# Restoring intracellular homeostasis disrupted by synthetic nanoassemblies

**DOI:** 10.1038/s41589-026-02279-x

**Published:** 2026-07-17

**Authors:** Jiaqi Xing, Xiaoran Zheng, Yong Ren, Maximilian Schuler, David Y. W. Ng, Tanja Weil, Seraphine V. Wegner, Christopher V. Synatschke

**Affiliations:** 1 Max Planck Institute for Polymer Research, Mainz, Germany; 2 Institute of Physiological Chemistry and Pathobiochemistry, University of Münster, Münster, Germany; 3 Max Planck School Matter to Life, Heidelberg, Germany

**Keywords:** Peptides, Synthetic biology

## Abstract

Cells have evolved to defend against perturbations by maintaining their intrinsic homeostasis to survive. However, no intrinsic pathway exists for them to expel new-to-biology synthetic nanostructures. Here we establish crosstalk between supramolecular transformations and genetic responses, achieving programmable influx–efflux cycles of nanoassemblies in living bacterial cells to restore redox and energy homeostasis. Specifically, a model photosensitizer–peptide conjugate undergoes multiple redox cycles between methionine and methionine sulfoxide (MetO), resulting in reversible morphological transformations between nanofibers (NFs) and nanoparticles (NPs). Upon irradiation, the oxidized peptide NPs are internalized into bacteria. To counteract the perturbations caused by internalized NPs, engineered bacteria activate the expression of MetO reductases in response to photo-oxidative stress. The internalized NPs are intracellularly enzymatically reduced such that they are expelled as reduced NFs, setting the stage for subsequent cycles. The concept presented here paves the way for the interlinked network between dynamic supramolecular assemblies and cellular regulatory behaviors.

**Figure:**
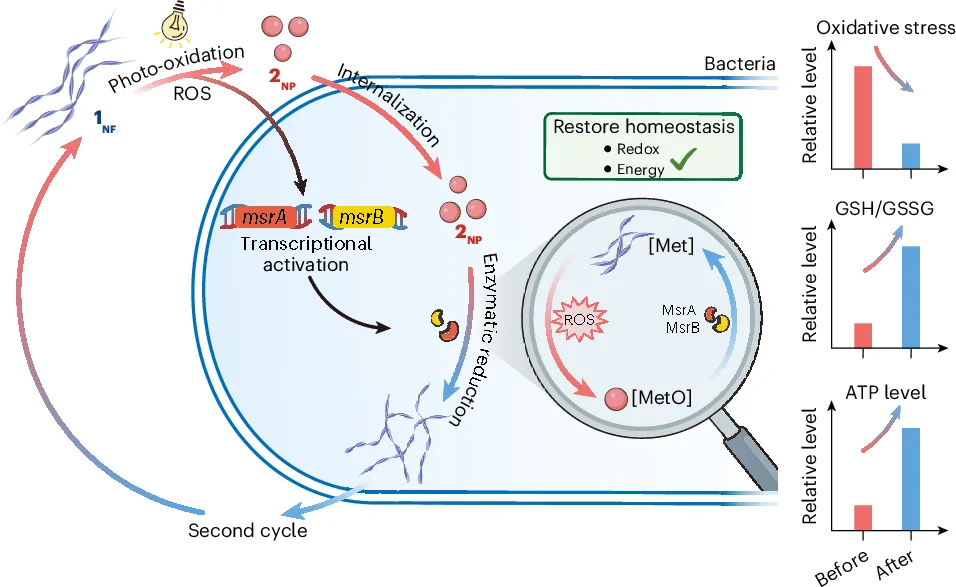

## Abstract (web summary)

Cells have evolved defense pathways to maintain homeostasis against external perturbations, but not against new-to-nature nanomaterials, which can irreversibly accumulate and induce cell stress. Now, programmable peptide nanoassemblies have been designed that can morphologically transform in influx–efflux cycles in response to restoring redox homeostasis in bacterial cells.

## Main

Over the course of millennia, cells have evolved sophisticated defense pathways to maintain their homeostasis against external perturbations^1^, which typically involve sequestering^2^, degradation^3^ and clearance of the toxins^4^. However, with the advent of nanotechnology for novel therapies^5^, cells are encountering new types of materials for which they have not yet developed coping mechanisms. In fact, many nanomaterials are developed to accumulate within cells to disrupt them as part of a therapeutic approach^6^. In general, because cells lack intrinsic pathways to remove these materials, persistent intracellular accumulation of nanomaterials occurs, resulting in the disruption of homeostasis^7,8^. This unintentionally hinders explorations of cooperative functionalities at biotic–abiotic interfaces. Notably, the development of nanomaterials capable of linking their molecular or morphological transformations to cellular regulatory pathways for restoring intracellular homeostasis remains elusive.

**Fig. 1:**
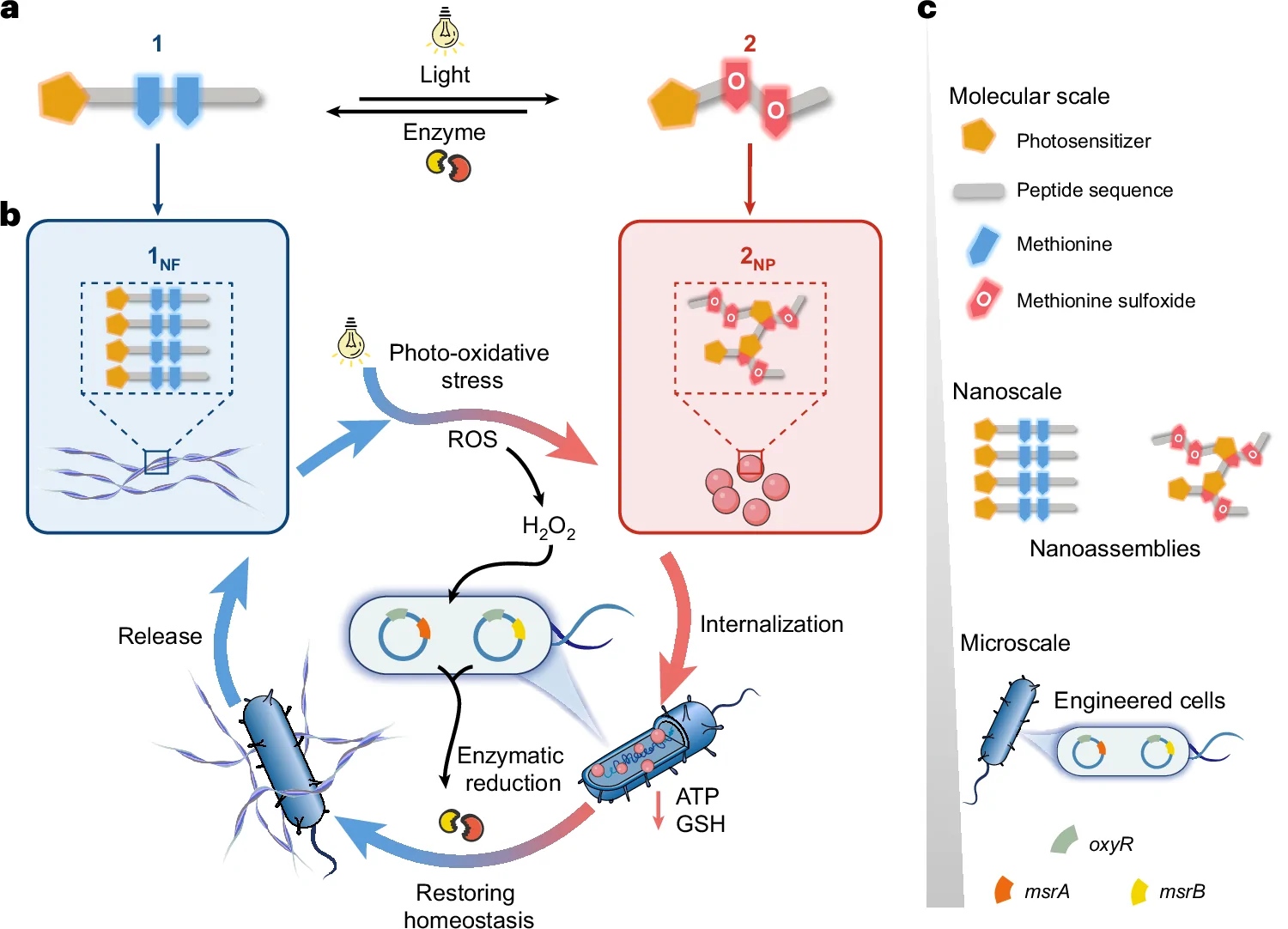
Schematic illustration of programmable influx–efflux cycles of nanoassemblies in living cells. **a**, Reversible redox-regulated reactions at the molecular level. **b**, Restoration of intracellular homeostasis disrupted by nanoassemblies, using engineered bacteria through an ROS/H_2_O_2_-initiated genetic circuit. **c**, Annotations of all symbols used in the scheme. OxyR is constitutively expressed and, in response to H_2_O_2_, activates transcription of *msrA* or *msrB*, thereby inducing expression of their encoded enzymes.

Self-assembled nanomaterials have been brought into contact with living cells as therapeutic agents^9,10^ as a result of recent advances in their dynamic nature arising from reversible, cumulative, noncovalent interactions^11–15^. Moreover, the precise control of nanomaterials through stimuli such as pH^16^, light^17,18^ and redox^19^ allows for switchable morphology, size and assembly states. The programmability and dynamics allow them to serve as precision building blocks that undergo programmed intracellular assembly and accumulate at the site of interest, thereby improving their specificity and pharmacokinetics^6,20^. However, intracellular self-assemblies often lead to irreversible accumulation and end-effect cellular responses for therapeutic applications^21–23^, including mechanical stress, homeostasis disruption and eventual cell death. As cells have not evolved intrinsic genetic programs to counteract synthetic assemblies, expelling accumulated nanostructures overwhelms cellular regulatory capacity. To modulate cellular response beyond cell death, the intracellular assembly systems need to link with living cell regulatory pathways for reversible accumulation. Here, we establish an integrated regulatory network linking supramolecular transformations with engineered genetic pathways and metabolic responses. This design enables self-assembled nanomaterials to be transiently internalized into engineered bacterial cells and subsequently expelled in response to intracellular cues, thereby restoring redox and metabolic homeostasis (Fig. 1).

As the starting point for self-regulated supramolecular assembly, we designed a self-assembling photo-oxidizable peptide containing the photosensitizer protoporphyrin IX (PPIX) and methionine (Met) residues. The amphiphilic DVMVMD sequence is rationally designed to combine reversible hydrogen bonding with spatially segregated hydrophobic residues. Upon irradiation with visible light (410 nm), PPIX generates reactive oxygen species (ROS)^24^, which oxidizes Met to Met sulfoxide (MetO), thereby rebalancing noncovalent forces driving a morphological transition from nanofibers (NFs) to spherical nanoparticles (NPs). At the same time, the oxidation of the peptide prompts NP uptake into bacterial cells and the generation of intracellular oxidative stress. We genetically engineer the bacteria to express MetO reductases in response to this oxidative stress as a coping strategy. The reductases counteract the internalized NPs, reducing them and expelling them from the bacterial cells to form extracellular NFs again. Overall, we demonstrate programmable influx–efflux cycles of synthetic nanoassemblies within living bacterial cells, empowering cells to defend against synthetic nanostructures through negative feedback loops that mediate crosstalk between supramolecular assemblies and cellular behavior.

## Reversible oxidation and reduction of peptide conjugates

**Fig. 2:**
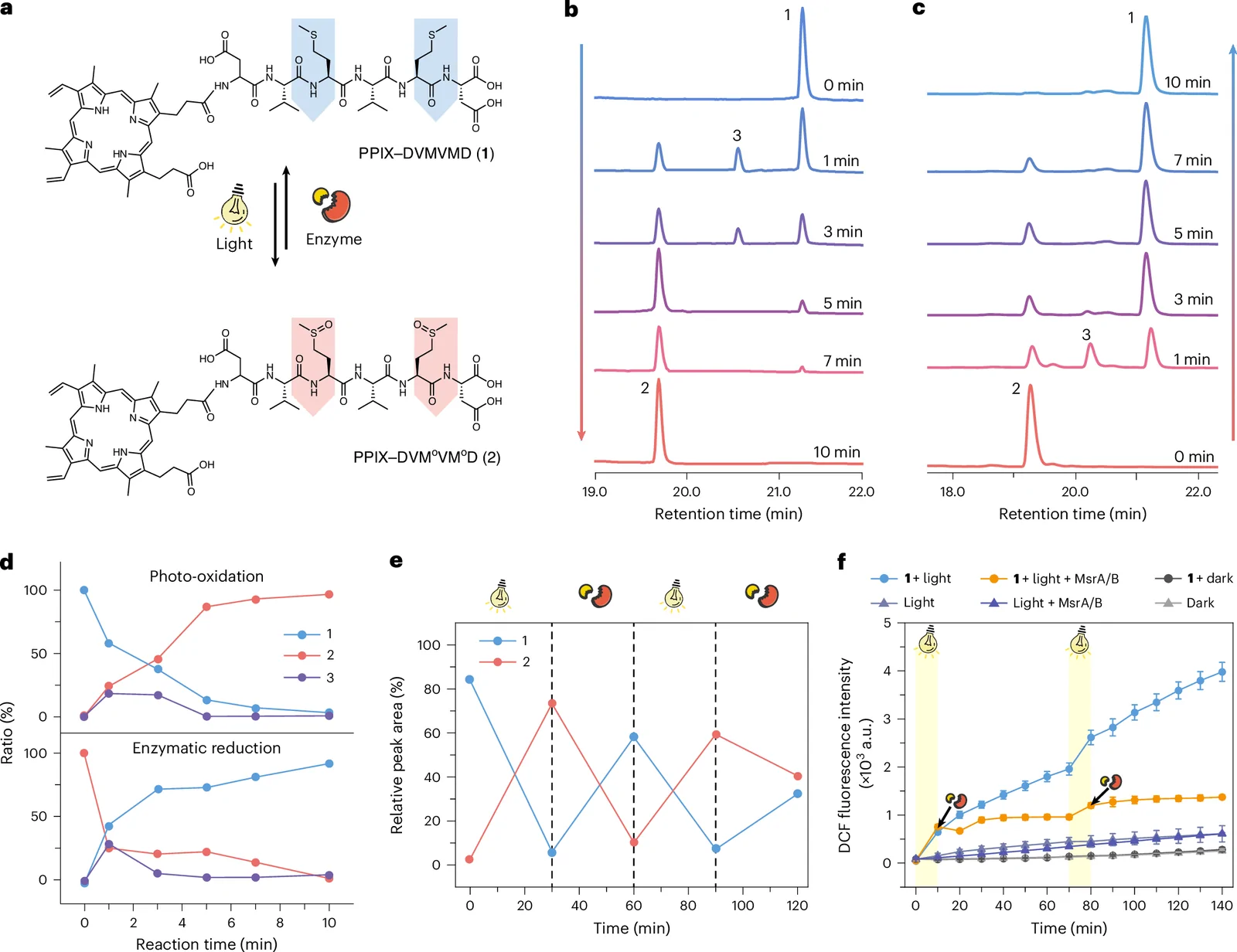
Reversible redox-mediated conversion between **1** and **2**. **a**, Reaction scheme for redox regulation conversion between compounds **1** and **2**. **b**, Kinetic analysis of cold-white-light-induced oxidation of compound **1** (200 µM in AB buffer). Samples were diluted with methanol to prevent aggregation before HPLC–MS injection (final concentration: 50 μM). **c**, Kinetic analysis over the CL of *E*. *coli* MsrA (0.5 mg) and *E*. *coli* MsrB (0.5 mg) enzymatic reduction of compound **2** (final concentration: 50 μM) using analytical HPLC. **d**, Molar ratio of compounds **1**, **2** and **3** quantified from peak integrations at 254 nm during oxidation progress and at 404 nm during reduction progress. **e**, Multiple redox-mediated cycles between compounds **1** and **2**. Relative peak area of compounds **1** and **2** in multiple cycles. **f**, The cyclic regulation of ROS generation by compound **1** (final concentration: 200 μM) and scavenging by MsrA and MsrB indicated by DCFH-DA. The black arrows indicate the point of enzyme addition and the yellow areas indicate the light irradiation for 10 min. Data were obtained from three independent replicates are presented as the mean ± s.d. Source data

First, we rationally designed the peptide PPIX–DVMVMD as model redox-active nanomaterial comprising a self-assembling peptide with two Met residues and an N-terminally conjugated PPIX. PPIX has been reported to generate ROS (^1^O_2_) upon irradiation with visible light (Supplementary Figs. 1 and 2)^25^. The reduced PPIX–peptide (compound **1**) and oxidized version with both Met residues oxidized to MetO (compound **2**) were synthesized by using standard, microwave-assisted Fmoc solid-phase peptide synthesis, followed by an on-resin coupling of PPIX and purification through high-performance liquid chromatography (HPLC) after cleavage (Supplementary Figs. 3–5). The irradiation of compound **1** (50 µM) with white light (3.0 mW cm^−2^) resulted in a quantitative conversion of compound **1** (*t*_R_ = 10.7 min) into the oxidized compound **2** (*t*_R_ = 10.3 min, 97%) over the course of 10 min, as followed by HPLC–mass spectrometry (MS) (Fig. 2a–d and Supplementary Fig. 6). The photo-oxidation proceeded through monooxidized intermediates (compound **3**, *t*_R_ = 10.5 min; Supplementary Fig. 7), which appeared briefly at 1 min of irradiation before being further oxidized to compound **2**. The photo-oxidation was 30-fold more efficient than the chemical oxidation of a non-PPIX-containing control peptide (50 µM DVMVMD) with 20 mM H_2_O_2_ (Supplementary Figs. 8 and 9). The high efficiency of the photo-oxidation presumably arises from the close proximity of the photosensitizer to the thioether groups (Supplementary Fig. 8) in compound **1**. When we compared the synthesized compound **2** (**2**_**syn**_) to its counterpart formed through the photo-oxidation of compound **1** (**2**_**irr**_), we observed no differences in their optical properties and stereochemistry (Supplementary Figs. 10–12). Thus, both compounds are hereafter designated as **2**. Next, we looked into the possibility of reducing the oxidized Met residues to close the reduction–oxidation loop. To this end, we generated cell lysates (CLs) from *Escherichia* *coli* cells, expressing the MetO reductases MsrA (26.8 kDa) and MsrB (18.9 kDa), which are able to reduce Met *S*-sulfoxide and *R*-sulfoxide, respectively (Supplementary Figs. 12 and 13)^26^. After we incubated compound **2** (*t*_R_ = 19.8 min) with the CLs supplemented with dithiothreitol (DTT) for 1 min at 37 °C, two new peaks corresponding to compound **3** (*t*_R_ = 20.2 min) and compound **1** (*t*_R_ = 21.2 min) appeared (Fig. 2c,d).

After 10 min of reaction, 88% of compound **2** was converted to compound **1**. Following a successful single oxidation–reduction cycle, we probed the ability of compound **1** to undergo repeated redox cycles by exposing the PPIX–peptide to light for 30 min, followed by enzyme incubation for another 30 min (Fig. 2e and Supplementary Fig. 14). Starting with light irradiation of compound **1**, compound **2** gradually became the dominant species. The addition of CLs resulted in the reduction to compound **1**. A similar conversion from compound **1** to compound **2** was observed after the second illumination cycle and a conversion to compound **1** was observed after the addition of enzymes. A gradual decrease in the peak area of all compounds was observed after each step and attributed to product loss during the workup before chromatographic analysis (Supplementary Fig. 15). Having validated the redox cycle, we investigated the connection between ROS generation and scavenging. Under repeated illumination, the compound consistently generated ROS in a stepwise manner, detected by a general ROS probe, 2′,7′-dichlorofluorescein diacetate (DCFH-DA). In contrast, with the addition of reductases the elevated ROS level could be effectively buffered and the fluorescence signal was maintained at a stable plateau throughout both illumination cycles (Fig. 2f and Supplementary Fig. 16). These results indicate that the reductases actively counteract photoinduced ROS accumulation, maintaining redox homeostasis during repeated illumination.

## Redox state-dependent morphological transformations

**Fig. 3:**
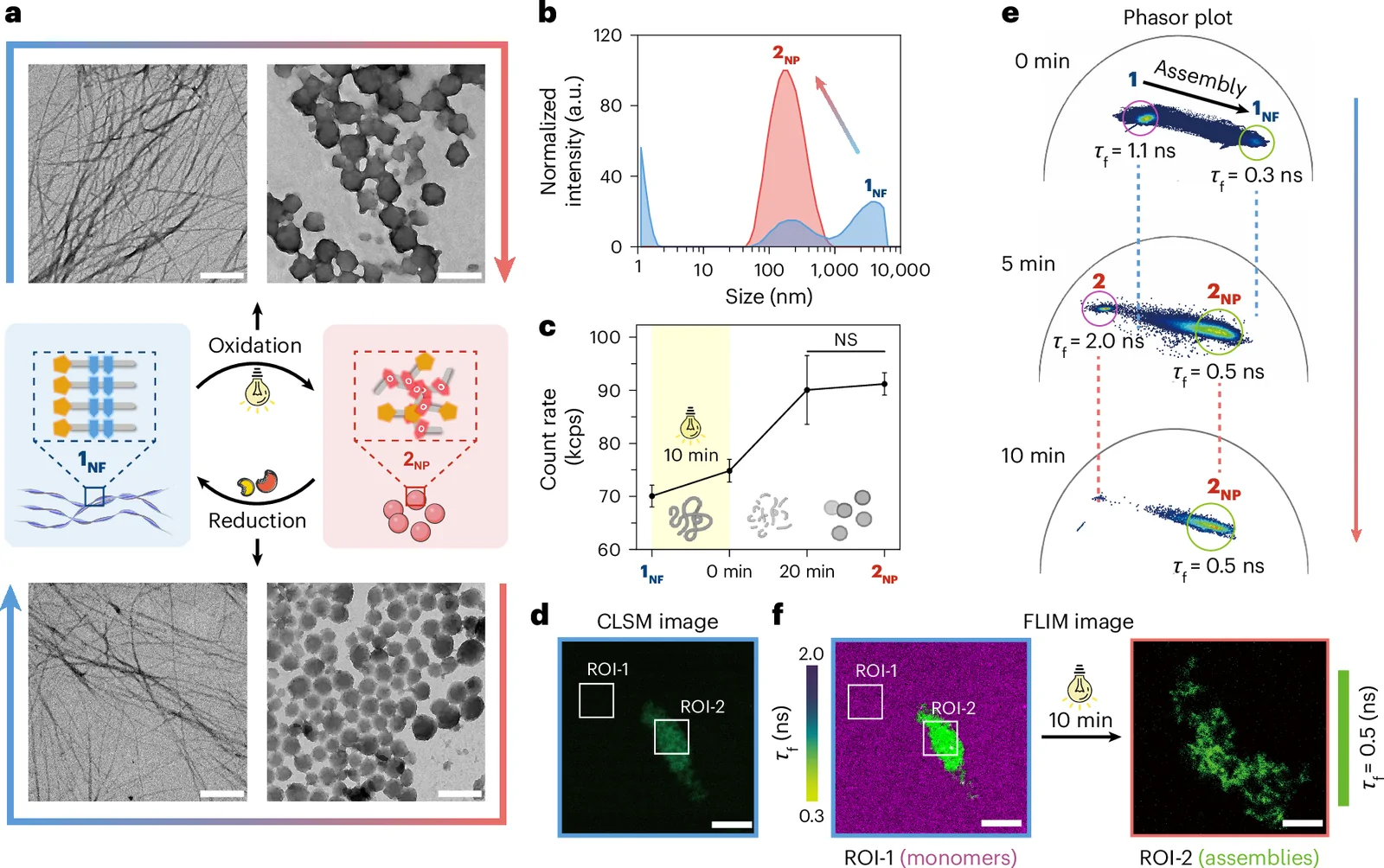
Analysis of cyclic morphological transformations and monitoring the progression. **a**, TEM images during different periods of the redox cycle. Top, the oxidation-induced morphological transformation from **1**_**NF**_ (200 μM) in AB buffer to **2**_**NP**_. Scale bar, 400 nm. Bottom, schematic illustrations of dynamic morphological transformations. The third row shows the reduction-induced morphological transformation from NPs **2**_**NP**_ (200 μM) in AB buffer to NFs **1**_**NF**_. Scale bar, 400 nm. **b**, DLS intensity-based size distribution histograms of **1**_**NF**_ and **2**_**NP**_. **c**, In situ monitoring of the morphological transformation from NFs to NPs, determined by the scattered light intensity in DLS. Data were obtained from three independent replicates and are presented as the mean ± s.d. Statistical significance was assessed using a one-way analysis of variance with Tukey’s multiple-comparison test. NS, not significant (*P* = 0.9809). Elements of this panel created in BioRender; Xing, J. https://BioRender.com/hlrh1rh (2026). **d**, Confocal fluorescence image for **1**_**NF**_ at 0 min (λ_ex_ = 488 nm, λ_em_ = 600–700 nm). Scale bar, 5 μm. **e**, Fluorescence lifetime analysis of **1**_**NF**_ assembly progression in a cell-free system using phasor plot analysis. Two major species showing **1** *τ*_f_ = 1.1 ns and **1**_**NF**_ *τ*_f_ = 0.3 ns can be identified. After 5 min of light irradiation, multiscale transformations are shown in two major species. The **1**_**NF**_ *τ*_f_ = 0.3 ns photons disappeared and two new photon populations of *τ*_f_ = 2.0 ns and 0.5 ns could be identified as **2** and **2**_**NP**_, respectively. After 10 min of light irradiation, only one major species showing **2**_**NP**_ *τ*_f_ = 0.5 ns was found. The magenta circles indicate monomers and the green circles indicate assemblies. **f**, Corresponding FLIM images for **1**_**NF**_ at 0 min and after 10 min of illumination (λ_ex_ = 488 nm). The channels are correlated with the circles in phasor plots. At 0 min, the magenta channel indicates monomers (**1** *τ*_f_ = 1.1 ns, ROI-1) and the green channel indicates assemblies (**1**_**NF**_ *τ*_f_ = 0.3 ns, ROI-2). Upon 10 min of irradiation, only assemblies (**2**_**NP**_ *τ*_f_ = 0.5 ns) can be seen. Scale bar, 5 μm. Experiments in **a**,**d**–**f** were independently repeated three times with similar results. Source data

After confirming the repeated cycling of oxidation states in the PPIX–peptide conjugates, we investigated how the oxidation state influences their self-assembly into nanostructures. To this end, we determined the optical properties, secondary structure and morphology of compounds **1** and **2** in nanoscale assemblies. To distinguish between monomeric and assembled states, we determined the critical aggregation concentrations of compounds **1** and **2** to be 59.4 µM and 114.7 µM, respectively (Supplementary Fig. 17). Thereafter, we used 200 µM peptides in all experiments to ensure the formation of nanoassemblies. Analysis of the absorption and fluorescence spectra acquired below and above the CAC showed that irradiation induces a red shift in the Soret band (*π* → π*) and emission spectra, together with broadening of the Q bands^27^. These spectral signatures indicate the transition of assembly states from predominantly H-aggregate to J-aggregate packing^28^ by photo-oxidation, which increases local polarity and reorganizes packing density (Supplementary Fig. 18). Transmission electron microscopy (TEM) revealed distinct morphologies for the assemblies formed from compounds **1** and **2**; in the reduced state, NFs several hundred nanometers long were found (**1**_**NF**_), whereas, in the oxidized state, spherical NPs (**2**_**NP**_) with a diameter of 200 nm were observed (Fig. 3a,b). Remarkably, both morphologies were reversibly interconvertible through photo-oxidation (**1**_**NF**_ → **2**_**NP**_) and enzymatic reduction (**2**_**NP**_ → **1**_**NF**_).

Furthermore, the changes in assembly size during the photo-oxidation of compound **1** to compound **2** were followed by using dynamic light scattering (DLS). The size distribution curve of **1**_**NF**_ showed several broad peaks with a large population above 1,000 nm, as is expected for NFs with various lengths. Upon photo-oxidation, with the emergence of **2**_**NP**_, the size distribution became much narrower, culminating in a single peak with a hydrodynamic diameter around 192 ± 28 nm (Fig. 3b and Supplementary Fig. 19), consistent with TEM measurements. In addition, the dynamic transition of **1**_**NF**_ to **2**_**NP**_ could also be followed using the derived count rate of scattered light from DLS measurements^29^. Initially, **1**_**NF**_ showed a count rate of 69 ± 1.67 kcps. After 10 min of illumination followed by 20 min of incubation for morphological transformation, the count rate increased and reached a value of 90 ± 5.29 kcps, similar to that of **2**_**NP**_ (92 ± 1.71 kcps) formed from the chemically synthesized compound (Fig. 3c). To capture the intermediate state of the transformation, TEM samples were prepared after 10 min of incubation, revealing the coexistence of NFs and NPs, which supports a stepwise transition rather than an abrupt two-state process (Supplementary Fig. 20). The peptides underwent changes to their secondary structure when transitioning from **1**_**NF**_ to **2**_**NP**_, as evaluated using Fourier-transform infrared (FT-IR) and circular dichroism (CD) spectroscopy. The FT-IR spectrum of **1**_**NF**_ showed a typical peak of α-helices at 1,649 cm^−1^ in the amide I region^30^, which was absent in **2**_**NP**_ (Supplementary Fig. 21). In the CD spectrum, a shoulder peak appeared at ~220 nm after illuminating **1**_**NF**_, indicating a β-sheet-rich secondary structure of the formed **2**_**NP**_ (ref. ^31^). At the same time, the CD spectrum of **1**_**NF**_ in the Soret region displayed a strong negative peak at 380 nm and a positive peak at 426 nm, indicating tightly packed neighboring porphyrins^32,33^. After irradiation, the intensity of peaks in the Soret region gradually decreased, indicating that this close packing was disturbed during the transformation by more hydrophilic MetO and the formation of NPs (Supplementary Fig. 22).

Next, we conducted confocal laser scanning microscopy (CLSM) and observed the presence of large aggregates with a high intensity of fluorescence signals (Fig. 3d). However, this method cannot distinguish between monomeric (**1** and **2**) and assembled states (**1**_**NF**_ and **2**_**NP**_). Thus, we performed phasor fluorescence lifetime imaging (phasor-FLIM) to measure fluorescence lifetime (*τ*_f_), which can be correlated to the molecular self-assembly^21,34^. First, we conducted control experiments with only compounds **1** and **2** in M9 medium, which was later also used for cell experiments (Fig. 3e,f and Supplementary Fig. 23). The assembly progression showed that both nonassembled compounds (**1**, *τ*_f_ = 1.1 ns and **2**, *τ*_f_ = 2.0 ns) had a longer *τ*_f_ than their assemblies (**1**_**NF**_, *τ*_f_ = 0.3 ns and **2**_**NP**_, *τ*_f_ = 0.5 ns). Following the successful identification of the initial and final states, we monitored *τ*_f_ alterations in phasor plots to track the dynamic progression over the course of photo-oxidation and related morphological transformation. After 3 min of irradiation, a new photon population of **2**_**NP**_ (*τ*_f_ = 0.5 ns) was detected (Supplementary Fig. 24). Extending the illumination time to 5 min, the population corresponding to **1** declined, along with the emergence of the new photon population of **2** (*τ*_f_ = 2.0 ns), indicating the conversion of **1** to **2**. Regarding assemblies, the **1**_**NF**_ characteristic phasor completely disappeared at 5 min, indicating potential disassembly. By separating the lifetimes of emitted photons on a phasor plot (Fig. 3e), we were able to gather the population of photons corresponding to their spatial distribution in FLIM images (Fig. 3f)^35^. The magenta signals of free molecules (region of interest 1, ROI-1) gradually decreased over time, suggesting the progression of assembly (Supplementary Fig. 23). Finally, after prolonged illumination of up to 10 min, the photon populations exhibited only one phasor of **2**_**NP**_ (*τ*_f_ = 0.5 ns) along with solely green signals from assemblies, indicating the complete transition of free monomers into **2**_**NP**_. In summary, from the observations above, we hypothesize that, because of the photo-oxidation of compounds **1** to **2**, **1**_**NF**_ first fragments into smaller assemblies, which then reassemble into spherical NPs, **2**_**NP**_ (Fig. 3b–f).

## Interaction of nanoassemblies and bacterial cells

**Fig. 4:**
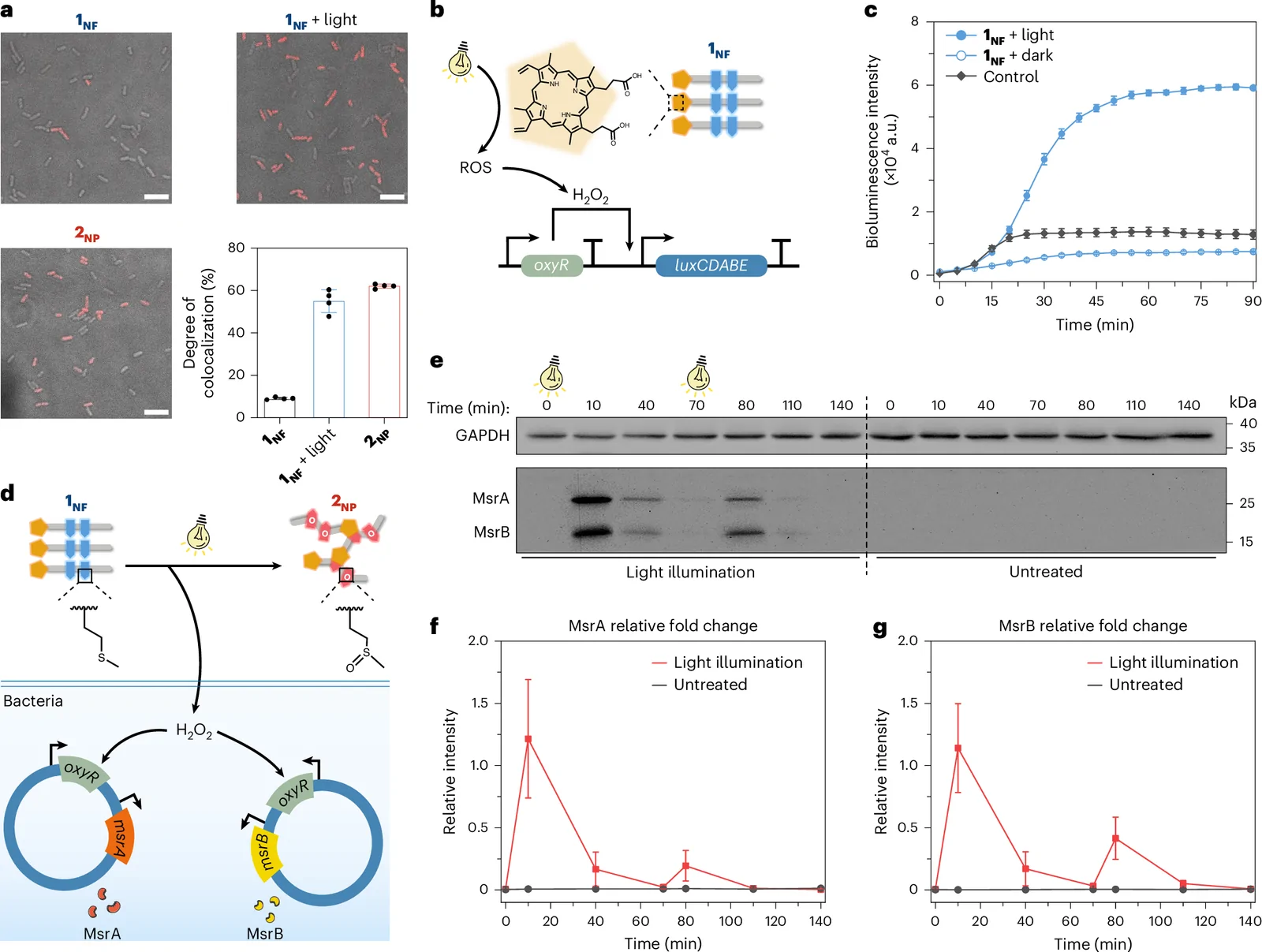
Programming bacterial responses to photo-oxidative stress. **a**, CLSM images of *E*. *coli* GFP bacterial cells treated with **1**_**NF**_, **1**_**NF**_ + light or **2**_**NP**_ (all at 200 µM) after 8 h. Scale bars, 4 μm. The histogram shows the average colocalization degree of three groups evaluated by CLSM images. The mean of *n* = 4 biological samples ± s.d. is shown. Experiments were independently repeated three times with similar results. **b**, Schematic representation of the *oxyR-lux* gene activation induced by **1**_**NF**_. The emergent production of ^1^O_2_ elevates the H_2_O_2_ level, which in turn enables the constitutively expressed OxyR to activate downstream transcription of the synthetic gene cluster luxCDABE in the presence of H_2_O_2_, leading to a bacterial bioluminescence response. **c**, *E*. *coli* MG1655, transformed with the *oxyR-lux* plasmid, was incubated with AB buffer without **1**_**NF**_ as a control and with **1**_**NF**_ under either dark or light conditions. Bioluminescence intensities were determined by Tecan. Data were obtained from three independent replicates and are presented as the mean ± s.d. **d**, Schematics of multiactivation of MsrA and MsrB in response to photo-oxidative stress. **e**, Immunoblots showing the expression of MsrA and MsrB in *E*. *coli* MG1655 upon light irradiation for 10 min at the indicated time points. **f**,**g**, Quantification of the band intensities of MsrA (**f**) and MsrB (**g**) from the immunoblots in **e**. The red line indicates samples with 10 min of light illumination at 0 and 70 min. The gray line indicates untreated samples used as controls. The mean of *n* = 3 biological samples ± s.d. is shown. Source data

The next question to be addressed was the interaction of the nanomaterials **1**_**NF**_ and **2**_**NP**_ with bacterial cells. To this end, *E*. *coli* MG1655 (expressing green fluorescent protein (GFP) for visualization, named *E*. *coli* GFP) were incubated with **1**_**NF**_ and **2**_**NP**_ for 12 h. Bacterial colony-counting assays showed no toxicity of the compounds up to 400 µM and no phototoxicity for up to 15 min of illumination (Supplementary Fig. 25). CLSM images showed that only **2**_**NP**_ but not **1**_**NF**_ was able to enter the bacteria, as visualized using PPIX fluorescence (Fig. 4a). Moreover, the better internalization of **2**_**NP**_ compared to that of **1**_**NF**_ was further confirmed by the percentage of PPIX-positive and GFP-positive cells in fluorescence-activated cell sorting (FACS) analysis (**2**_**NP**_, 25.35% ± 2.35% and **1**_**NF**_, 4.35% ± 0.11%; Supplementary Figs. 26–29). Remarkably, when the bacteria incubated with **1**_**NF**_ were exposed to light, peptide internalization greatly increased to comparable levels observed for **2**_**NP**_. To investigate the possible pathway of the preferential uptake of **2**_**NP**_ over **1**_**NF**_, we evaluated NP internalization under conditions that suppressed cellular energy production. Both low-temperature incubation (4 °C) and addition of an ATP synthase inhibitor (*N*,*N*′-dicyclohexylcarbodiimide, DCCD) (Supplementary Figs. 30 and 31) were tested. In both cases, the relatively low internalization rate shown in CLSM and FACS indicated that internalization is metabolically driven rather than passive^36^. We next assessed whether transport could be attributed to membrane damage. By using propidium iodide (PI), a membrane-impermeable DNA-binding dye, we observed no notable increase in PI uptake under all tested conditions (Supplementary Fig. 32). These results indicate that membrane permeability and structural integrity remain intact^37^ and the observed influx–efflux behavior is not attributable to nonspecific membrane leakage or membrane disruption.

## Programming bacteria response to intracellular nanoassemblies

To equip the bacteria with the means to expel the NPs, we engineered them with a regulatory gene circuit, which is activated upon oxidative stress. As repeated illumination perturbed ROS levels (Fig. 2f and Supplementary Fig. 16), we hypothesized that this oxidative stress could also be used for the regulation of redox sensitive transcription factor and induce the expression of regulated genes.

To establish the link between this photo-oxidative stress and the cellular response, we introduced to the bacteria a plasmid that constitutively expresses the oxidative stress-related transcription factor OxyR, which can then turn on genes in the presence of H_2_O_2_ as part of the oxidative stress response^38,39^ (Fig. 4b). Initially, we regulated with OxyR the expression of the bacterial luciferase gene cluster, *luxCDABE*, from *Photorhabdus* *luminescens*^40^ (denoted as *oxyR-lux*) as a bioluminescent reporter; later, we regulated the expression of the MetO reductases, MsrA/MsrB (that is, *oxyR-msrA* and *oxyR-msrB*), to reduce the MetO-containing **1**_**NP**_.

First, to demonstrate that oxidative stress upon illumination can activate the gene circuit, we incubated *E*. *coli* MG1655 transformed with *oxyR-lux* with **1**_**NF**_ followed by light irradiation for 10 min. These bacteria exposed to light exhibited bioluminescence; however, in the dark, regardless of **1**_**NF**_ addition, no bioluminescence was observed (Fig. 4c). The bioluminescence signal from light-exposed bacteria was comparable to *oxyR-lux* bacteria that were exposed to 10 µM exogenous H_2_O_2_ (Supplementary Fig. 33). These results demonstrated that ROS generated by **1**_**NF**_ under light produces sufficient H_2_O_2_ in the cells to activate OxyR-regulated gene expression, thereby establishing communication between the living cells and synthetic materials through photo-oxidative stress.

Subsequently, we aimed to leverage the cellular stress response induced by **1**_**NF**_ upon irradiation to facilitate the elimination of nanoassemblies from the cell (Fig. 4d), thereby enabling engineered bacteria to establish a homeostatic mechanism to counteract disruption caused by intracellular **2**_**NP**_. To this end, we regulated the expression of MsrA and MsrB under the control of OxyR. When these engineered bacterial cells were incubated with **1**_**NF**_ and exposed to light, they expressed MsrA and MsrB in comparable amounts, as shown by western blot analysis (Fig. 4e). The rapid increase in enzyme expression levels after 10 min of light exposure was followed by a gradual degradation in the dark over the next 60 min. Notably, a second 10-min illumination induced enzyme production again, albeit at a lower level (Fig. 4f,g). A positive control with 10 μM H_2_O_2_ added at 0 and 70 min showed similar enzyme production and degradation (Supplementary Fig. 34). Additionally, the bacterial colony-counting assay confirmed that the nanoassemblies do not affect bacterial viability throughout the repeated illumination cycles (Supplementary Fig. 35). Thus, the programmed OxyR-regulated gene circuits effectively regulated the cellular response to the photo-oxidative stress from the NPs.

## Restoring intracellular homeostasis

Having established the enzymatic response of the bacterial cells to photo-oxidative stress, we evaluated how these enzymes act on intracellular nanoassemblies and whether they are able to remove them from the cells to reestablish homeostasis. Initially, both monomer **1** (*τ*_f_ = 1.1 ns) and **1**_**NF**_ (*τ*_f_ = 0.4 ns) could only be found extracellularly, not inside *E*. *coli* MG1655 (cotransformed with *oxyR-msrA* and *oxyR-msrB*, named *E*. *coli* AB), as verified by phasor-FLIM (Supplementary Fig. 36). Consistent with these results, when exposed to light for 10 min, **1**_**NF**_ was transformed into **2**_**NP**_ (*τ*f = 0.8 ns) and subsequently internalized into the bacteria (Supplementary Figs. 36 and 37). Yet, with enzymes designed to counteract the intracellular **2**_**NP**_, the reduced **1**_**NF**_ was expelled from bacteria after 70 min, indicating that homeostasis was successfully restored.

**Fig. 5:**
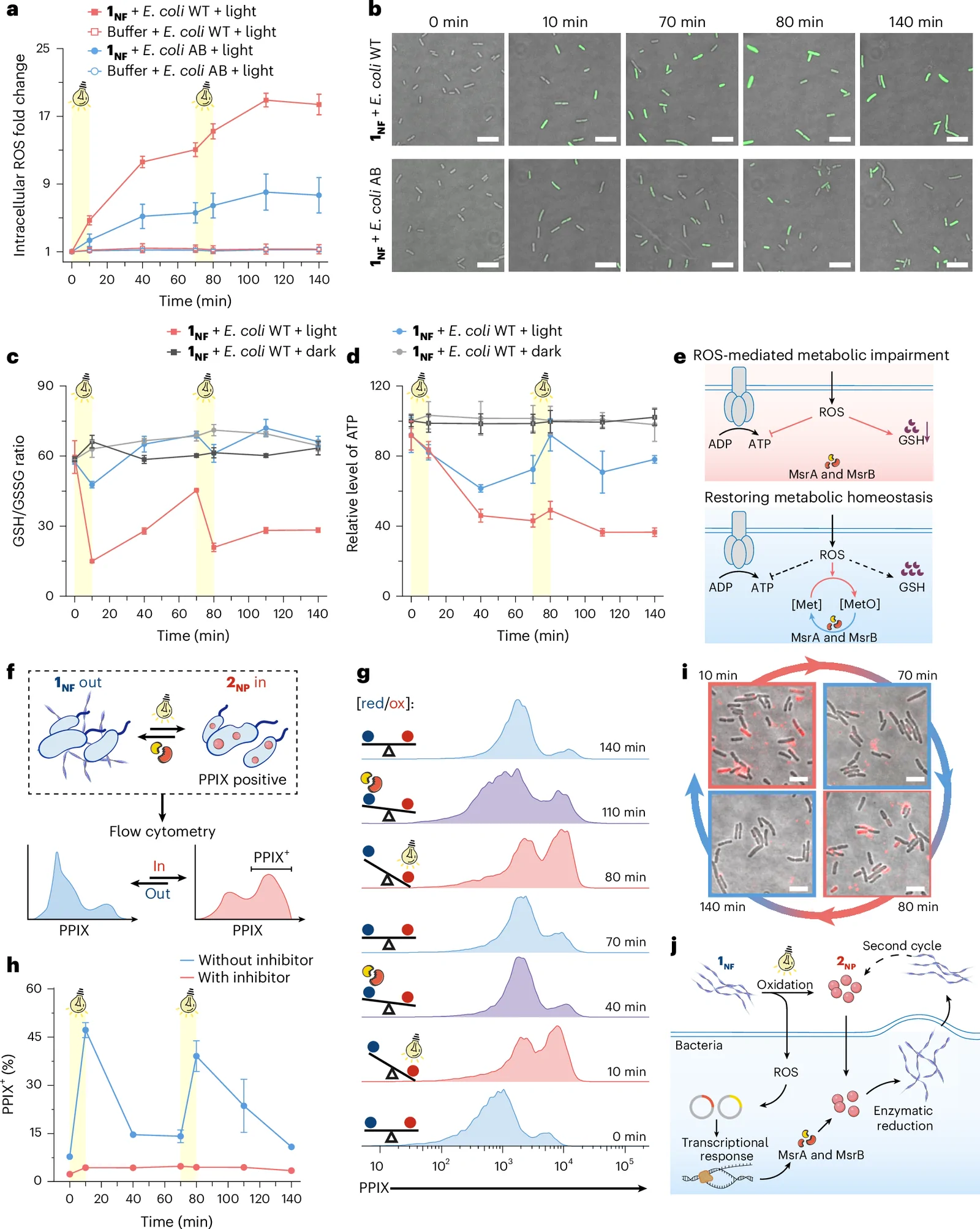
Restoration of intracellular homeostasis. **a**, Time-course analysis of intracellular ROS levels using the DCFH-DA indicator (relative to the dark-treated control groups). The mean of *n* = 6 biological samples ± s.d. is shown. Light exposure (yellow shading) was applied for 0–10 min and 70–80 min for all experiments in **a**–**i**; all other time points were incubated under dark. **b**, Representative fluorescence imaging of **1**_**NF**_-treated *E*. *coli* (WT and AB) under light exposure. Bacterial cells are shown in gray. ROS levels were probed by DCFH-DA (green). Scale bar, 10 μm. **c**, Evaluation of redox status by GSH/GSSG ratio. The mean of *n* = 3 biological samples ± s.d. is shown. **d**, Relative intracellular ATP levels in *E*. *coli* WT and AB following **1**_**NF**_ treatment under light or dark conditions (relative to the dark-treated control groups). The mean of *n* = 6 biological samples ± s.d. is shown. **e**, Schematics for the ROS-induced metabolic impairment (top) and restoration of intracellular redox and energy homeostasis in *E*. *coli* AB (bottom). **f**, Schematic illustration of the restoration of the homeostasis process in *E*. *coli* AB induced by the redox cycle of **1**_**NF**_ and **2**_**NP**_, as mapped by flow cytometry. **g**, Representative flow cytometry histogram indicates restoration of intracellular homeostasis over two cycles, with gating at Hoechst^+^ (gating strategies in Supplementary Fig. 39). **h**, Line graph showing the percentage of PPIX-positive bacterial cells at different time points with and without ATP synthase inhibitor, DCCD, quantified by flow cytometry gating at Hoechst^+^. The mean of *n* = 3 biological samples ± s.d. is shown. **i**, CLSM images of two cycles at different time points and stages. PPIX signals are shown in red. Scale bar, 5 μm. **j**, Schematics for the restoration of intracellular homeostasis disrupted by nanoassemblies. Experiments shown in **b**,**i** were independently repeated three times with similar results. Source data

To specify the pathways disrupted by the intracellular nanostructures, we first evaluated the intracellular redox fluctuations caused by photo-oxidative stress. Intracellular ROS probe DCFH-DA confirmed that *E*. *coli* AB could substantially lower the concentration of ROS and effectively attenuate the oxidative stress (Fig. 5a,b). In comparison, the *E*. *coli* wild type (WT; without any plasmid) cultured with **1**_**NF**_ under illumination showed 2.7-fold stronger DCF fluorescence after 140 min, indicating that *E*. *coli* AB effectively prevented the intracellular ROS accumulation. Presumably, ROS generated by peptide-conjugated PPIX is rapidly consumed by the Met residues (Met → MetO)^41^. Subsequently, reductases, as antioxidant repair enzymes, continuously regenerate Met (MetO → Met)^42^, thereby replenishing the intracellular antioxidant pool and preventing excessive ROS accumulation^43,44^.

As cells possess intrinsic regulatory pathways to buffer moderate oxidative stress, we next examined whether the ROS levels generated here exceeded this regulatory range (Fig. 5c). The most important redox buffer in cells is the antioxidant glutathione (GSH), which converts into oxidized glutathione (GSSG) under oxidative stress. The GSH/GSSG ratio is a commonly used sensitive early biomarker of the redox homeostasis. Measuring the GSH/GSSG ratio in the bacterial cells, the *E*. *coli* AB cells exposed to **1**_**NF**_ and light were able to maintain the same ratio as the controls in the dark. In contrast, this ratio dropped notably in *E*. *coli* WT in the presence of **1**_**NF**_ after each light exposure, where the ratio recovered slowly after the first light pulse but failed to recover after the second illumination cycle. These measurements reflect that the nanoassemblies under light are able greatly damage the redox buffer and the OxyR-regulated expression of MsrA and MsrB effectively counteracts this oxidative damage.

Lastly, we evaluated the perturbation on the ATP levels in the cells throughout the influx–efflux cycles (Fig. 5d). Consistent with the elevated ROS accumulation, at 140 min, ATP in the **1**_**NF**_ + *E*. *coli* WT + light group dropped to 36.5% ± 2.5% of the initial level, indicating that ROS accumulation disrupted metabolism and impaired ATP synthesis. In contrast, the **1**_**NF**_ + *E*. *coli* AB + light group showed a milder initial decrease in ATP levels, followed by a recovery that stabilized around 80% after 140 min, indicating that the reductases could counteract the redox stress of **1**_**NF**_.

Taken together, these results indicated that, although photo-oxidative stress from **1**_**NF**_ results in oxidative stress, *E*. *coli* AB cells were able recover from this stress better than *E*. *coli* WT (Fig. 5e).

After identifying that metabolic homeostasis was restored, we used flow cytometry to quantify the influx–efflux cycles. Upon 10 min of illumination, 45.4% ± 3.6% of cells were PPIX positive because of intracellular **2**_**NP**_ (Fig. 5f,g and Supplementary Figs. 38 and 39). After 40 min, the number of PPIX-positive cells decreased to 14.4% ± 0.6%, indicating the removal of **2**_**NP**_ from the bacteria through intracellular enzymatic reduction of MetO. Consequently, after 70 min, reduced **1**_**NF**_ was expelled from bacteria, successfully establishing a closed loop. With another 10 min of light exposure, the extracellular **1**_**NF**_ was reoxidized to **2**_**NP**_ and detected intracellularly. During this second cycle, **2**_**NP**_ was removed again through the action of the activated MsrA and MsrB expression in the engineered bacteria. However, no PPIX signal was observed in the presence of an ATP synthase inhibitor (Fig. 5h), indicating that the uptake of nanoassemblies is an active and not a passive process. These observations with FACS analysis were confirmed by CLSM imaging, which showed clear PPIX fluorescence in the bacteria after each light exposure and the disappearance of this signal after some time (Fig. 5i). In addition, at different time points, bacterial lysate was collected and analyzed by analytical HPLC–MS to verify the presence of compounds within the regulatory feedback loops (Supplementary Fig. 40). Following enzymatic reduction and elimination of intracellular NPs, the bacteria reverted to their initial state and were ready to undergo the subsequent redox cycles (Fig. 5j). This design provides a blueprint for establishing regulatory feedback loops for bacterial cells to restore the intracellular redox and energy homeostasis by counteracting synthetic nanoassemblies.

## Discussion

Here, we established a closed loop between supramolecular transformation and genetic response, enabling influx–efflux cycles of synthetic nanostructures within living cells, during which intracellular redox and energy homeostasis were restored. The bottom-up design started from a molecular redox cycle coupled with the formation of distinct nanostructures, namely NFs and NPs. The assembly progression and morphological transformation were monitored by phasor-FLIM as a function of distinguishing *τ*_f_ at different stages of the cycle. In combination with DLS measurements, the mechanism of reversible morphological transitions between reduced NFs and oxidized NPs was elucidated. Moreover, the two morphologies showed strong differences in their interaction with cells. NPs were found to be efficiently internalized compared with NFs. Once accumulated within the cells, the invasive nanoassemblies cannot be expelled through an intrinsic pathway. Herein, we programmed bacteria to leverage the cellular stress response induced by **1**_**NF**_ upon irradiation to regulate enzyme expression to counteract invasive NPs. Specifically, upon irradiation, **1**_**NF**_ transformed to **2**_**NP**_, causing intracellular ROS perturbations to exceed the *E*. *coli* WT regulatory range. Meanwhile, photo-oxidative stress activated the biosynthesis of enzymes to counteract intracellular **2**_**NP**_ and expel **1**_**NF**_. This, in turn, marked the inception of the second loop, which was triggered by subsequent light illumination and repeated activation of the negative feedback loop in cells, thereby restoring homeostasis.

Recently, the integration of dynamic self-assemblies within a biological context, such as intracellular self-assembly, has become a promising strategy in responsive nanomedicine and delivery. Previous reports have demonstrated light-triggered or enzyme-triggered peptide assemblies^7,21,45^ for end-effect cellular responses. While this unidirectional behavior effectively destroys cell metabolism for tumor suppression, it poses challenges because of the absence of coupling between the intracellular nanostructures and endogenous feedback mechanisms, thereby hindering their translation to scenarios requiring sustained cellular viability and precise, nondestructive function modulation, including probiotic interventions^46^, programmable cell-based therapeutics^47^ and engineered living materials^48^. In contrast, our design, by introducing a negative feedback loop, enabled bacterial cells to defend against synthetic nanostructures. Specifically, the PPIX–peptide conjugate featured photo-oxidizable Met residues that coupled to cellular redox processes, thereby enabling reversible morphological transformations between **1**_**NF**_ and **2**_**NP**._ Furthermore, the incorporation of Met residues also functioned as redox modulators that restored the metabolic homeostasis^41,43^. Consequently, the metabolic restoration triggered the feedback-driven expulsion of nanoassemblies. This design allows assembly transformations to be directly coupled to cellular metabolic processes rather than merely introducing a static nanostructure to cells, opening conceptual pathways at the interface of biology and materials science.

Moving forward, however, translating this approach to mammalian systems requires the strategic integration of feedback-responsive materials that harmonize with, rather than disrupt, complex endogenous regulatory mechanisms. One promising direction is to repurpose metabolic cues as both assembly triggers and cellular pathway regulators, enabling bidirectional communication between supramolecular nanomaterials and cellular responses. The strategy holds substantial potential for next-generation nanomedicine, featuring minimized off-target effects and toxicity, alongside optimized kinetics. Overall, this work fosters a broader understanding of biotic–abiotic interfaces, thereby redefining the design of adaptive, self-regulating systems with lifelike functionality.

## Methods

### Microwave-assisted peptide synthesizer

Peptides were synthesized using a Liberty Blue automated microwave peptide synthesizer (CEM) at a 0.1-mmol scale using Fmoc-protected Wang resin according to the standard coupling strategy. Briefly, the resin was swollen in DMF for 1 h and, subsequently, the Fmoc protecting group was cleaved with a piperidine solution (20 vol% in DMF) by microwaving at 155 W, 75 °C for 15 s and at 30 W, 90 °C for 50 s. The resin was washed three times with DMF and Fmoc-protected amino acid (5 equiv. relative to the resin loading capacity), DIC (5 equiv.) and Oxyma Pure (10 equiv.) dissolved in DMF were added to the reaction vessel. After microwaving at 170 W, 75 °C for 15 s and 30 W, 90 °C for 110 s, the resin was washed with DMF. Repeating this procedure for all required amino acids yielded the desired peptide sequence.

### Photo-oxidation kinetic analysis

The peptide conjugates were dissolved in methanol at high concentrations and then diluted with AB buffer (Supplementary Table 1) to a final concentration of 200 µM (2% methanol). The samples were incubated overnight at 37 °C to allow nanostructure formation. Before analytical HPLC injection, four volumes of methanol were added to ensure complete solubilization (final concentration: 50 µM).

### Enzymatic reduction kinetic analysis

The peptide conjugates were dissolved in methanol at high concentrations and subsequently diluted with AB buffer to a final concentration of 200 µM (2% methanol). The samples were incubated overnight at 37 °C to allow nanostructure formation. For enzymatic reduction, either purified enzymes or CLs were added and the mixtures were incubated supplement with 500 µM DTT at 37 °C. At each time points, four volumes of methanol were added to precipitate proteins, enzymes and other high-molecular-weight components. Following centrifugation at 16,200*g* for 20 min at 4 °C, the supernatant was collected and directly injected into the analytical HPLC (final concentration: 50 µM).

### The cyclic regulation of ROS generation and scavenging

DCFH was prepared by hydrolyzing DCFH-DA with 0.01 M NaOH at 37 °C for 30 min in the dark. Subsequently, DCFH was diluted to 10 μM in M9 medium. The peptide conjugates were first fully dissolved in methanol and subsequently diluted with AB buffer to 400 µM (2% methanol). For ROS detection, peptide conjugate solution (400 µM) and 10 μM DCFH were mixed in a 1:1 (v/v) ratio, yielding a final peptide concentration of 200 µM in a total volume of 100 μl per well in 96-well plate. At *t* = 0 min and 70 min, samples were irradiated with visible light (12.5 mW cm^−2^) for 10 min. For enzymatic reduction, a mixture of MsrA and MsrB was added at *t* = 0 min and *t* = 80 min to reach a final concentration of 0.5 mg ml^−1^ for each enzyme. Fluorescence intensity of DCF (λ_ex_ = 490 ± 10 nm, λ_em_ = 525 ± 20 nm) was monitored continuously from *t* = 0 to 140 min.

### DLS

Single-angle DLS measurements were performed at 25 °C using a Malvern ZetaSizer Nano S purchased from Malvern Instruments with a He/Ne laser (λ = 633 nm) at a fixed scattering angle of 173°. All measurements were performed in triplicates. The obtained data were processed by CONTIN fitting for intensity-weighted particle size distribution. Samples were prepared at 200 μM in AB buffer.

### TEM

TEM images of the sample solutions were taken on a JEOL 1400 TEM instrument at a voltage of 120 kV. All samples of nanoassemblies were prepared in AB buffer at 200 µM and incubated overnight at 37 °C to allow nanostructure formation. Then, 4 μl of the sample droplets were prepared on Formvar/carbon-film-coated copper grids (300-mesh) by Plano. After 10 min, the solution was removed using filter paper and grids were stained with 7 μl of 4% uranyl acetate for 2.5 min. The grids were washed three times with MilliQ water and dried before measuring. TEM images were processed in ImageJ.

### Plasmid construction

All plasmids were constructed using either restriction enzyme cloning or Gibson assembly. Genetic parts and plasmids used in each experiment are detailed in Supplementary Table 2 and 3. Assembly products were transformed into chemically competent *E*. *coli* DH5α and sequences were confirmed using Sanger sequencing. Following sequencing, the verified plasmids were transformed into chemically competent *E*. *coli* strains, such as MG1655 or BL21.

### Bacterial culture

Typically, glycerol stock of bacteria (kept at −80 °C) was freshly streaked on lysogeny broth (LB)–agar plates once a month. A single colony from the agar plates was cultured overnight in LB medium containing appropriate antibiotics under 37 °C and 160 rpm. The next morning, the overnight culture was subsequently diluted 100:1 in fresh LB medium supplemented with the same antibiotics and incubated for 2–3 h until reaching an optical density at 600 nm (OD_600_ = 0.4–0.5). Following this, the bacterial culture was pelleted by centrifugation at 1,000*g* for 5 min at room temperature and then resuspended in the medium designated for further experiments.

### Expression and purification of MsrA and MsrB

The recombinant plasmid pET28a-msrA or pET28a-msrB was transformed into *E*. *coli* BL21 (DE3) for the heterologous expression of the corresponding enzyme, respectively. A single colony from agar plate was cultured overnight in 10 ml of LB medium containing 50 μg ml^−1^ kanamycin at 37 °C and 160 rpm. The overnight culture was then diluted 1:100 into 1 L of fresh LB medium supplemented with 50 μg ml^−1^ kanamycin and cultured at 37 °C, 160 rpm until OD_600_ = 0.4–0.5. Protein expression was induced by adding 0.5 mM IPTG, followed by incubation at 16 °C overnight. The cells from the overnight culture were harvested by centrifugation (6,300*g*, 12 min, 4 °C). The cell pellet was then resuspended in buffer A (supplemented with 1 mM DTT and 1 mM PMSF) for further purification or in KPi buffer if the CLs were needed for experiments. The resuspended bacteria were lysed by ultrasonication on ice (four 5-min sessions with 30% amplitude, 2.0 s pulse on, 2.0 s pulse off, interspersed with 1-min pauses). Finally, the CL was centrifuged (20,000*g*, 40 min, 4 °C) and the supernatant was collected. The resulting CL supernatant was then filtered through 0.45-μm and 0.20-μm syringe filters and either directly lyophilized for further experiments (that is, denoted as CL) or stored on ice for protein purification. The His-tag-containing protein was purified using a column filled with 3 ml of PureCube His affinity agarose. The column was equilibrated with five column volumes of buffer A before the CLs were loaded at a flow rate of 2 ml min^−1^. Following this, the column was washed with five column volumes of buffer A and then with 5–10 column volumes of wash buffer. Subsequently, the protein was eluted with elution buffer and the fractions containing the protein were transferred to a Pierce protein concentrator PES (10-kDa molecular weight cutoff, 5–20 ml; Thermo Fisher Scientific) for buffer exchange with buffer A (supplemented with 1 mM DTT). The purified protein solution was snap-frozen in liquid nitrogen and lyophilized for further enzymatic experiments. Successful expression of MsrA or MsrB was verified by SDS–PAGE analysis (Supplementary Fig. 13).

### Immunoblot

A single colony cotransformed with the *oxyR-msrA* and *oxyR-msrB* plasmids was inoculated into 10 ml of M9 medium containing 50 µg ml^−1^ kanamycin and 100 µg ml^−1^ ampicillin, followed by overnight incubation at 37 °C and 160 rpm. Next, 1 ml of overnight culture was added to 100 ml of fresh M9 medium containing 50 µg ml^−1^ kanamycin, which was further cultured at 37 °C, 160 rpm until OD_600_ = 0.5; this point was defined as *t* = 0 min. Bacterial cultures were ready for further experiments.

Oxidative stress responses were firstly studied by the H_2_O_2_-induced expression of MsrA and MsrB by adding 10 µM H_2_O_2_ at *t* = 0 and 70 min. Following this, the OD_600_ value of bacteria was measured at predetermined time points. Next, 5 U of OD_600_ bacteria cells were harvested by centrifugation (1,000*g*, 10 min, 4 °C). After removing the supernatant, the resulting pellet was resuspended in 200 µl of B-PER reagent (supplemented with 1 mM DTT and 1 mM PMSF). After that, the cell pellet was resuspended and subjected to three freeze–thaw cycles (liquid nitrogen and 37 °C). Next, 0.2 mg ml^−1^ lysozyme and 50 U per ml Benzonase were added, followed by shaking at 37 °C for 10 min. Any insoluble material was pelleted by centrifugation (13,000*g*, 10 min, 4 °C) and then 100 μl of supernatant CL was transferred to a clean microcentrifuge tube.

To determine the cellular responses to photo-oxidation induced by either **1**_**NF**_ or **2**_**NP**_, we preincubated 400 μM of compound **1** or compound **2** in AB buffer overnight to ensure self-assembly. On the next morning, 0.1 ml of overnight bacterial culture was added in 10 ml of fresh M9 medium containing 50 µg ml^−1^ kanamycin and 100 µg ml^−1^ ampicillin, which was further cultured at 37 °C, with shaking at 160 rpm until OD_600_ = 0.5. Subsequently, 5 ml of bacteria were mixed with 5 ml of **1**_**NF**_ or **2**_**NP**_ in a clean microcentrifuge tube and incubated at 37 °C,160 rpm; this point was defined as *t* = 0 min. At *t* = 0 min and *t* = 70 min, samples were irradiated with visible light (12.5 mW cm^−2^) for 10 min. Then, 1,000-μl mixtures were collected from the microcentrifuge tubes at predetermined time points. Bacterial cells were harvested by carefully removing the supernatant by centrifugation (1,000*g*, 10 min, 4 °C) and the resulting pellet was resuspended with 50 µl of B-PER reagent (supplemented with 1 mM DTT and 1 mM PMSF). The solution was vortexed to resuspend the cell pellet; then the cells were frozen in liquid nitrogen and thawed at 37 °C. These steps were repeated for three cycles. Next, 0.2 mg ml^−1^ lysozyme and 50 U per ml Benzonase were added, followed by shaking at 37 °C for 10 min. Any insoluble material was pelleted by centrifugation (13,000*g*, 10 min, 4 °C) and then 25 µl of the supernatant CL was transferred to a clean microcentrifuge tube.

Before starting blotting, the total protein concentration of the CL was extrapolated by BCA assay and was adjusted to the same levels across all groups. Protein samples were mixed with 5× protein loading buffer to a final concentration of 1× and then boiled at 95 °C for 10 min, resolved on 12% Bis–Tris SDS–PAGE gels and analyzed using a standard immunoblotting protocol. Briefly, the proteins were blotted onto an activated PVDF membrane (0.2 μm) in transfer buffer at 250 mA for 90 min and then blocked with 5% milk in Tris-buffered saline with Tween-20 (TBS-T). PVDF membranes after blocking were incubated with primary 6×His tag monoclonal antibody (1:1,000 dilution) and GAPDH loading control monoclonal antibody (1:2,500 dilution) at 4 °C overnight. Membranes were washed thoroughly with TBS-T and incubated with a secondary anti-mouse IgG horseradish-peroxidase-linked antibody (1:5,000 dilution) at 4 °C for 2 h. After thoroughly washing with TBS-T, the membranes were developed by Pierce ECL western blotting substrate. Western blots were imaged on a chemiluminescence imaging system.

### CLSM-FLIM

CLSM-FLIM imaging was performed on a STELLARIS 8 Leica DMi8 microscope (Leica Microsystems). Briefly, 2 µl of bacterial coculture solution was added to an IBIDI eight-well glass-bottom plate and a 1% low-melt agarose pad is placed on it. Bacterial cells were imaged live using microscope (×63 glycerol-immersion objective) with a fast lifetime contrast (FALCON) module. Samples were excited using a 40-MHz pulse tuned to 488 nm for both intensity and fluorescence lifetime measurements. Emitted photons were detected using GaAsP hybrid photocathode (HyD X) detector with a filter window at 600–700 nm.

Confocal images were taken on a Leica TCS SP5 and Visitron spinning disk microscope. To monitor the GFP, a 488-nm excitation diode laser was used with an emission filter from 510 to 565 nm. For coassembly compounds, an argon laser was used for excitation at 552 nm with an emission filter from 590 to 800 nm.

FLIM was conducted with a cell-free system with a scanning resolution of 1,024 × 1,024 pixels at 100 Hz and four-line accumulations equipped with adaptive focusing on each frame and well position. For *E*. *coli* (*oxyR- msrA* + *oxyR-msrB*) were counted with a scanning resolution of 1,024 × 1,024 pixels at 100 Hz using four-line and five-frame accumulations equipped with adaptive focusing on each frame and well position. Both measurements were based on the FALCON-modified time-correlated single-photon counting method. Each pixel was transformed into a phasor plot according to the following equations:

1 $${g}_{i,j}\left(\omega \right)=\underset{0}{\overset{T}{\int }}I\left(t\right)\bullet \cos \left(n\omega t\right){\rm{d}}t/\underset{0}{\overset{T}{\int }}I\left(t\right){\rm{d}}t$$

2 $${s}_{i,j}(\omega )=\underset{0}{\overset{T}{\int }}I(t)\bullet \sin (n\omega t){\rm{d}}t/\underset{0}{\overset{T}{\int }}I(t){\rm{d}}t$$

in which *g*_*i*,*j*_(*ω*) and *s*_*i*,*j*_(*ω*) are the *x* and *y* coordinates of the phasor plot, *n* and *ω* are the harmonic frequency and the angular frequency of excitation, respectively, and *T* is the repeat frequency of the acquisition. Frequency-domain data acquisition from each pixel can be converted to phasor points using the following transformations:

3 $${g}_{i,j}\left(\omega \right)={m}_{i,j}\bullet \cos \left({\phi }_{i,j}\right)$$

4 $${s}_{i,j}\left(\omega \right)={m}_{i,j}\bullet \sin \left({\phi }_{i,j}\right)$$

in which *m*_*i*,*j*_ and *ϕ*_*i*,*j*_ are the modulation and phase shift, respectively, of the frequency-domain measurement at pixel *i*,*j*. The decay from each pixel can, hence, be translated to a point in the phasor plot. Phasor components were identified and separated using LAS X software. Photons that lie beyond the defined phasor are excluded from the images.

### Detection of nanoassembly-induced intracellular oxidative stress

Overnight bacterial culture and NF preparation were carried out as described above. On the following morning, a fresh bacterial culture (OD_600_ = 0.5 in M9 medium) was prepared. Bacteria were stained with 10 μM DCFH-DA for 1 h at 37 °C. After incubation, to remove excess DCFH-DA, the stained bacteria were centrifuged at 1,000*g* for 5 min and resuspended in fresh M9 medium to OD_600_ = 0.5. Next, 100 μl of stained bacteria were mixed with 100 μl of NFs or AB buffer in a 96-well plate and incubated at 37 °C with shaking at 160 rpm throughout the experiment. This point was defined as *t* = 0 min. Samples were irradiated with visible light (12.5 mW cm^−2^) for 10 min at *t* = 0 min and 70 min. The fluorescence intensity of DCF was measured using a Tecan microplate reader at predetermined time points (λ_ex_ = 490 ± 10 nm, λ_em_ = 525 ± 20 nm).

### Measurement of glutathione redox status in bacteria

The GSH/GSSG ratio and the individual concentrations of GSH and GSSG were measured using a GSH/GSSG-Glo assay following the manufacturer’s instructions. Overnight bacterial culture and NF preparation were carried out as described above. On the following morning, a fresh bacterial culture (OD_600_ = 0.5 in M9 medium) was prepared as described above.

At *t* = 0 min, 1 ml of bacterial culture (*oxyR-msrA*, *oxyR-msrB* or no plasmid) was mixed with 1 ml of NFs and incubated at 37 °C, 160 rpm. Photo-oxidation was induced by 10-min irradiation at 12.5 mW cm^−2^ at *t* = 0 and *t* = 70 min. At predesigned time points, 25-μl aliquots were collected, subjected to three freeze–thaw cycles (liquid nitrogen and 37 °C) and transferred to a 96-well plate (25 μl per well). Subsequently, 25 μl of either the total glutathione lysis reagent or the oxidized glutathione lysis reagent was added to each well and the plate was shaken on an orbital shaker for 5 min. Afterward, 50 μl of luciferin generation reagent was added to all wells and incubated at room temperature for 30 min. Finally, 100 μl of luciferin detection reagent was added, the plate was briefly shaken and incubated for 15 min and the luminescence was measured. The GSH/GSSG ratio was calculated as follows:

$$\begin{array}{l}\displaystyle\frac{{\rm{GSH}}}{{\rm{GSSG}}}{\rm{ratio}}\\\,=\displaystyle\frac{\left({\rm{Net\; total\; glutathione\; luminescence}}-{\rm{Net\; GSSG\; luminescence}}\right)}{\left({\rm{Net\; GSSG\; luminescence}}/2\right)}\end{array}$$

where net luminescence represents the luminescence value of each group after subtracting the average luminescence of the no-GSH control group.

### Measurement of ATP levels in bacteria

The BacTiter-Glo microbial cell viability assay was used to measure intracellular ATP present, following the manufacturer’s instructions. Overnight bacterial culture and NF preparation were carried out as described above. On the following morning, a fresh bacterial culture (OD_600_ = 0.5 in M9 medium) was prepared as previously described.

Subsequently, 5 ml of bacterial cultures (*oxyR-msrA*, *oxyR-msrB* or no plasmid) at an OD_600_ of 0.5 were mixed with 5 ml of NFs in a clean microcentrifuge tube and incubated at 37 °C, 160 rpm throughout the experiment. This point was defined as *t* = 0 min. Samples were irradiated with visible light (12.5 mW cm^−2^) for 10 min at *t* = 0 min and 70 min. At predesigned time points, 100 μl of bacteria–NF mixture was collected and transferred to a 96-well plate. Subsequently, 100 μl of BacTiter-Glo reagent was added to each well and the plate was shaken on an orbital shaker for 5 min before luminescence was measured. The ATP standard curve was generated by replacing the 100-μl bacteria–NF mixture with 100 μl of tenfold serial dilutions of ATP in M9 medium (from 1 μM to 10 pM), following the same assay procedure as used for the bacteria–NF samples.

### ATP synthesis inhibitor assay

To discern whether the uptake mechanism of **2**_**NP**_ is by passive diffusion or active transport, an ATP synthase inhibitor (DCCD) was used. A 0.1 mM stock solution of DCCD was prepared in 100% dimethyl sulfoxide and added to the designated experimental groups at a 1:10 dilution ratio, yielding a final concentration of 10 μM DCCD. This treatment allowed us to assess the contribution of ATP-dependent processes the influx–efflux cycles.

### Flow cytometric analysis of bacterial uptake qualification and quantification

Flow cytometric analysis was performed using a BD LSRFortessa SORP flow cytometer. Sheath fluid consisted of BD FACSFlow and the sample flow rate was constant throughout the analysis of all bacterial mixtures. Detectors used were side scatter with the voltage set at 320 V and forward scatter, while the fluorescence detector settings varied between fluorophores. Hoechst 33342 fluorescence (λ_ex_ = 355 nm) was detected using the 450/50-nm bandpass filter. GFP fluorescence (λ_ex_ = 488 nm) was detected using the 530/30-nm bandpass filter. PPIX fluorescence (λ_ex_ = 405 nm) was detected using the 610/20-nm bandpass filter. All parameters were collected as logarithmic signals. A total of 20,000 fluorescence events from each bacterial mixture were collected for subsequent analysis. Proper gates for the detection of GFP^+^PPIX^+^ and PPIX^+^Hoechst^+^ fluorescence events were set with blank and control solutions containing equivalent amounts of unlabeled *E*. *coli* (GFP^−^), *E*. *coli* (Hoechst^−^), *E*. *coli* (GFP^+^) and *E*. *coli* (Hoechst^+^). These unlabeled cells were simultaneously prepared with the labeled cells and analyzed before the fluorescence bacterial assays. No peptide conjugates were added to the blank solution; only equal volumes of the appropriate buffer solution were added. Bacteria in different groups were collected and diluted 20-fold in MilliQ water at different time intervals. For Hoechst 33342 staining, living *E*. *coli* (cotransformed with *oxyR-msrA* + *oxyR-msrB*) cells were stained with Hoechst 33342 for 10 min at room temperature. Data analyses were carried out with BD FACSDiva version 9.7 and FlowJo version 10.8.1.

### Reporting summary

Further information on research design is available in the Nature Portfolio Reporting Summary linked to this article.

## Online content

Any methods, additional references, Nature Portfolio reporting summaries, source data, extended data, supplementary information, acknowledgements, peer review information; details of author contributions and competing interests; and statements of data and code availability are available at https://doi.org/10.1038/s41589-026-02279-x.

## Supplementary information

Supplementary Information Supplementary Methods, Discussion, Figs. 1–40 and Tables 1 and 2.

Reporting Summary

Supplementary Table 3 DNA sequences of genetic parts and plasmids used in this study.

## Source data

Source Data Fig. 2 Statistical source data.

Source Data Fig. 3 Statistical source data.

Source Data Fig. 4 Unprocessed western blots.

Source Data Fig. 4 Statistical source data.

Source Data Fig. 5 Statistical source data.

## Data Availability

Plasmids described in this study were deposited to the Addgene repository under accession numbers 257812–257815. The data that support the findings of the present study are available within the article and its Supplementary Information. All data have been uploaded to a Zenodo open-access repository (https://doi.org/10.5281/zenodo.15304145)^49^. Source data are provided with this paper.
