# Supplementary material for: Restoring intracellular homeostasis disrupted by synthetic nanoassemblies
Source: Nat Chem Biol. 2026 Jul 17;22(10):1676–84. doi: 10.1038/s41589-026-02279-x (PMC13612220; doi:10.1038/s41589-026-02279-x)

nature chemical biology

Supplementary information

https://doi.org/10.1038/s41589-026-02279-x

Restoring intracellular homeostasis disrupted by synthetic nanoassemblies

In the format provided by the authors and unedited

Table of Contents

Contents

1. Materials **3**

2. Supplementary methods **3**

2.1 Preparative HPLC 3

2.2 Analytical HPLC 3

2.3 HPLC-MS 3

2.4 Matrix-assisted laser desorption/ionization-time of flight mass spectrometry (MALDI-TOF-MS) 4

2.5 Circular dichroism spectroscopy 4

2.6 UV-vis absorption, fluorescence spectroscopy, and critical aggregation concentration (CAC) measurements 4

2.7 Fourier transform infrared (FT-IR) spectroscopy 4

2.8 Light source for irradiation 4

2.9 Bacterial viability assays 5

2.10 Bioluminescence measurements 5

2.11 Membrane integrity assay 6

3. Synthesis and structural characterization **7**

3.1 Compound 1 7

3.2 Compound 2 8

4. Characterization of intermediate, mono-oxidized compound 3. .... **10**

5. H_2O_2-induced oxidation of control peptide, DVMVMD. **10**

6. Comparison between synthesized compound 2 (2_{syn}) and directly after irradiation from compound 1 (2_{irr}) **11**

6.1. Stereochemistry of 2_{syn}. 11

6.2. Optical properties of 2_{syn} and 2_{irr}. 12

7. Enzyme activity. **12**

8. Multiple redox cycles at the molecular level **13**

9. Quantitative analysis of reactive oxygen species (ROS) generation **14**

10. Critical aggregation concentrations (CAC) of compound 1 and compound 2. .... **14**

11. Optical properties of 1 and 2 .... **15**

12. The hydrodynamic diameter of 2_{NP} **15**

13. Secondary structures **16**

14. Phasor- FLIM to monitor assembly progression and assemblies' transformation..17

15. Bacterial viability...19

16. Time-dependent and morphological-induced bacterial internalization 20

16.1 Gating strategies for *E. coli* GFP 20

16.2 Time-dependent bacterial internalization and quantification. 20

17. Temperature-dependent nanoparticle internalization. 23

18. Energy-dependent nanoparticle internalization. 24

19. Membrane integrity. 24

20. Bacterial responses to oxidative stress 25

21. Bacterial viability across cycle-relevant time scales. 26

22. Phasor-FLIM for analyzing bacteria and material interactions 26

23. Flow cytometry indicates a homeostatic synthetic biosystem 28

24. Bacterial lysis analysis 28

Supplementary Table 1. Buffer compositions used in this study..........................30

Supplementary Table 2. Plasmids used in this study. 31

Supplementary Table 3. DNA sequences of genetic parts and plasmids used in this study. (See separated file)..31

Supplementary References...31

1. Materials

Fmoc-protected amino acids, Fmoc-protected methionine sulfoxide, and Wang resin were purchased from Novabiochem®. OxymaPure®, N-ethyldiisopropylamine, piperidine, trifluoroacetic acid, ampicillin, kanamycin, Lysogeny broth (LB) medium, LB-agar, powdered milk, and transfer membrane (polyvinylidene fluoride, PVDF, 0.2 μm) were obtained from Carl Roth. Dimethylformamide (DMF for peptide synthesis), diethyl ether, and dimethylsulfoxide were purchased from Acros Organics. Acetonitrile, sulforhodamine 101, singlet oxygen sensor green, dithiothreitol (DTT), Hoechst 33342 (NucBlueTM), B-PER™ reagent for bacterial protein extraction, Pierce™ BCA protein assay kits, and Pierce™ ECL western blotting-substrate were purchased from Thermo Fisher Scientific. Oxalyl chloride (>98.0%) was purchased from TCI. Uranyl acetate and fluorescamine were purchased from Merck. Dulbecco's phosphate-buffered saline (DPBS), thioflavin T (ThT), Glucose, isopropyl β-D-1-thiogalactopyranoside (IPTG), phenylmethylsulfonyl fluoride (PMSF), imidazole, nickel (II) chloride, calcium (II) chloride, magnesium (II) chloride, lysozyme from chicken egg white (~100000 U/mg), Benzonase, Propidium iodide, and N, N′-Dicyclohexylcarbodiimide (DCCD) were purchased from Sigma Aldrich. 6x-His Tag Monoclonal Antibody (#MA1-135), GAPDH Loading Control Monoclonal Antibody (#MA5-15738), and 2',7'-dichlorofluorescein diacetate (DCFH-DA) were purchased from Invitrogen. Anti-mouse IgG, HRP-linked Antibody (#7076) was purchased from Cell Signaling. PureCube Ni-NTA agarose beads (31103) were purchased from Cube Biotech. GSH/GSSG-glo Assay, and BacTiter-Glo Microbial Cell Viability Assay were purchased from Promega. The compositions of buffers used in this study are listed in Supplementary Table S1. Reagents and solvents were purchased from commercial sources and were used without further purification. Peptide Synthesis grade reagents were used for synthesis of the peptides.

2. Supplementary methods

2.1 Preparative HPLC

The compounds were purified by preparative HPLC using a setup by Shimadzu. For purification, a Phenomenex Gemini 5 μM NX-C18 110 Å 150 × 30 mm was used at a flow rate of 25 mL/min. A gradient of CH3CN in HPLC grade (containing 0.1% CF_3COOH) and MilliQ water (containing 0.1% CF_3COOH) was used as eluent. The chromatographic peak fractions are collected at 254 nm.

2.2 Analytical HPLC

The samples were analyzed by analytical HPLC using a setup by Shimadzu. A ZORBAX Eclipse XDB-C18 HPLC column (4.6 × 250 mm, 50 μM) was used at a flow rate of 1 mL/min. Experiments were performed using CH_3CN in HPLC grade (containing 0.1% CF_3COOH) and MilliQ water (containing 0.1% CF_3COOH). The solvent gradient started with 100% MilliQ water, then the CH_3CN content was linearly increased to 100% in 20 min. The molar ratio of the compounds was calculated using peak areas at 254 nm. Data were processed in LabSolutions and Origin2024b.

2.3 HPLC-MS

The compounds were purified by preparative HPLC using a setup by Shimadzu. For purification, a Phenomenex Gemini 5 μM NX-C18 110 Å 150 × 30 mm was used at a flow rate of 25 mL/min. A gradient of CH_3CN in HPLC grade (containing 0.1%

CF_3COOH) and MilliQ water (containing 0.1% CF_3COOH) was used as eluent. The chromatographic peak fractions are collected at 254 nm.

2.4 Matrix-assisted laser desorption/ionization-time of flight mass spectrometry (MALDI-TOF-MS)

All MALDI-TOF-MS spectra were recorded on a rapifleX MALDI-TOF/TOF from Bruker. Samples were mixed with a saturated solution of the matrix S7 α-cyano-4-hydroxycinnamic acid (CHCA) in MilliQ water/CH_3CN 1/1 + 0.1% CF_3COOH and prepared via the dried droplet method. Data processing was performed in mMass.

2.5 Circular dichroism spectroscopy

Circular Dichroism (CD) spectra were recorded on a JASCO J-1500 spectrometer in a Hellma high-precision cell with a 1 mm light path. $\mathbf{1_{NF}}$ and $\mathbf{2_{NP}}$ were dissolved in a mixture of 2% methanol and 5 mM NH_4HCO_3 buffer pH=7.5 at 200 µM. Data were processed in Spectra Analysis by JASCO and OriginPro 2019b.

2.6 UV-vis absorption, fluorescence spectroscopy, and critical aggregation concentration (CAC) measurements

To record the absorbance and fluorescence intensity, a SPARK 20M microplate reader by Tecan Group Ltd. was used. The samples (50 µM in AB buffer) were measured in a Greiner 96 flat transparent well plate for absorbance measurement, black well plates for fluorescence measurements (Excitation wavelength = 410 nm.) For the CAC analysis, PS-peptide conjugate solutions were prepared at gradient concentrations in AB buffer and placed in black UV Star ® 384 microliter well-plates (Greiner bio– one). The maximum emission intensity at 426 nm under excitation at 410 nm was quantified. The data was processed in OriginPro2024b and Prism 10.

2.7 Fourier transform infrared (FT-IR) spectroscopy

FT-IR spectroscopy of all reported peptides was performed using a Bruker (Tensor-27) FT-IR spectrophotometer. 200 µL of the respective peptide solutions (200 µM in AB buffer) were lyophilized and the resulting powders were prepared with KBr as pellets. Samples are scanned between 600 cm^{-1} to 4000 cm^{-1} over 64 scans at a resolution of 4 cm^{-1} and an interval of 1 cm^{-1}. The data was processed in OriginPro 2019b.

2.8 Light source for irradiation

Compound **1** (50 µM, final concentration) was added to a glass vial (1.5 mL, 32x11.6 mm) and then irradiated with an LED array light source (cold white LED, 4600 K - 9000 K, 3.0 mW/cm^2), purchased from Thorlabs, Inc., see Supplementary Figure 1). After a predetermined irradiation time interval, photo-oxidation was stopped by removing the light source.

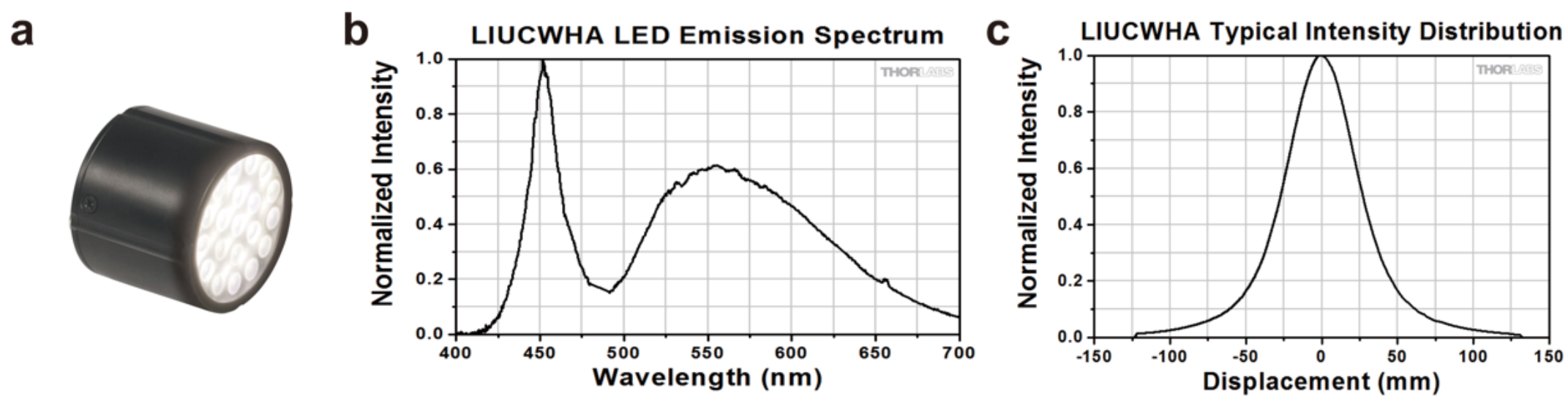

Supplementary Figure 1. a. Cold white LED array employed in this study. b. Normalized emission spectrum of the cold white LED array (middle). c. The intensity distribution of the LED in the plane is located 100 mm from the LED along the emission axis (right). Adapted from Manufacturer's specifications Thorlabs, Inc.

For the bacteria viability test, in a well-plate, the photo-irradiation was conducted using an in-house-built photobox that is equipped with LED arrays (96 LEDs, 25 mA, 10.2 mW/cm^2) of 410 nm, see Supplementary Figure 2.

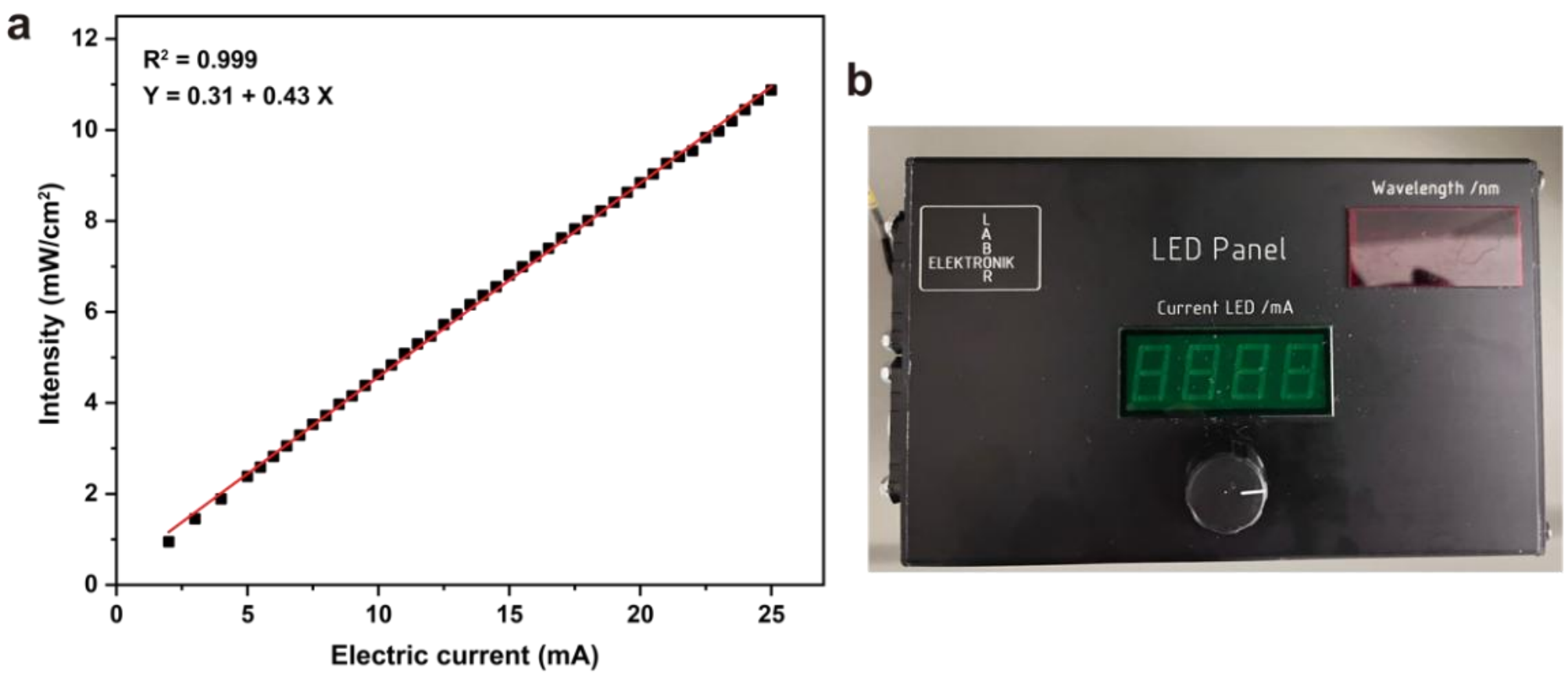

Supplementary Figure 2. a. The in-house-built photo-box has a linear relationship between current and intensity. b. Optical image of the in-house-built photo-box.

2.9 Bacterial viability assays

Bacterial viability assays were performed using *E. coli* GFP and *E. coli* AB. Based on the bacterial culture method described in the main text section, both strains were each diluted to OD_{600} = 0.2 and co-cultured with samples in a 1:1 volume ratio at 37 ^oC and 160 rpm. At various time points,100 µL of culture solution was extracted and diluted in 900 µL of sterile distilled water. Further 4-5-fold dilutions were made, resulting in 1 ml of diluted culture available for plating. Colony-forming units (CFU) were quantified by plating 2 µL of diluted culture on agar plates and incubating overnight. All irradiation experiments used photobox (96 LEDs, 25 mA, 10.2 mW/cm^2) of 410 nm, see Supplementary Figure 2.

2.10 Bioluminescence measurements

The oxyR-lux gene circuit was first verified by the production of bioluminescence. A single colony transformed with the oxyR-lux plasmid was inoculated into 10 mL of LB medium containing 50 µg/mL Kanamycin, followed by overnight incubation at 37 °C and 160 rpm. 400 µL of overnight culture was added in 20 mL of fresh LB medium

containing 50 µg/mL Kanamycin, which was further cultured at 37 °C, 160 rpm until the OD_{600}=0.4-0.5. The bacteria were harvested by centrifugation, and the supernatant was carefully removed. After resuspension in fresh LB medium to achieve an OD_{600} of 0.1, 50 µL of the resulting bacterial suspension for both the illumination and dark groups was mixed with 50 µL of either 400 µM solution of compound **1** or **2**. In the H_2O_2 group, 50 µL of the bacterial suspension was mixed with 50 µL of AB buffer. The mixtures of the illumination group were first transferred to 96-well white plate and exposed to light for 10 minutes at 12.5 $mW·cm^{-2}$. Immediately following the illumination, additional H_2O_2 was added to the H_2O_2 group's mixtures to achieve final H_2O_2 concentrations of 200, 100, 50, 25, 12.5, 6.25, 3.125, and 0 µM. Subsequently, the mixtures of the dark and H_2O_2 groups were then transferred to the 96-well white plate and measurements were started immediately. All plates were incubated aerobically with shaking for 2 hours at 37°C for all groups, and the bioluminescence intensities were measured every 5 minutes between 400-500 nm using a SPARK 20M microplate reader.

2.11 Membrane integrity assay

Overnight bacterial culture and NF preparation were carried out as described above. On the following morning, a fresh bacterial culture with an OD600 of 0.5 in M9 medium was prepared as previously described in Immunoblot section.

Subsequently, 1 mL of bacterial cultures (co-transformed with oxyR-msrA and oxyR-msrB) at an OD600 of 0.5 were mixed with 1 mL of NFs in a clean microcentrifuge tube. Afterwards, NF-bacteria mixture was transferred to a 96-well plate (100 µL per well) and incubated at 37 °C, 160 rpm throughout the experiment. This point was defined as t = 0 min, a 10-minute irradiation at 12.5 mW·cm–2 was applied at t = 0 min and t=70 min for photo-oxidation. At predetermined time points (i.e., t = 0, 10, 40, 70 min), 1 µL of 1 µM propidium iodide (PI) solution in DMSO was added into the well assigned to that time point (three well per time point, with distinct wells assigned to each time point). Following incubation in the dark on an orbital shaker for 5 minutes, CLSM images were acquired using a Leica SP8 confocal microscope with Ex = 553 nm and Em = 620-700 nm. Fluorescence intensity was quantified using a Tecan microplate reader (Ex = 535 ± 10 nm, Em = 620 ± 20 nm).

3. Synthesis and structural characterization

3.1 Compound 1

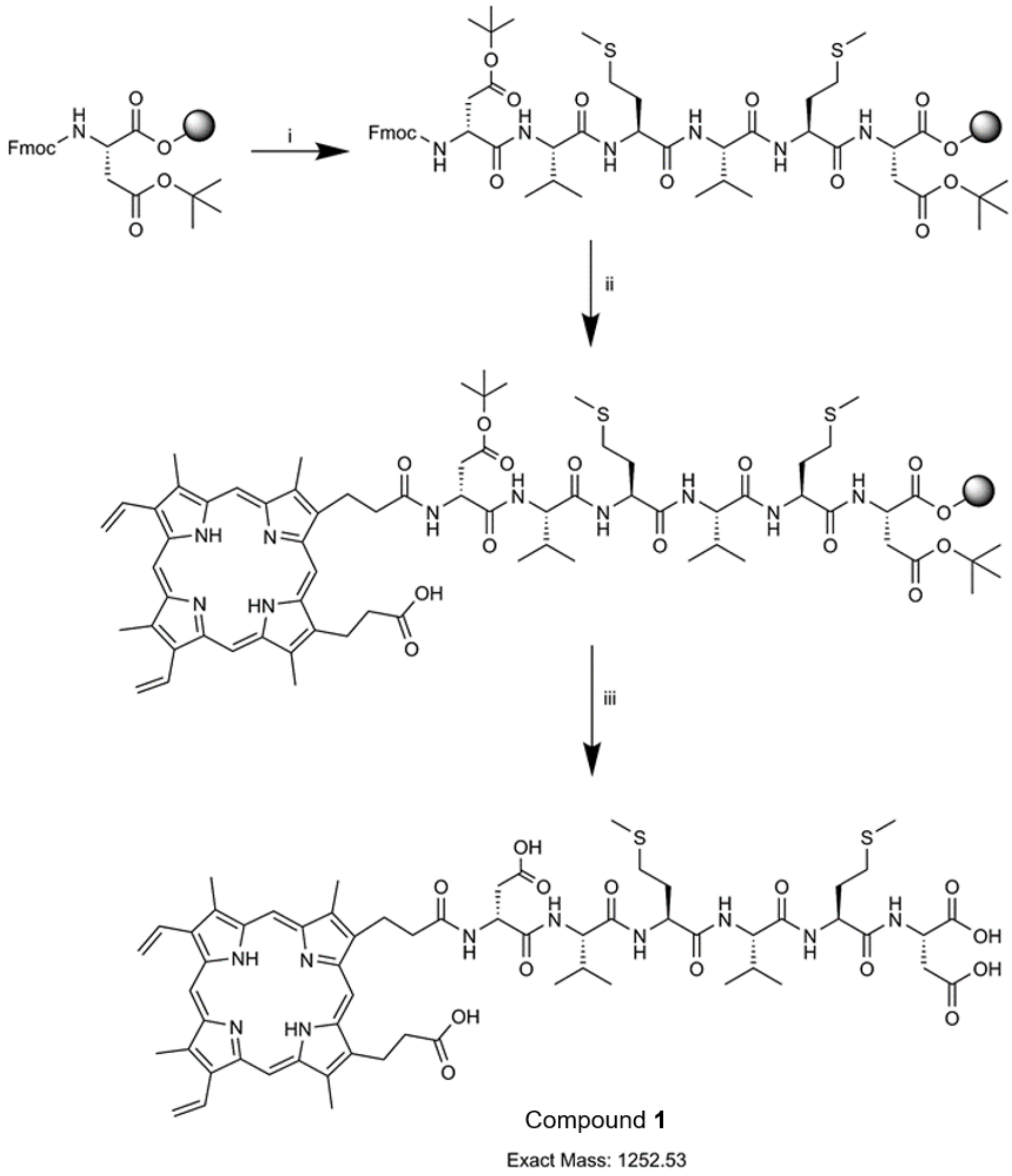

Supplementary Figure 3. Synthesis of compound **1**. (i) Solid phase peptide synthesis (SPPS) was used to prepare Asp (OtBu)-Val-Met-Val-Met-Asp (OtBu)-Resin (ii) PPIX, Oxyma pure, DIC, DMF, RT, 16 h. (iii) 95% TFA, 2.5% TIPS, 2.5% H_2O, RT, 1.5 h. The product was purified by HPLC using the Zorbax Eclipse column at a flow rate of 4 mL/min. The gradient started with 5% CH_3CN in H_2O (+0.1% CF_3COOH) and this solvent ratio was kept for 1 min, after which the CH_3CN content was increased to 100% over 13 min. Compound **1** eluted from the column after 11.2 min and was received as a light brown powder after lyophilization (9.8 % overall yield). (HPLC-MS and MALDI-TOF data are in **Supplementary Figure** 5 and 6)

3.2 Compound 2

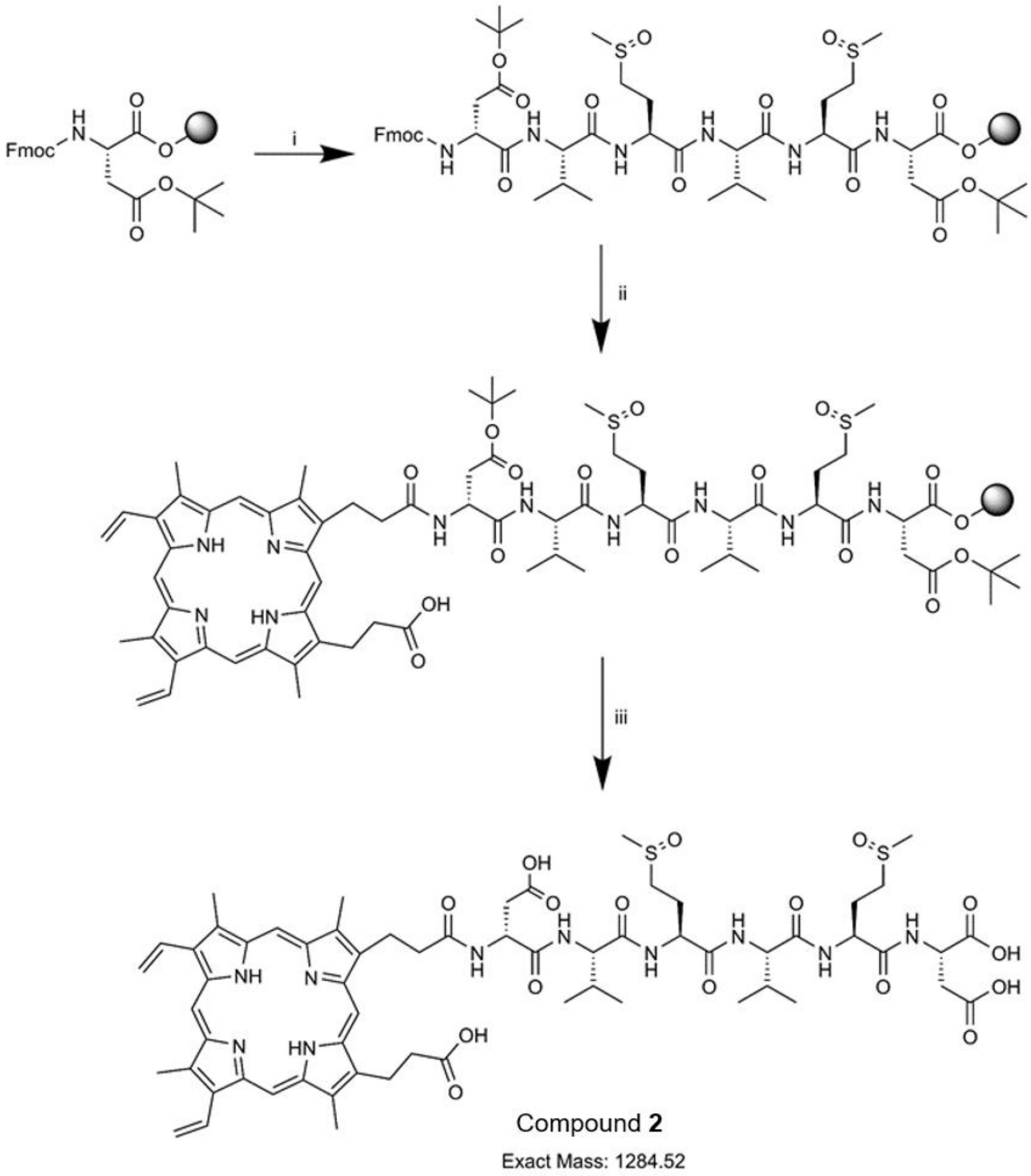

Supplementary Figure 4. Synthesis of compound **2**. (i) Solid phase peptide synthesis (SPPS) was used to prepare Asp (OtBu)-Val-Met(O)-Val-Met(O)-Asp (OtBu)-Resin (ii) PPIX, Oxyma pure, DIC, DMF, RT, 16 h. (iii) 95% TFA, 2.5% TIPS, 2.5% H_2O (HPLC-MS data are in **Supplementary Figure** 1) The product was purified by HPLC using the Zorbax Eclipse column at a flow rate of 4 mL/min. The gradient started with 5% CH_3CN in H_2O (+0.1% CF_3COOH) and this solvent ratio was kept for 1 min, after which the CH_3CN content was increased to 100% over 13 min. Compound **2** eluted from the column after 10.5 min and was received as a light brown powder after lyophilization (8.5 % overall yield). (HPLC-MS and MALDI-TOF data are in **Supplementary Figure** 5 and 6)

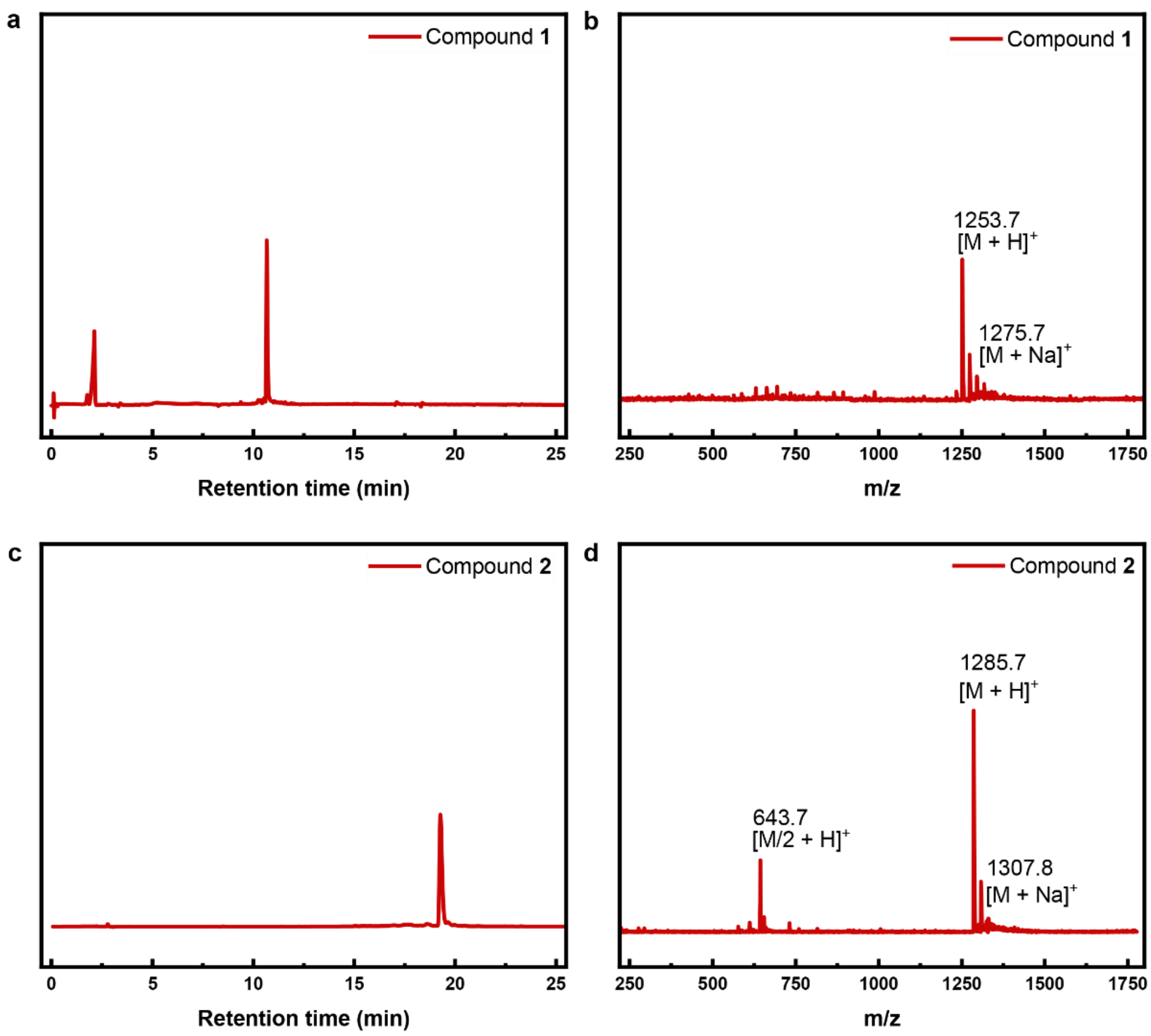

Supplementary Figure 5. HPLC-MS study of compound **1** and compound **2**. a. HPLC trace of compound **1**. Retention time (t_R) = 10.6 min. b. Calculated for $[M]^+$ ($C_{62}H_{80}N_{10}O_{14}S_2$): 1252.53, found mass (m/z): 1253.7 $[M+H]^+$ and 1275.7 $[M+Na]^+$. c. HPLC trace of compound **2**. Retention time (t_R) = 19.2 min. d. Calculated for $[M]^+$ ($C_{62}H_{80}N_{10}O_{16}S_2$): 1284.52, found mass (m/z): 643.7 $[M/2+H]^+$,1285.7 $[M+H]^+$ and 1307.8 $[M+Na]^+$.

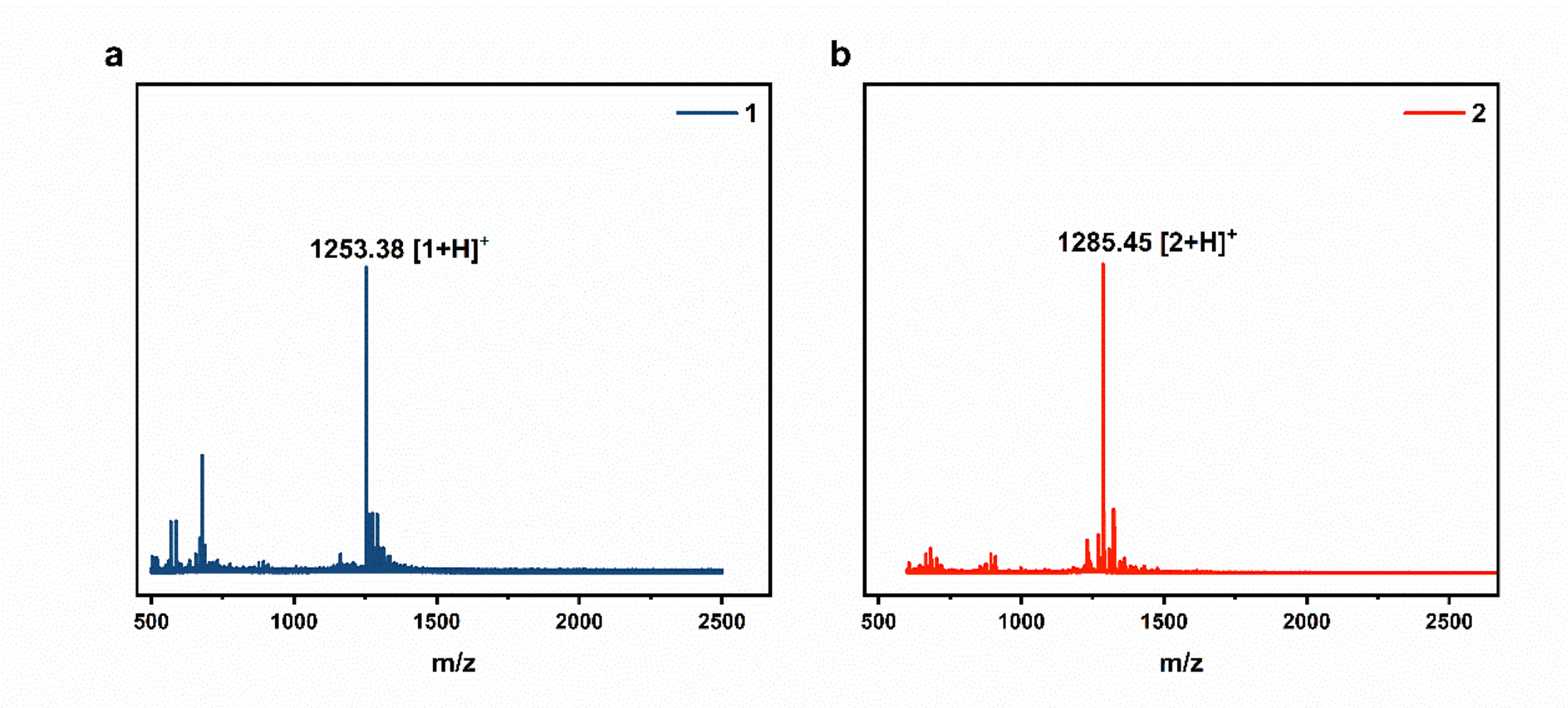

Supplementary Figure 6. MALDI-TOF mass spectrum for compound **1** and compound **2** (LC traces are shown in Fig. 2). a. Calculated for $[M]^+$ ($C_{62}H_{80}N_{10}O_{14}S_2$): 1252.53, found mass (m/z): 1253.38 $[$**1**$+H]^+$. b. Calculated for $[M]^+$ ($C_{62}H_{80}N_{10}O_{16}S_2$): 1284.52, found mass (m/z): 1285.45 $[$**1**$+H]^+$.

4. Characterization of intermediate, mono-oxidized compound 3.

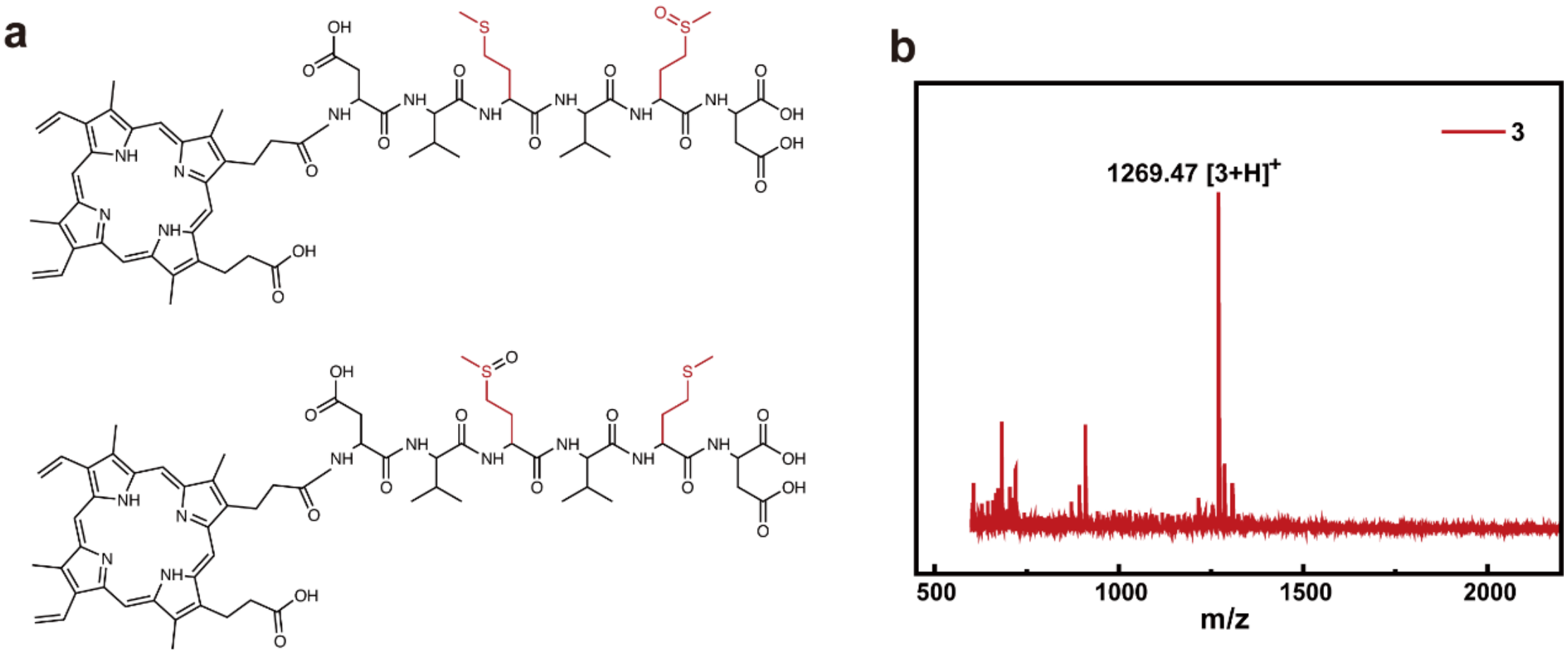

Supplementary Figure 7. a. Structures of the isomers termed compound **3** (mono-oxidized 1) and b. Calculated for $[M]^+$ ($C_{62}H_{80}N_{10}O_{15}S_2$): 1268.52. MALDI-TOF mass spectrum (m/z): 1269.47 $[$**3** $+ H]^+$.

5. H_2O_2-induced oxidation of control peptide, DVMVMD.

A solution of 50 µM control peptide, DVMVMD, was prepared by dissolving it in AB buffer. Subsequently, 20 mM H_2O_2 was added to oxidize methionine in the peptide sequence at 37 ^oC. At each time point, 100 µL of the solution was injected into HPLC-MS.

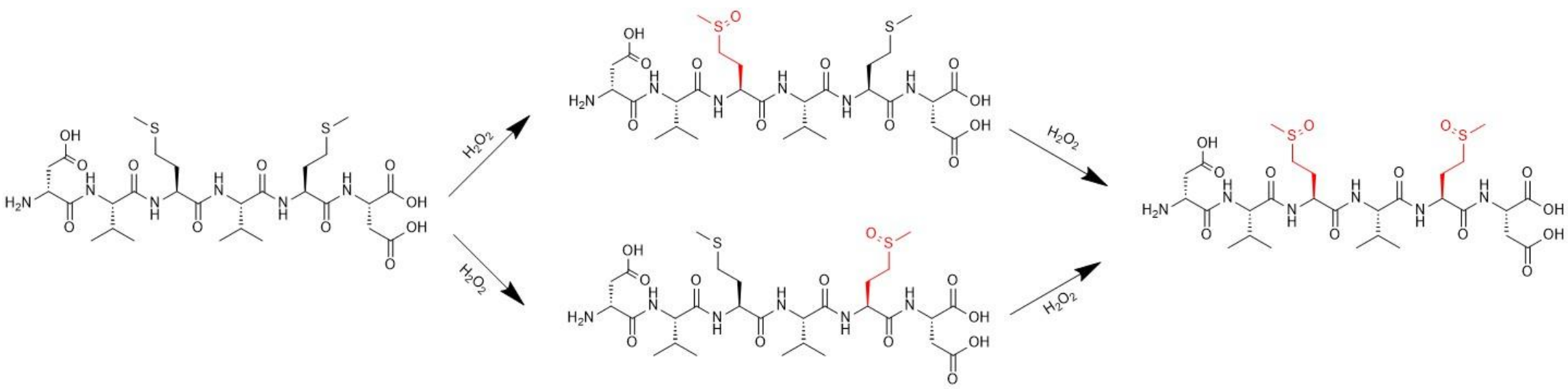

Supplementary Figure 8. Oxidation scheme of control peptide, DVMVMD.

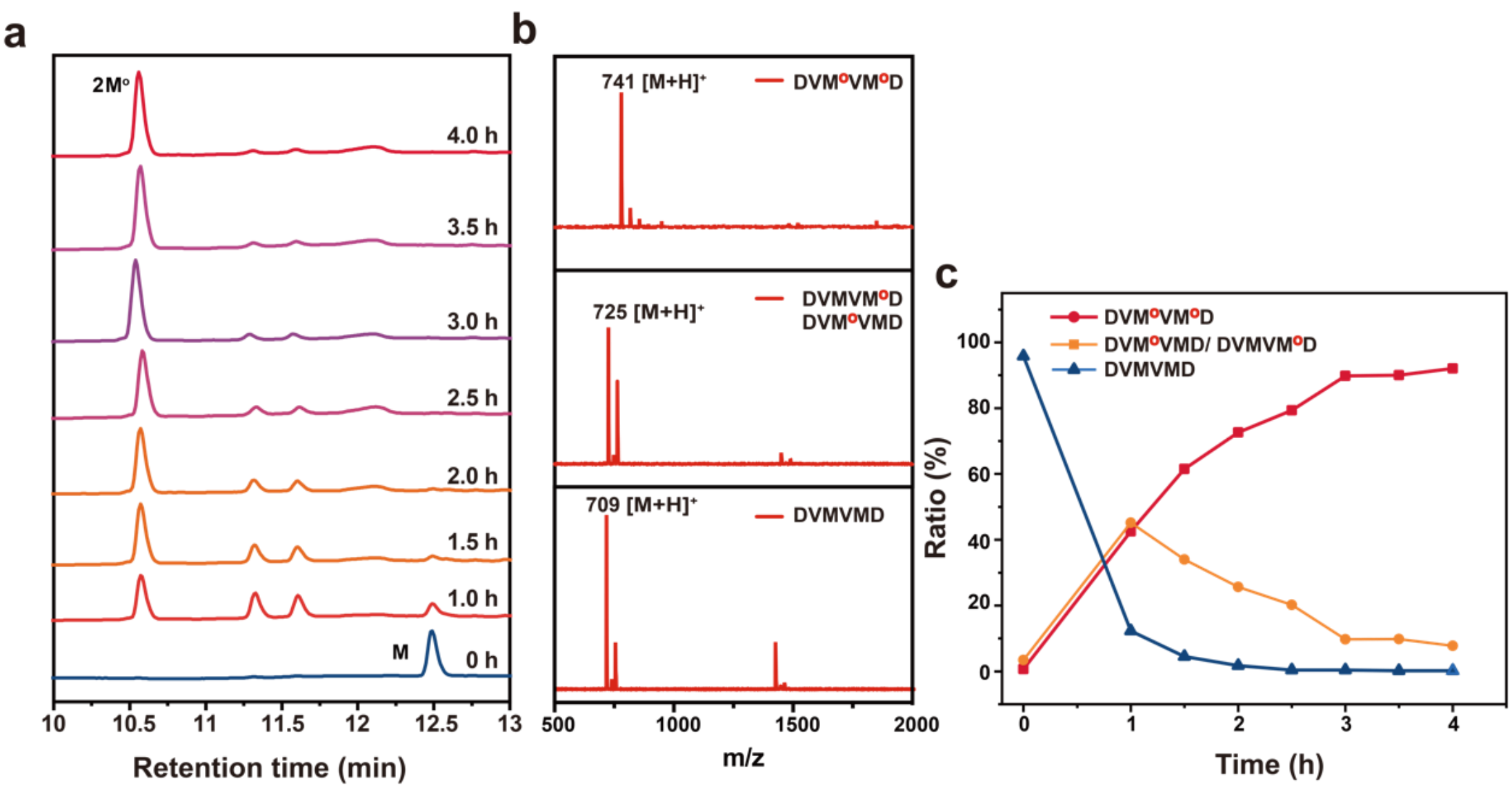

Supplementary Figure 9. a. Kinetic analysis of the H2O2-induced oxidation of control peptide, DVMVMD (50 μM) in AB buffer by using HPLC-MS. b. Convoluted MS spectra in the HPLC-MS analysis for a reaction time of 0 min and t_R = 12.3 min (identified as DVMVMD) and a reaction time of 4 h and t_R = 10.7 min (identified as DVM^{o}VM^{o}D) The other two peaks are identified as intermediates (M_1^o; DVM^{o}VMD and M_2^o; DVMVM^{o}D). c. Molar ratio of DVMVMD, DVM^{o}M^{o}D, and intermediates based on the peak integration at 254 nm.

6. Comparison between synthesized compound 2 (2_{syn}) and directly after irradiation from compound 1 (2_{irr}).

6.1. Stereochemistry of 2_{syn}.

Notably, the light-induced sulfide oxidation is unselective, whereas methionine sulfoxide reductases (Msrs) are stereospecific. It can be demonstrated that the $\mathbf{2_{syn}}$ also has two isomers, given that the enzyme, MsrA, can reduce only one stereoisomer, i.e. approximately 50% of $\mathbf{2_{syn}}$ (Supplementary Figure 10).

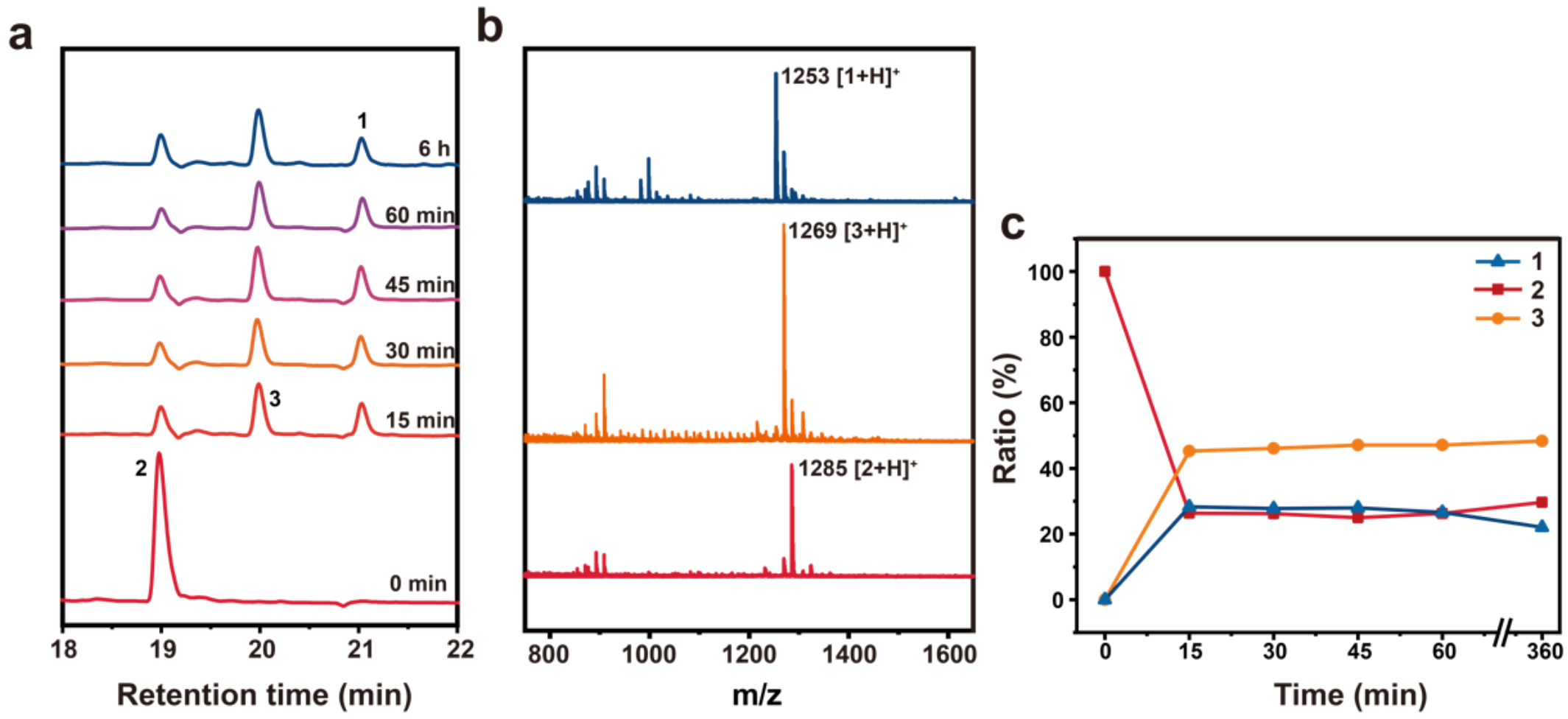

Supplementary Figure 10. a. Kinetic analysis of the reduction of $\mathbf{2_{syn}}$ was conducted using cell lysates (CL) of MsrA (5.6 mg) in AB buffer supplement with 500 μM DTT. Aliquots of 50 μL of the mixture were taken at different time intervals, precipitated with 4 volumes of methanol, and injected into an analytical HPLC. The final concentration is 50 μM. b. Convoluted MS spectra in the HPLC-MS analysis for a reaction time of 0 min and t_R = 19 min

(identified as compound **2**) t_R = 20.3 min (identified as compound **3**) and t_R = 21.2 min (identified as compound **1**). c. Molar ratio of compounds **1**, **2**, and **3** based on the peak integration at 254 nm.

6.2. Optical properties of 2_{syn} and 2_{irr}.

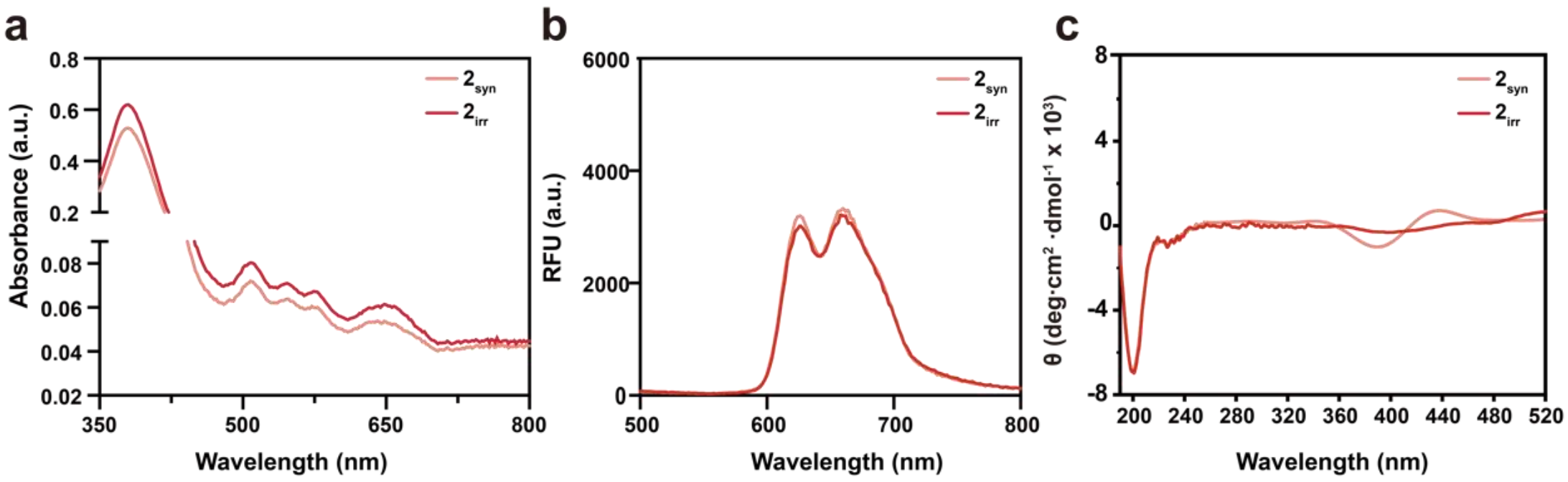

Supplementary Figure 11. a. Absorbance spectra of $\mathbf{2}_{syn}$ and $\mathbf{2}_{irr}$ (50 μM) in AB buffer. b. Fluorescence emission spectra of $\mathbf{2}_{syn}$ and $\mathbf{2}_{irr}$ (50 μM) in AB buffer. Excitation wavelength = 410 nm. c. Circular dichroism (CD) spectra of $\mathbf{2}_{syn}$ and $\mathbf{2}_{irr}$ (200 μM) in AB buffer.

7. Enzyme activity.

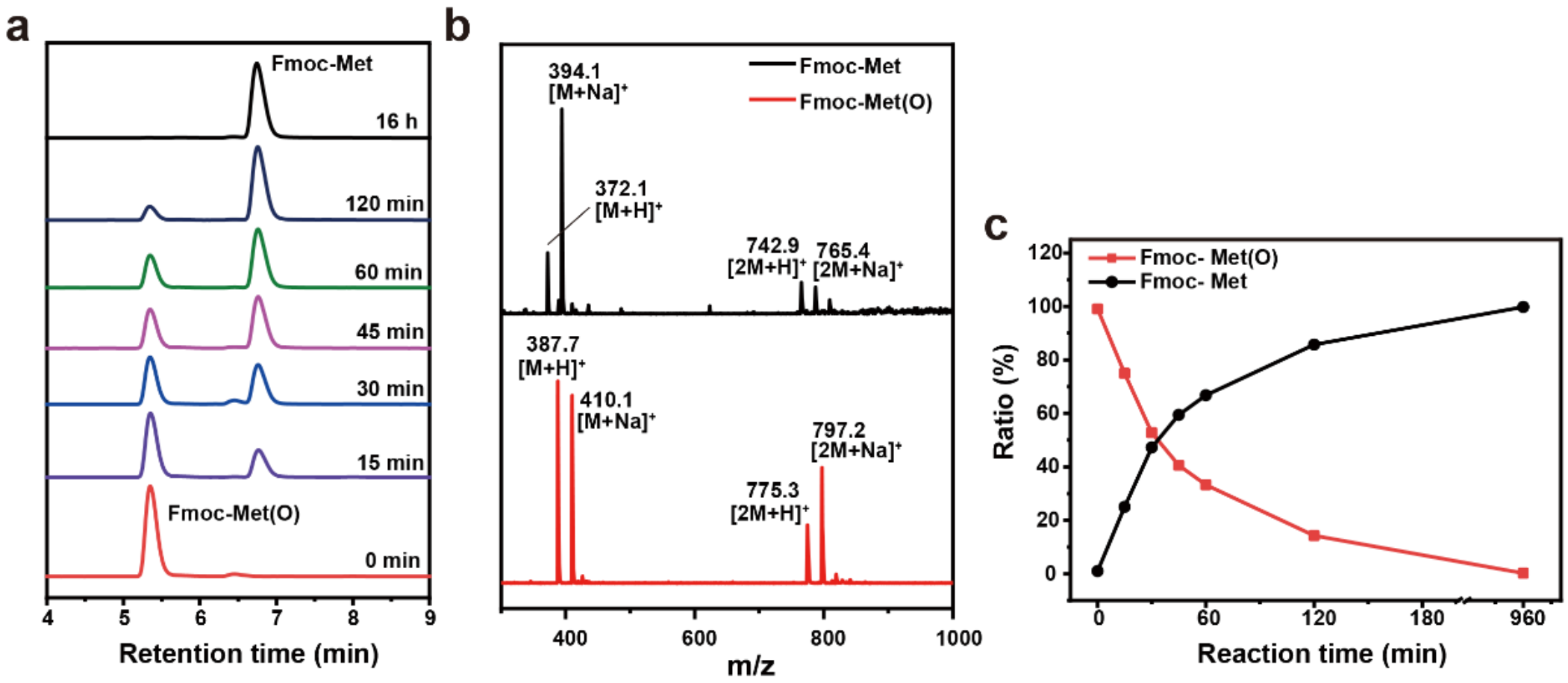

Supplementary Figure 12. a. Kinetic analysis of the enzymatic reduction of Fmoc-Met(O) (200 μM) was performed using 50 μg MsrA and 50 μg MsrB in AB buffer supplement with 500 μM DTT monitored by HPLC-MS. Aliquots of 50 uL were taken at different time intervals, precipitated with 4 volumes of methanol, and the supernatant was injected into an analytical HPLC. The final peptide concentration is 50 μM. b. Convoluted MS spectra in the HPLC-MS analysis for a reaction time of 0 min and t_R = 5.37 min (identified as Fmoc-Met (O)) and a reaction time of 16 h and t_R = 6.8 min (identified as Fmoc-Met). c. Molar ratio of Fmoc-Met and Fmoc-Met (O) based on the peak integration at 254 nm.

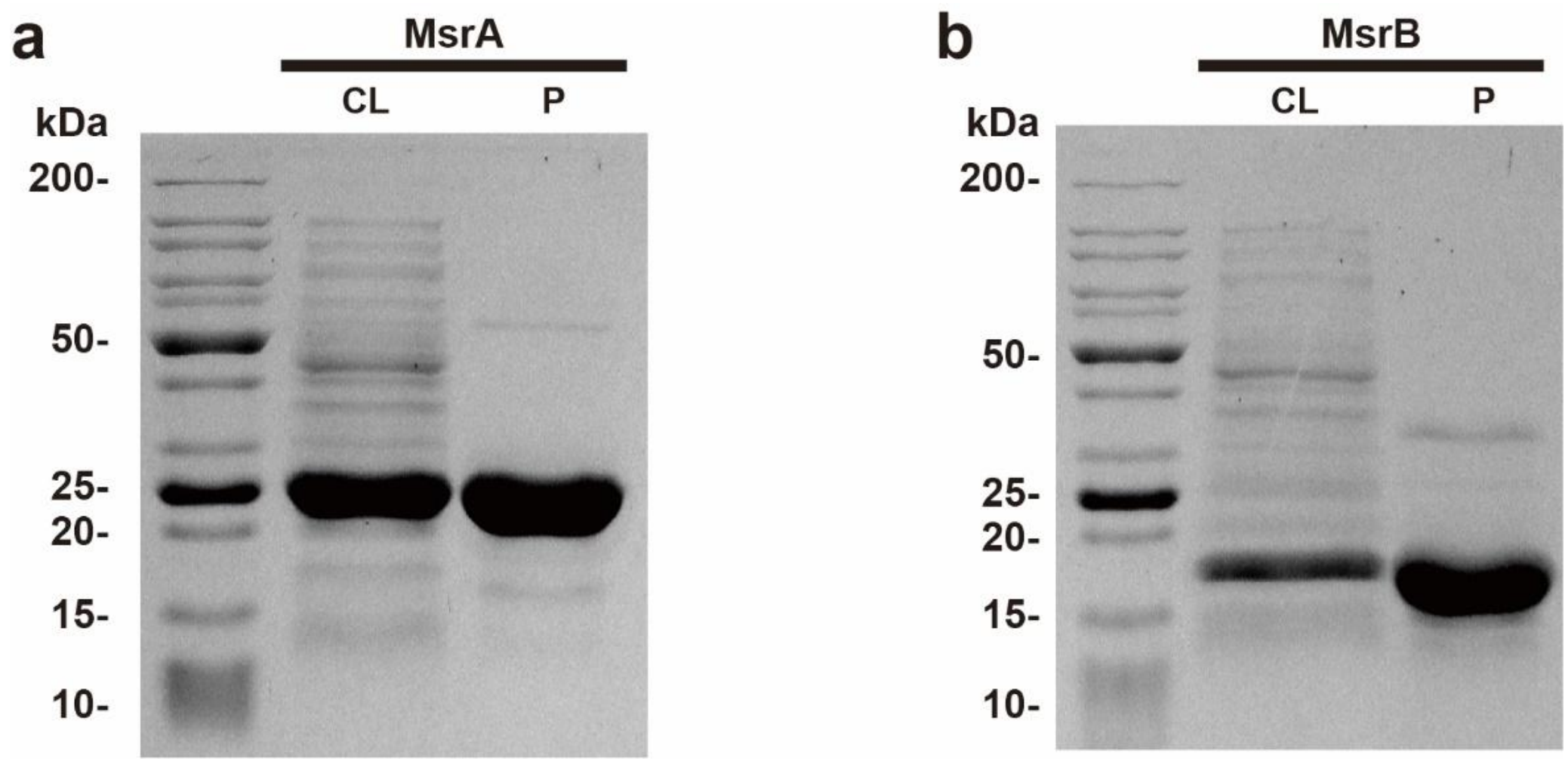

Supplementary Figure 13. SDS-Page to verify the expression of a. MsrA or b. MsrB. CL = cell lysates, P = protein solution. MsrA (26.8 kDa). MsrB (18.9 kDa).

8. Multiple redox cycles at the molecular level

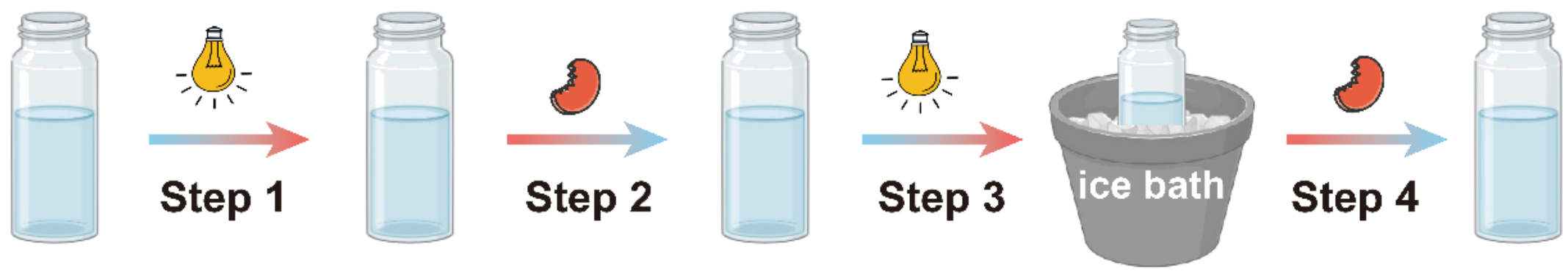

Supplementary Figure 14. Schematic illustration of the molecular cycling experimental design. The molecular cycling is conducted by repeatedly exposing the sample to light irradiation and enzymes (MsrA and MsrB CL) addition. At step 3, to avoid excess enzymes to affect the oxidation rate, the reaction is performed in an ice bath.

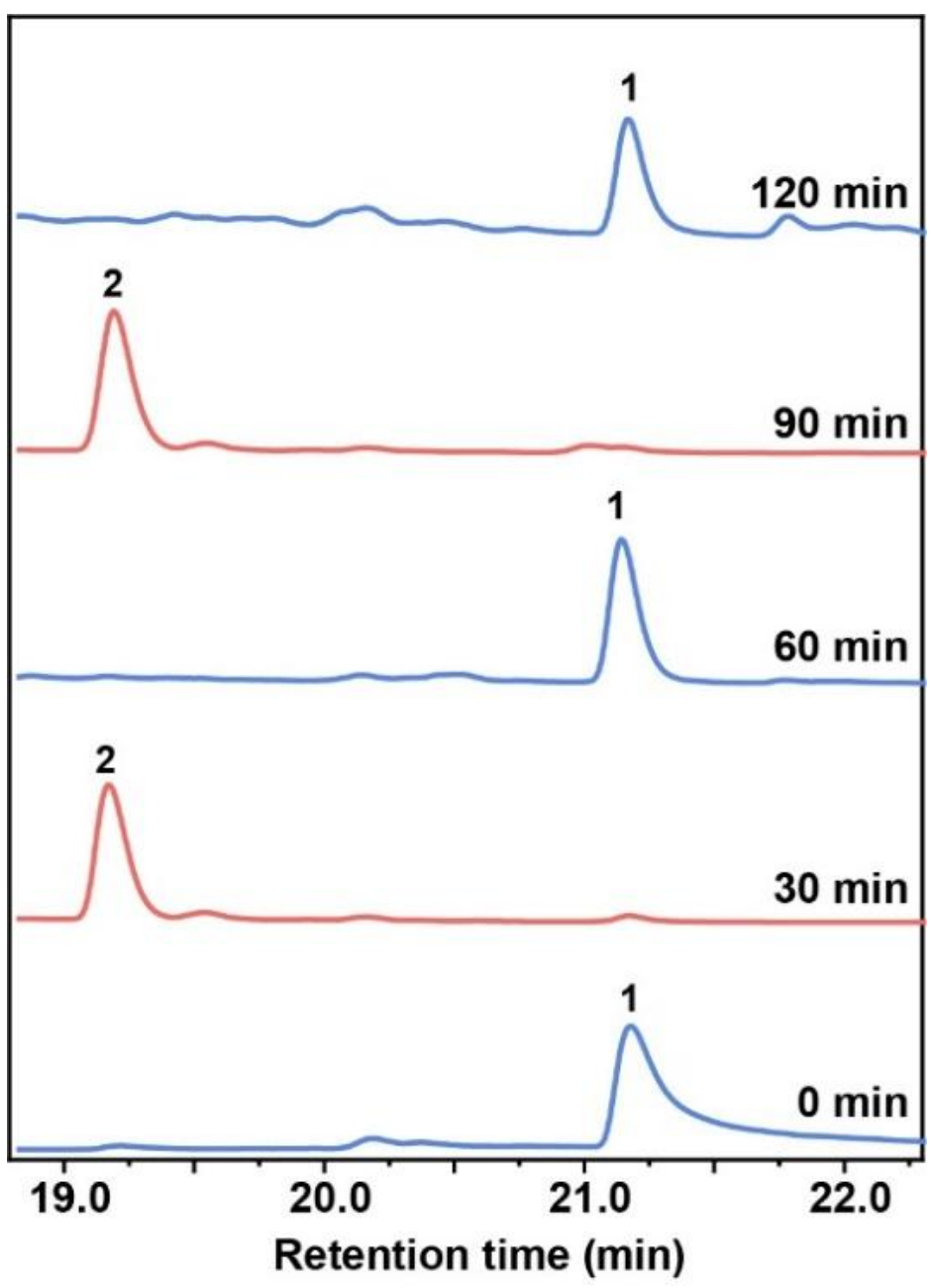

Supplementary Figure 15. Kinetic analysis over multiple redox-medicated cycles between compound **1** and compound **2** by using analytical-HPLC.

9. Quantitative analysis of reactive oxygen species (ROS) generation

First, singlet oxygen production was evaluated using Singlet Oxygen Sensor Green (SOSG). A progressive increase in fluorescence intensity was observed with each successive light exposure, indicating continuous generation of singlet oxygen ($^1O_2$). We then assessed overall ROS levels using the general ROS probe 2’,7’-dichlorofluorescein diacetate (DCFH-DA), which exhibited a similar increasing trend, further confirming light-induced ROS formation.

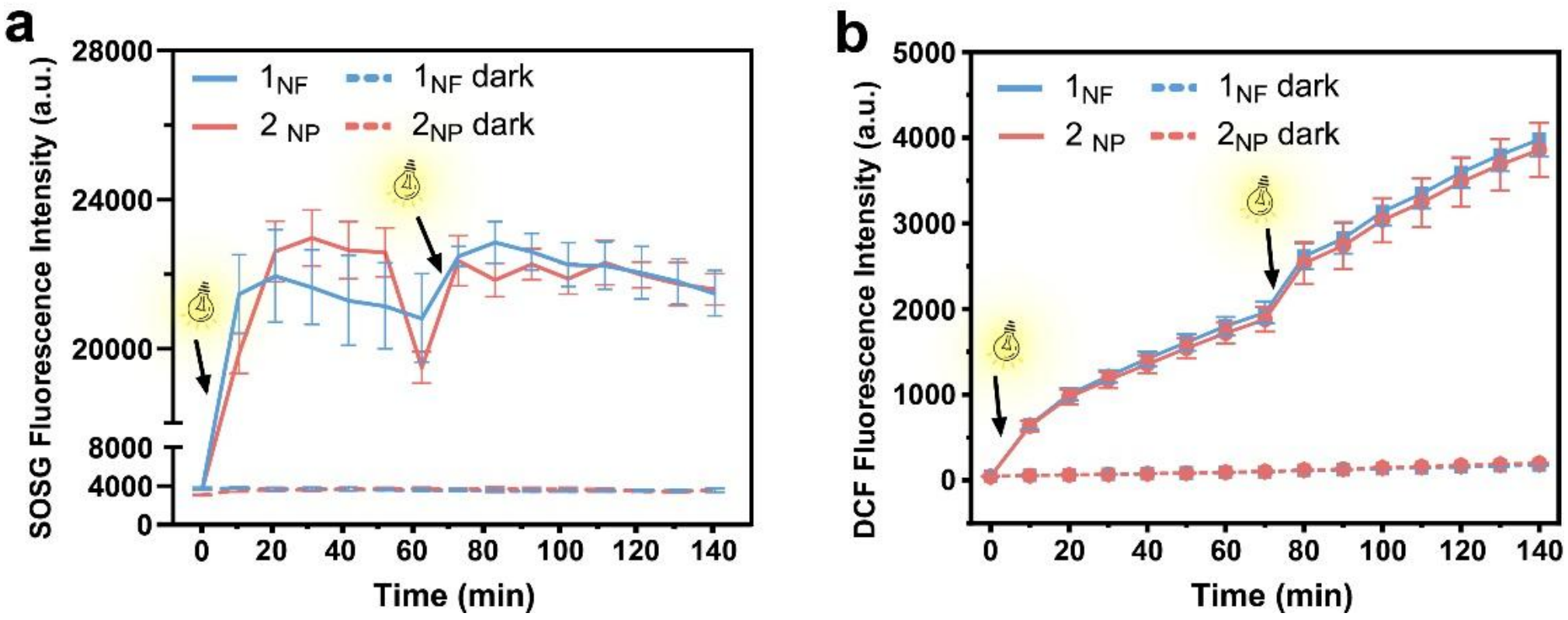

Supplementary Figure 16. a. SOSG fluorescence intensity of $^1O_2$ generated by light-irradiated $\mathbf{1}_{\mathbf{NF}}$ and $\mathbf{2}_{\mathbf{NP}}$ at 200 µM. The mean of n = 3 biological samples ± s.d. is shown. b. Total ROS levels (DCF fluorescence intensity) by light-irradiated $\mathbf{1}_{\mathbf{NF}}$ and $\mathbf{2}_{\mathbf{NP}}$ at 200 µM. Data are represented as the mean ± s.d. (n= 3 independent replicates) The arrow and light bulb represent the 10 min-light irradiation at t = 0 min and 70 min.

10. Critical aggregation concentrations (CAC) of compound 1 and compound 2.

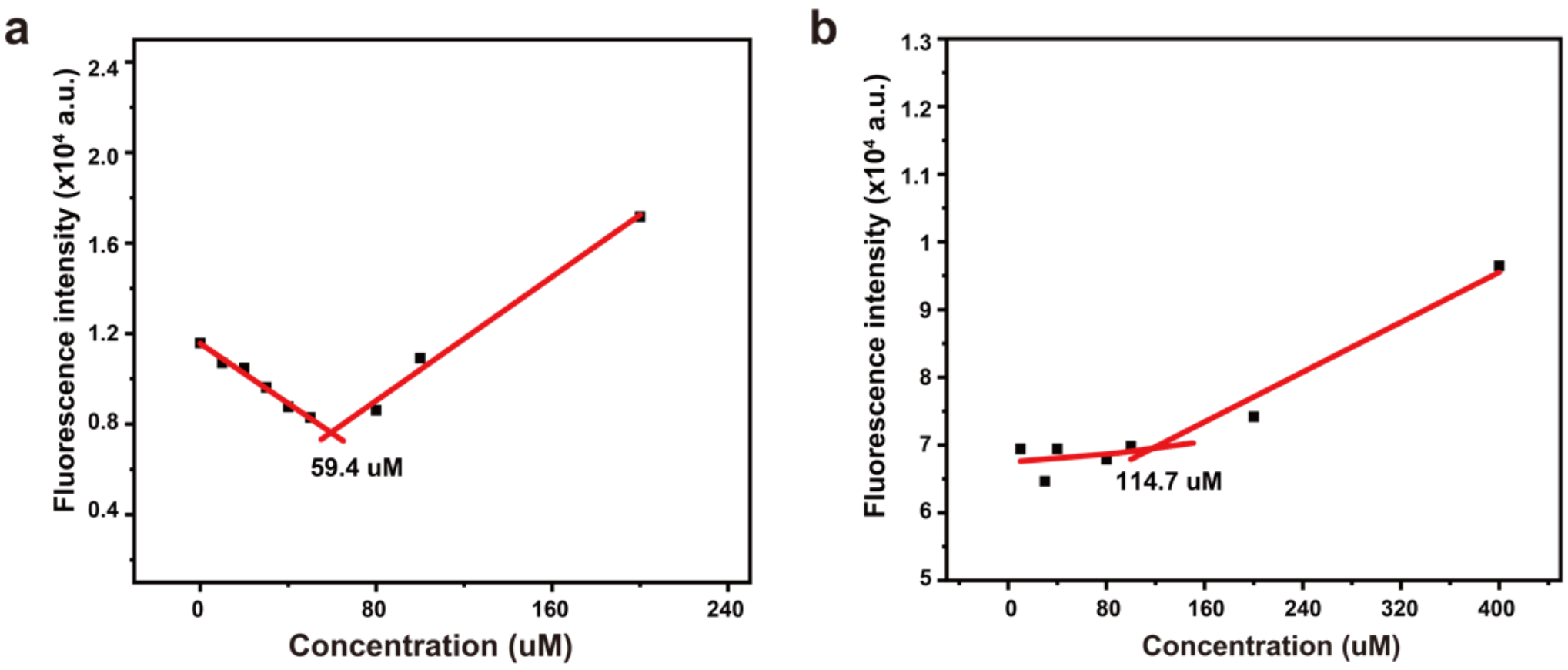

Supplementary Figure 17. a. ThT fluorescence intensity *versus* concentration of compound **1** and b. compound **2** in AB buffer at 37ºC.

11. Optical properties of 1 and 2.

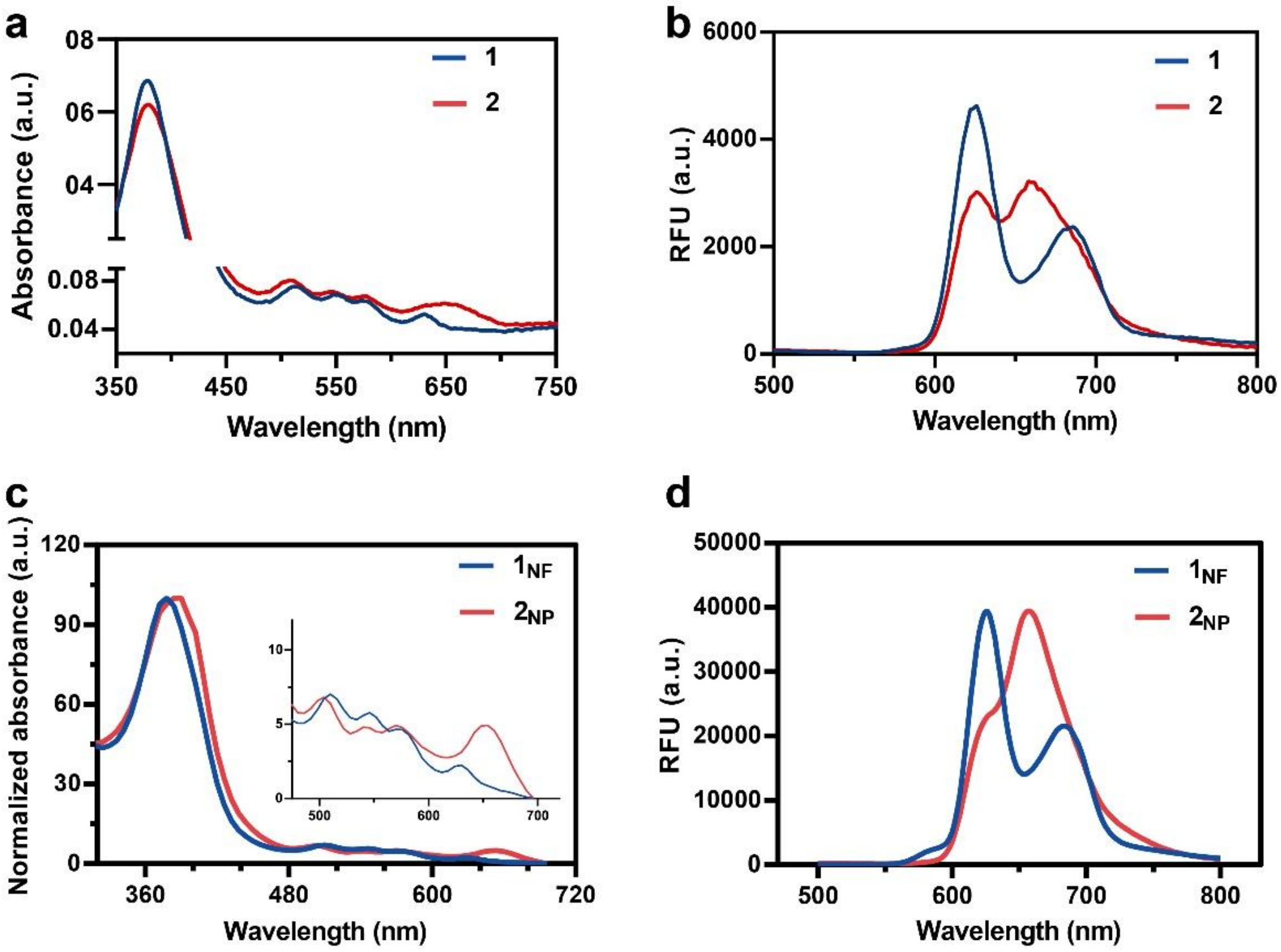

Supplementary Figure 18. a. UV–vis absorption spectra of Compounds **1** and **2** (50 µM, AB buffer) b. Fluorescence emission spectra of Compounds **1** and 2 (50 µM, AB buffer; λex = 410 nm). c. UV–vis absorption spectra of assembly states 1_{NF} and $\mathbf{2_{NP}}$ (200 µM, AB buffer) d. Fluorescence emission spectra of assembly states $\mathbf{1_{NF}}$ and $\mathbf{2_{NP}}$ (200 µM, AB buffer; λex = 410 nm).

12. The hydrodynamic diameter of 2_{NP}

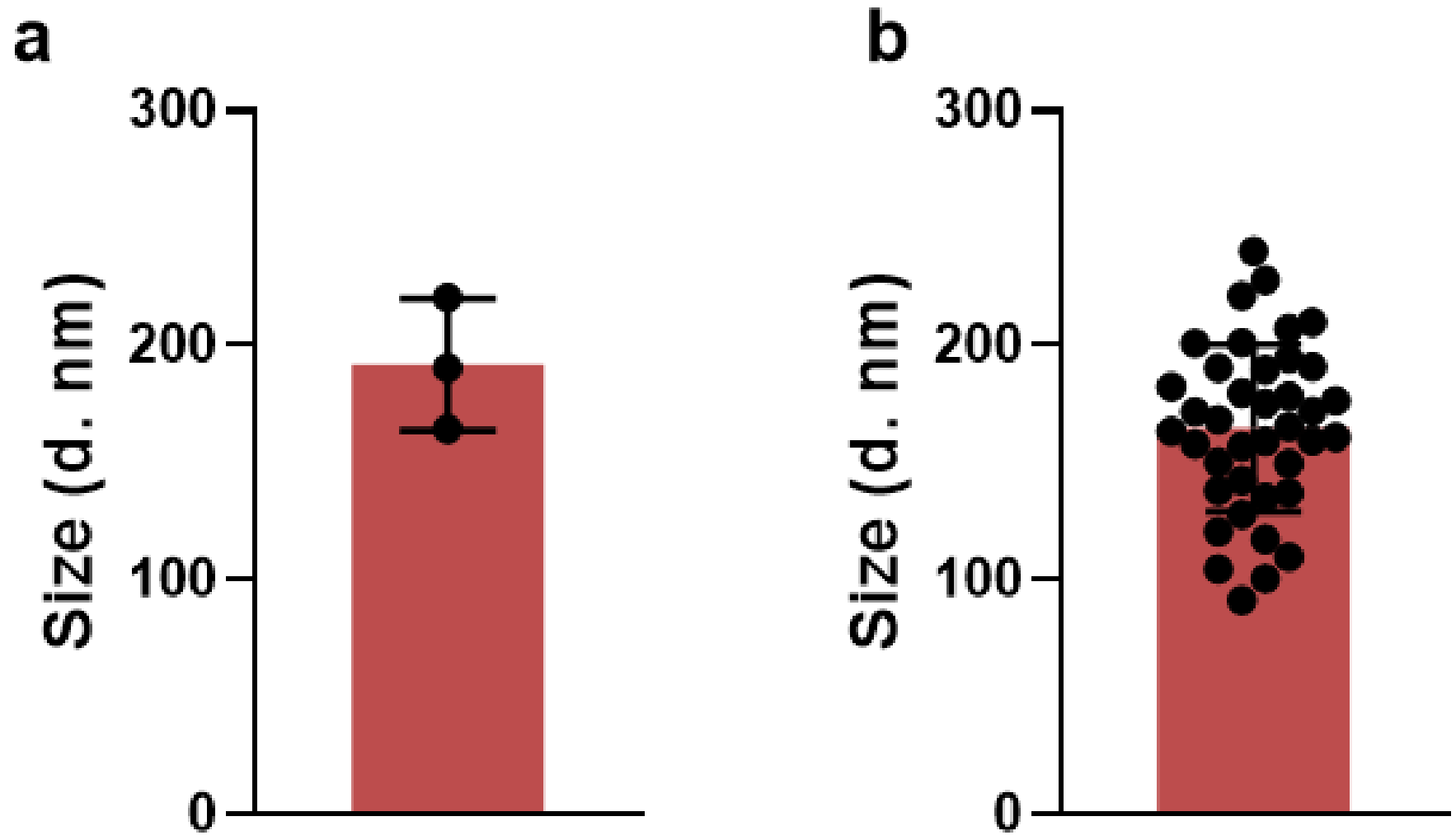

Supplementary Figure 19. a. The intensity-average hydrodynamic diameter of $\mathbf{2_{NP}}$ at 200 µM in AB buffer as measured by DLS. The diameter was determined to be 192 ± 28 nm. Data were obtained from three independent replicates are presented as mean ± s.d. b. The average diameter was calculated to be 166 ± 35 nm by measuring nanoparticles from TEM images using ImageJ software. Data are represented as the mean ± s.d. (n= 3 independent experiments)

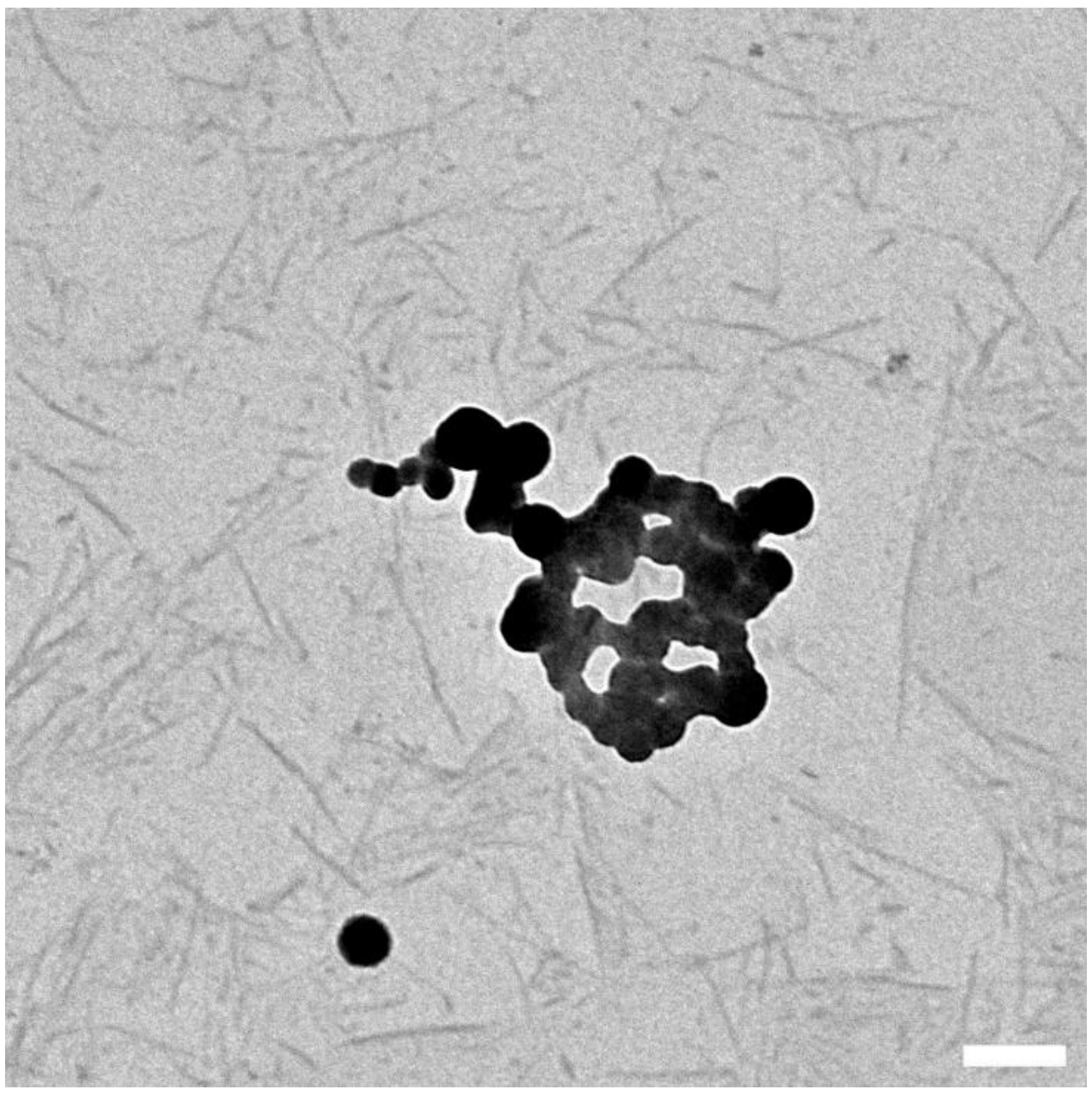

Supplementary Figure 20. The intermediate state after 10 min irradiation and 10 min sample preparation observed by TEM (Scale bar = 400 nm). Experiments were independently repeated three times with similar results.

13. Secondary structures

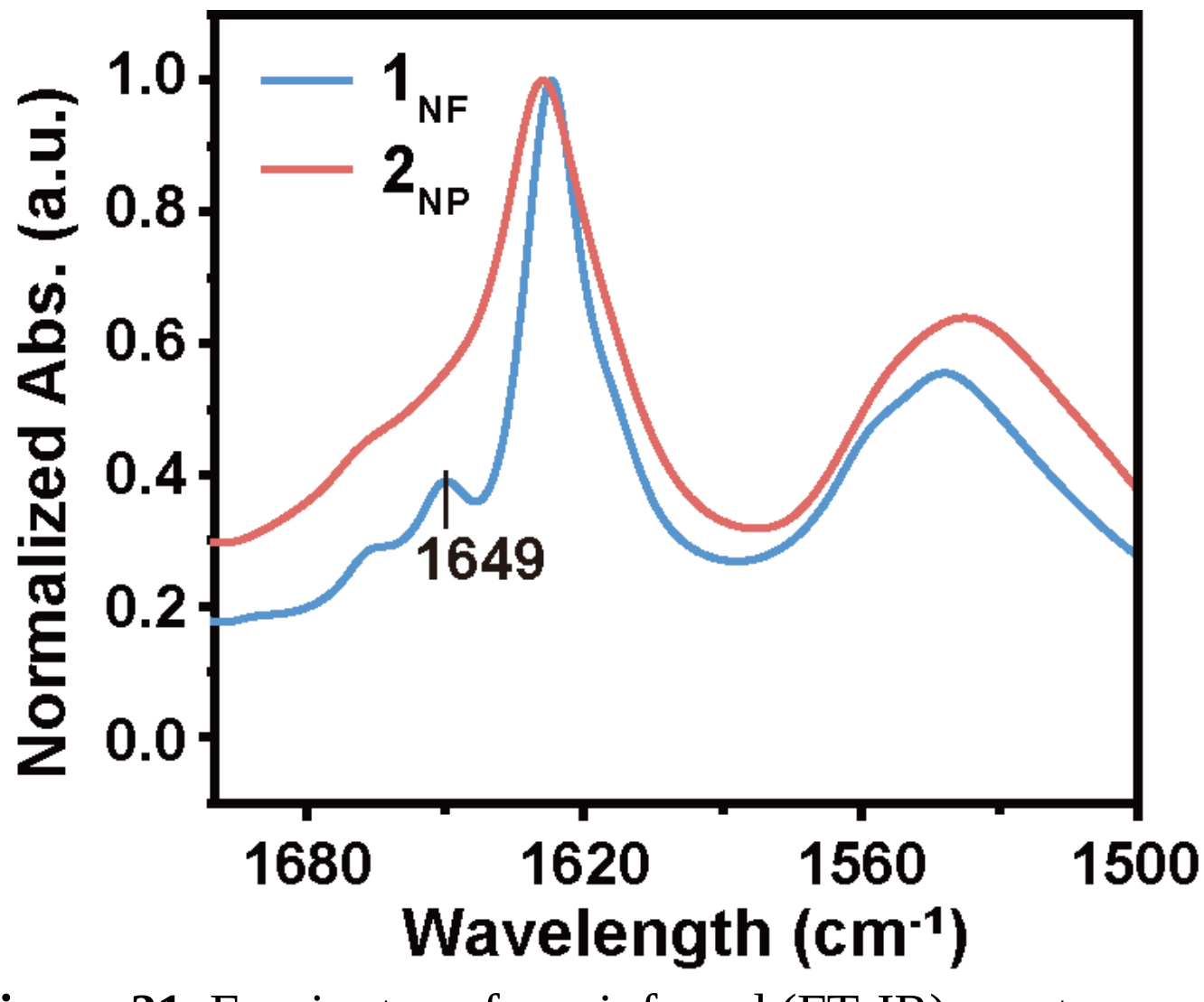

Supplementary Figure 21. Fourier transform infrared (FT-IR) spectroscopy of $\mathbf{1_{NF}}$ and $\mathbf{2_{NP}}$ solid powders

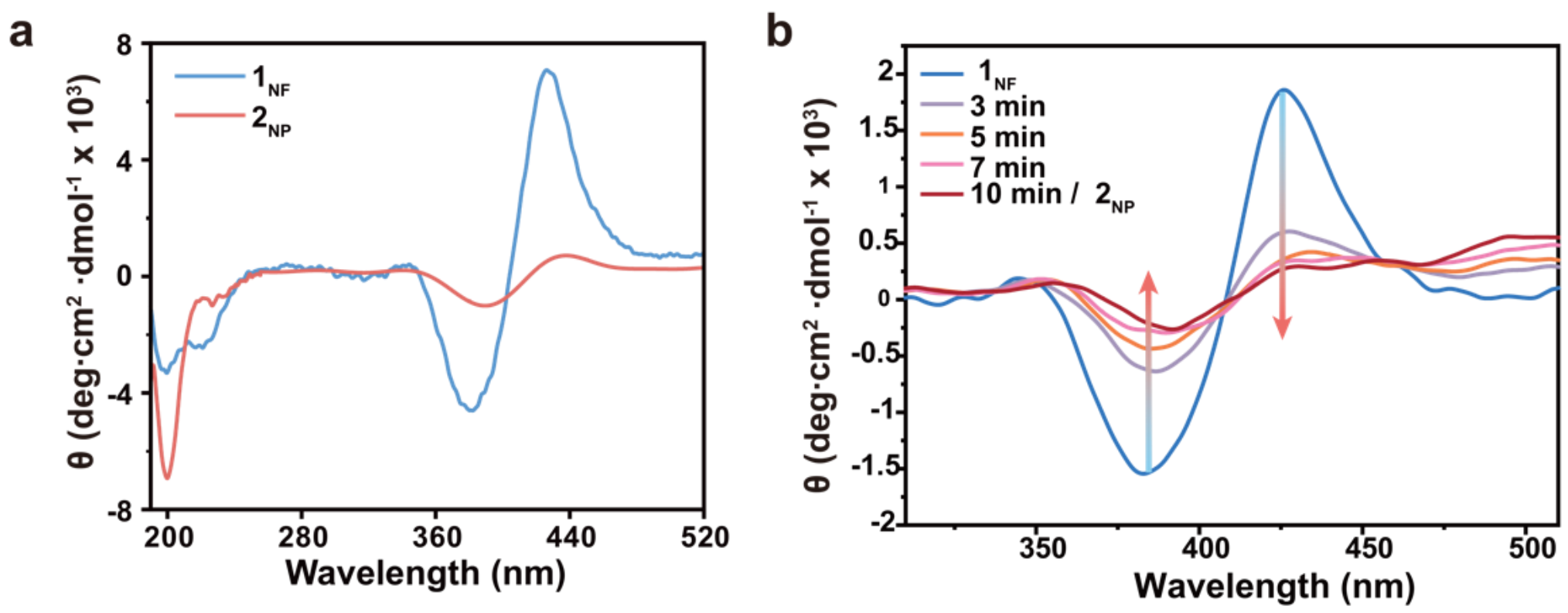

Supplementary Figure 22. a. Circular dichroism (CD) spectra of $\mathbf{1}_{\mathbf{NF}}$ and $\mathbf{2}_{\mathbf{NP}}$ (200 μM) in AB buffer. b. The CD spectra of $\mathbf{1}_{\mathbf{NF}}$ (200 μM) in AB buffer were obtained from the Soret region following light irradiation.

14. Phasor- FLIM to monitor assembly progression and assemblies' transformation

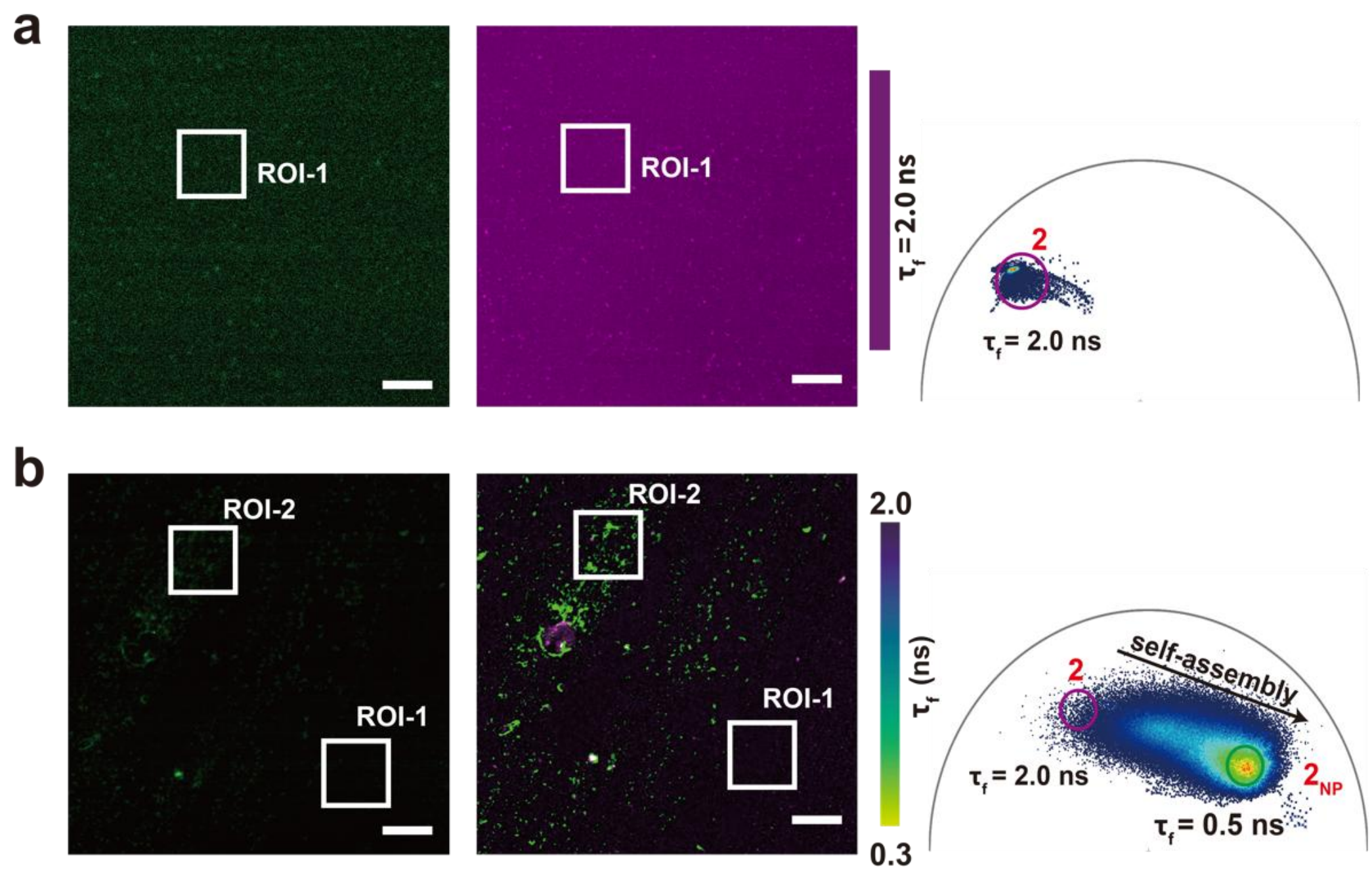

Supplementary Figure 23. a. Intensity-based confocal image for compound **2** (50 μM) in a cell-free condition (left). λex = 488 nm, λem = 600 - 700 nm. Scale bar, 20 μm. Corresponding fluorescence lifetime image of it. λex = 488 nm (middle). The magenta signal indicates monomers (ROI-1) with τ_f = 2.0 ns shown in the phasor map (right). b. The aggregates formation was tracked by Phasor-FLIM. Intensity-based confocal image for $\mathbf{2}_{\mathbf{NP}}$ (200 μM) in a cell-free condition (left). λex = 488 nm, λem = 600 - 700 nm. Scale bar, 20 μm. Corresponding fluorescence lifetime image of it. λex = 488 nm (middle). The magenta signal indicates monomers (ROI-1) with τ_f = 2.0 ns shown in the phasor map (magenta circle). And the green signal indicates aggregates (ROI-2) with $\mathbf{2}_{\mathbf{NP}}$ τ_f = 0.5 ns shown in the phasor map (green circle). Scale bar, 20 μm. Each experiment in a and b was performed with three biological replicates, yielding similar results.

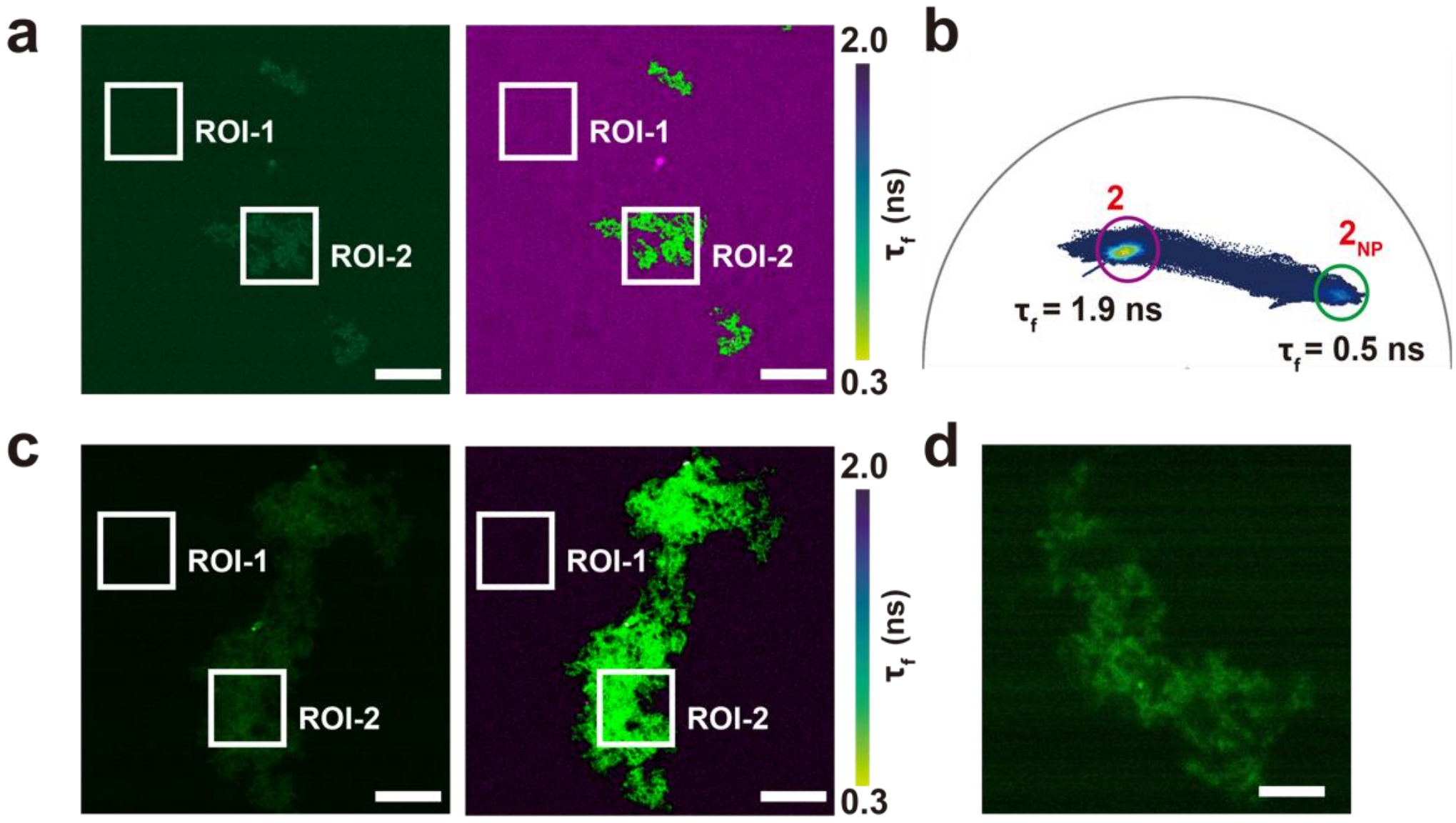

Supplementary Figure 24. a. Intensity-based confocal image (left) for $\mathbf{1_{NF}}$ in a cell-free condition after 3 min irradiation. λex = 488 nm, λem = 600 - 700 nm. Scale bar, 20 μm. Corresponding fluorescence lifetime image of it (right). λex = 488 nm. The magenta signal indicates monomers (ROI-1) with τ_f = 1.9 ns, and the green signal indicates aggregates (ROI-2) with $\mathbf{2_{NP}}$ τ_f = 0.5 ns. Scale bar, 10 μm. b. Fluorescence lifetime phasor map of $\mathbf{1_{NF}}$ in a cell-free condition after 3 min irradiation. Two major species showing intermediates τ_f = 1.9 ns and $\mathbf{2_{NP}}$ τ_f = 0.5 ns can be identified. c. Intensity-based confocal image (left) for $\mathbf{1_{NF}}$ in a cell-free condition after 5 min irradiation. λex = 488 nm, λem = 600 - 700 nm. Scale bar, 20 μm. Corresponding fluorescence lifetime image of it (right). λex = 488 nm. The magenta signal indicates monomers (ROI-1) with τ_f = 1.9 ns, and the green signal indicates aggregates (ROI-2) with $\mathbf{2_{NP}}$ τ_f = 0.5 ns. Scale bar, 10 μm (Right) D. Intensity-based confocal image for $\mathbf{1_{NF}}$ in a cell-free condition after 10 min irradiation. λex = 580 nm, λem = 600 - 700 nm. Scale bar, 5 μm. Each experiment was performed with three biological replicates, yielding similar results.

15. Bacterial viability

Due to solubility constraints, the compound concentrations were capped at 200 µM, and the culture incubation time was limited to 12 hours. Beyond these conditions, precipitation occurred, which could interfere with an accurate toxicity assessment. Therefore, all subsequent experiments (200 µM, 10-15 min irradiation and 12 h culture) were conducted within this soluble and stable range to ensure reliable results.

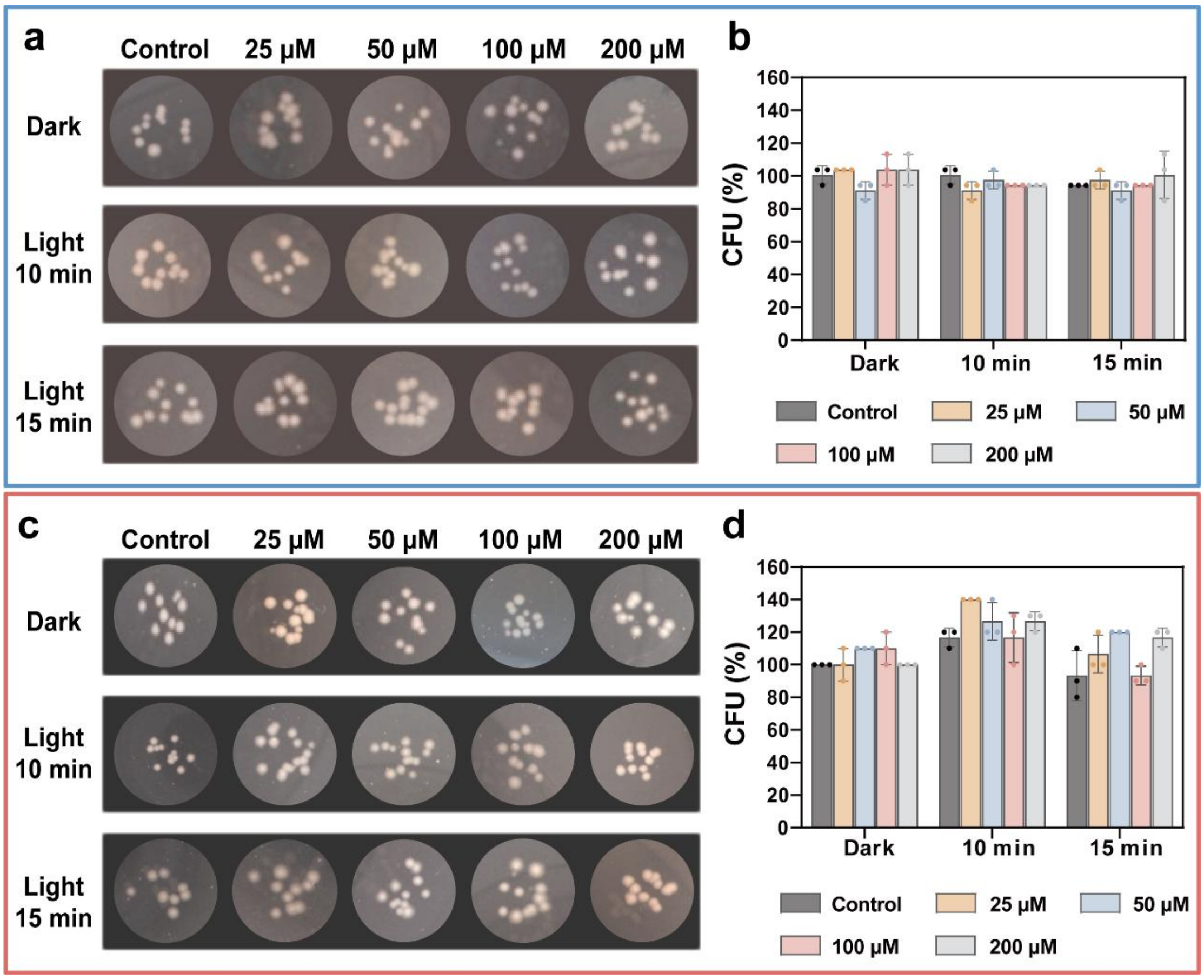

Supplementary Figure 25. Bacterial colony counting assay. a. Corresponding optical images of the number of *E. coli* GFP colonies treated with varying concentrations of $\mathbf{1_{NF}}$ after 12 h on agar plates. b. Colony-forming unit (CFU) showing bacterial viability with the indicated treatments after 12 hours. The results are presented relative to the media-dark control (100% viable). The mean values from three independent experiments are shown with the error bars representing the standard deviation. Data are represented as the mean ± s.d. (n= 3 independent biological replicates) c. Corresponding optical images of the number of *E. coli* GFP colonies treat with $\mathbf{2_{NP}}$ after 12 h on agar plates. The assemblies are mixed with bacteria-containing solutions at a 1:1 volume ratio. d. CFU showing bacterial viability with the indicated treatments after 12 hours. The results are presented relative to the media-dark control (100% viable). The mean values from three independent experiments are shown with the error bars representing the standard deviation. Data are represented as the mean ± s.d. (n= 3 independent biological replicates) Experiments in a and c were independently repeated three times with similar results.

16. Time-dependent and morphological-induced bacterial internalization

16.1 Gating strategies for *E. coli* GFP

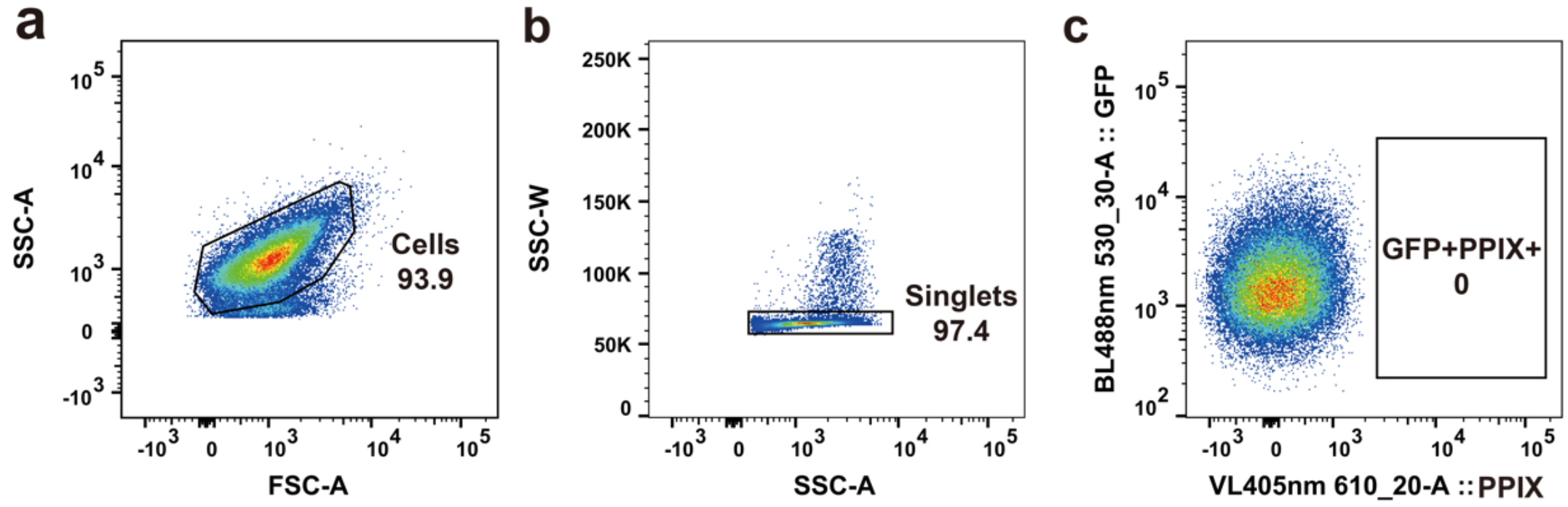

Supplementary Figure 26. Gating strategies used for flow cytometry analysis of *E. coli* GFP bacteria taking-up of assemblies. A. FSC-A vs SSC-A dot plots showing the gating of cells b. SSC-A vs SSC-W dot plots showing the gating of singlets (gate at cells) c. PPIX vs GFP dot plots showing the gating of GFP+PPIX+ (gate at singlets).

16.2 Time-dependent bacterial internalization and quantification.

E. coli GFP and fluorophore-coupled peptide conjugates are suitable for dual fluorescence mode flow cytometry. With a proper gating strategy, at singlets gate the percentage of dual signal data revealed morphological-induced and time-dependent bacterial internalization. Interestingly, **1**$_{NF}$ showed negligible internalization (2.7%), while **1**$_{NF}$ **+ light** and **2**$_{NP}$ demonstrated increased uptake, peaking at 14.45% and 26.52%, respectively, after 8 hours. This time-dependent internalization pattern was consistent across all groups. Additionally, after 8 hours **1**$_{NF}$ **+ light** achieves a similar uptake percentage to **2**$_{NP}$ at 4h. (Supplementary Fig. 27)

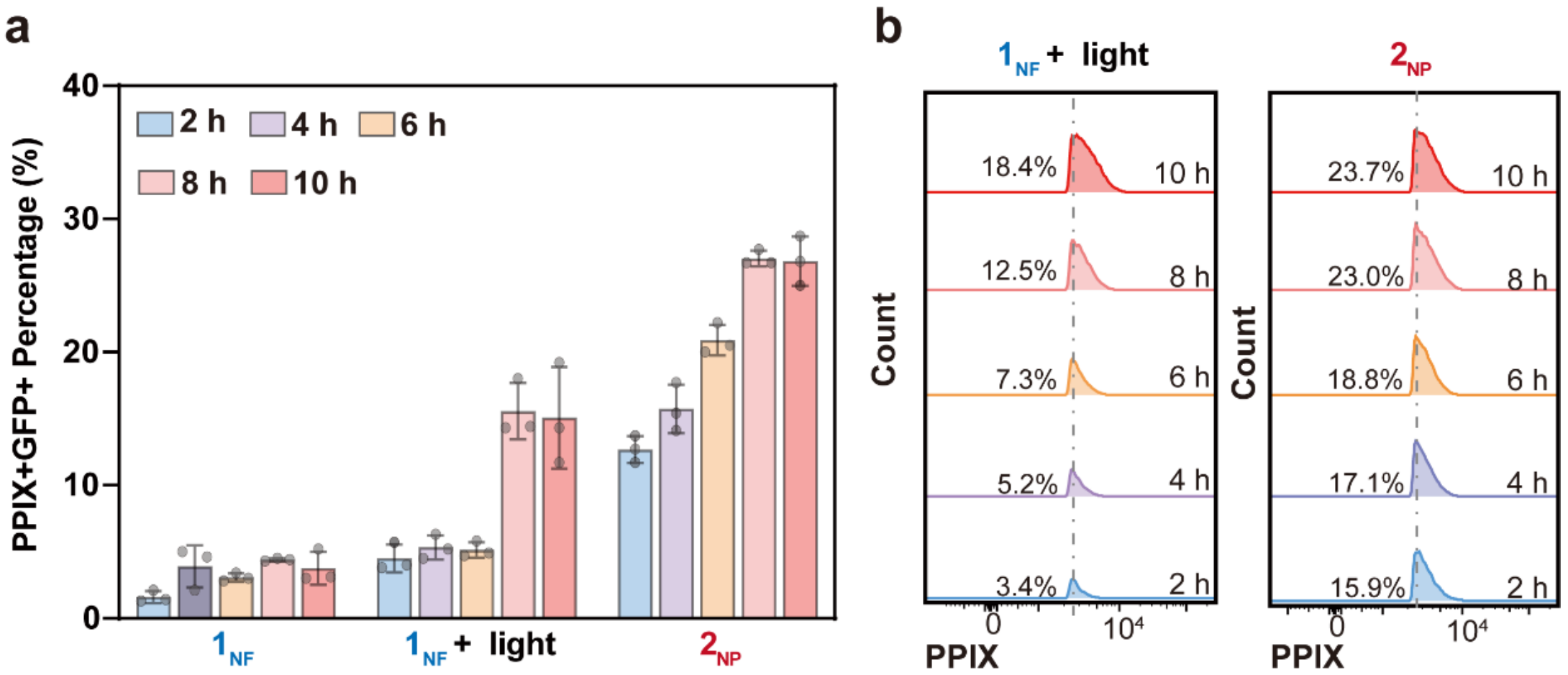

Supplementary Figure 27. a. Dual fluorescence analysis of flow cytometry for quantifying bacterial internalization gating at PPIX+GFP+. Data are represented as the mean ± s.d. (n= 3 independent biological replicates) b. Histograms obtained from flow cytometry analysis. Comparison histograms of PPIX+ counts at different time intervals gating at PPIX+GFP+.

To enable visualization of the assemblies and further analyze their uptake behavior via CLSM, both $\mathbf{1}_{NF}$ and $\mathbf{2}_{NP}$ are co-assembled with sulforhodamine 101-coupled with control peptides. After co-assembly both co$\mathbf{1}_{NF}$ and co$\mathbf{2}_{NP}$ preserved the same morphology as depicted in TEM images, indicating that fluorescent labeling does not interfere with structure formation (Supplementary Fig. 28). It should be noted that this method was only applied for the GFP strain in order to avoid the overlay of PPIX signals. Therefore, no further distinction is made in the main text.

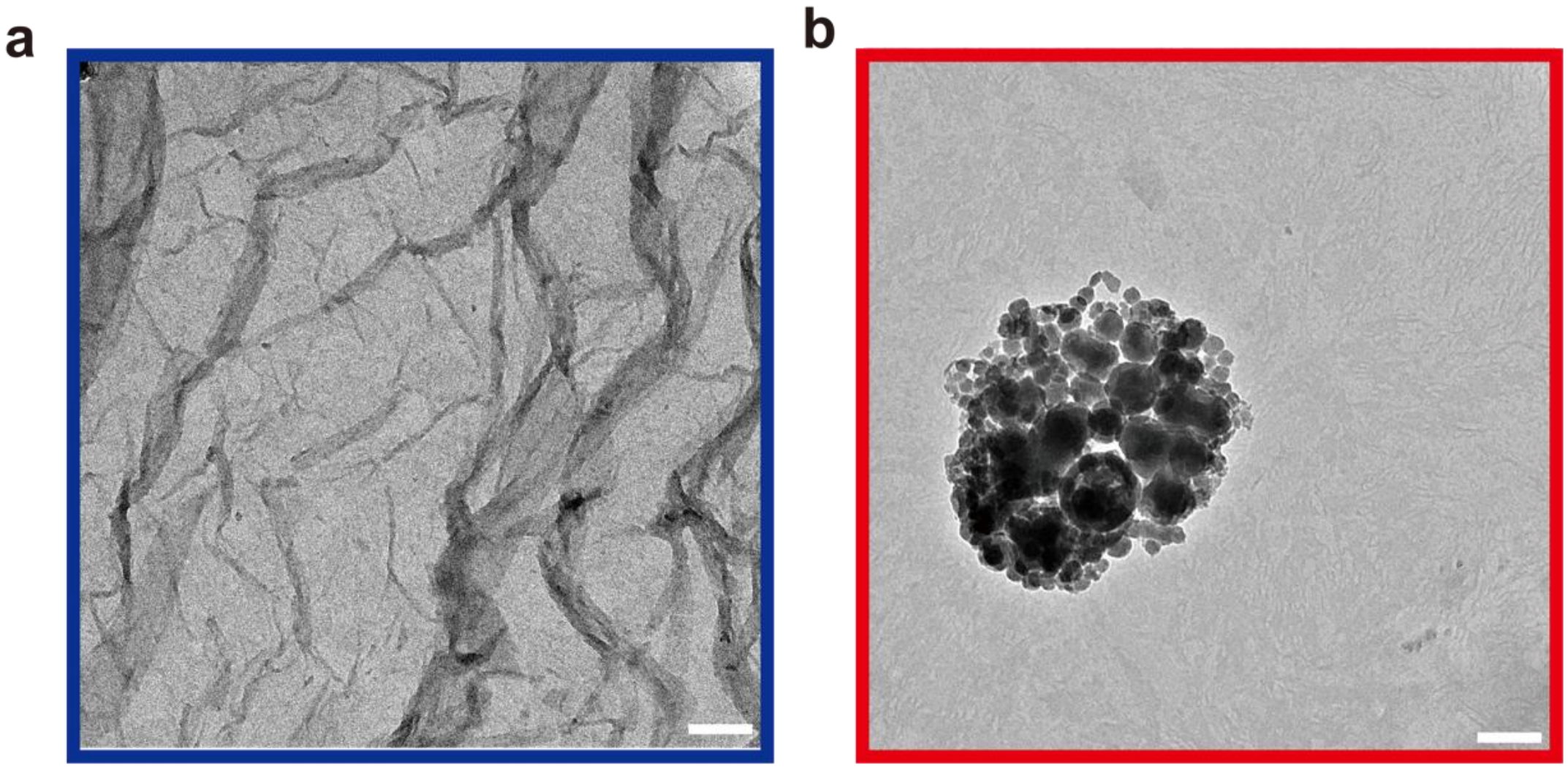

Supplementary Figure 28. a. TEM images of co$\mathbf{1}_{NF}$ (200 µM, 10% sulforhodamine 101-DVMVMD) in AB buffer. Scale bar, 200 nm. b. TEM images of co$\mathbf{2}_{NP}$ (200 µM, 10% sulforhodamine 101-DVM^{o}VM^{o}D) in AB buffer. Scale bar, 400 nm. Experiments in a and b were independently repeated three times with similar results.

Flow cytometry and co-localization analysis of both GFP and PPIX channels CLSM images are used to quantify bacterial internalization (Fig. 4a and Supplementary Fig. 26). The differences between $\mathbf{1_{NF}}$ and $\mathbf{1_{NF}}$ **+ light** validated the importance of morphology in preferential uptake.[1] The population distribution of 4 groups at 8 h displayed the shift to the PPIX+GFP+ region of particle-shaped groups (Supplementary Fig. 29).

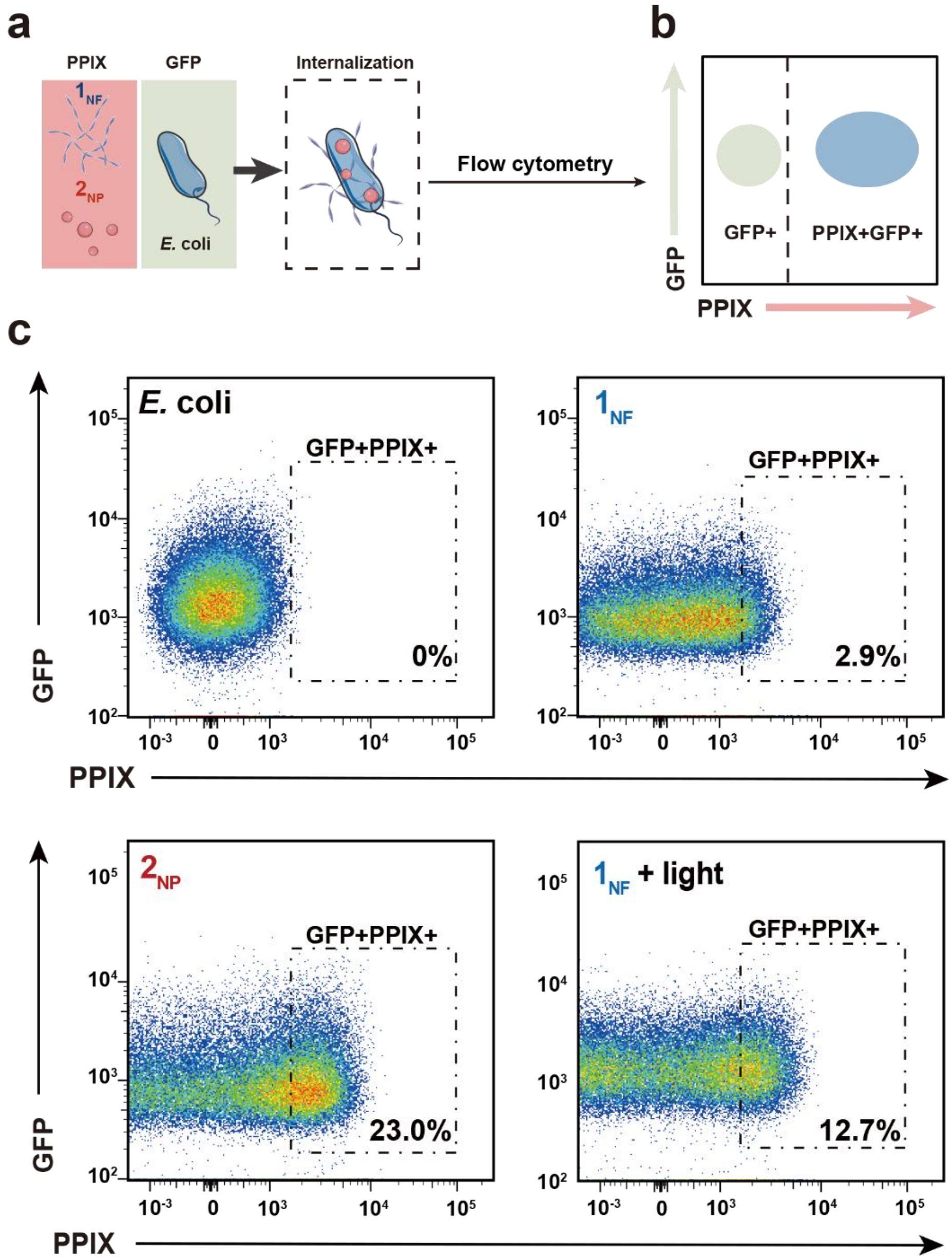

Supplementary Figure 29. Morphology-induced bacterial uptake. a. Schematic of co-culture *E. coli* with $\mathbf{1_{NF}}$ and $\mathbf{2_{NP}}$ separately, resulting in morphology-related bacterial uptake of $\mathbf{2_{NP}}$ but not of $\mathbf{1_{NF}}$. b. Bacterial uptake was also quantified by dual fluorescence mode flow cytometry. c. Representative flow cytometry population plots of GFP+PPIX+ bacterial cells after 8 h incubation and quantification, gated at singlets under different treatments.

17. Temperature-dependent nanoparticle internalization.

To elucidate the uptake mechanism of nanoassemblies, we examined its temperature dependence. An inhibition assay was performed at 4 °C for 8 h to suppress metabolic activity. Notably, intracellular fluorescence signals from the positive groups (**1**$_{NF}$ + light and **2**$_{NP}$) were negligible at this low temperature. This significant reduction confirms that the internalization is a temperature-dependent process, implying that the particles are taken up via an energy-dependent active transport mechanism.

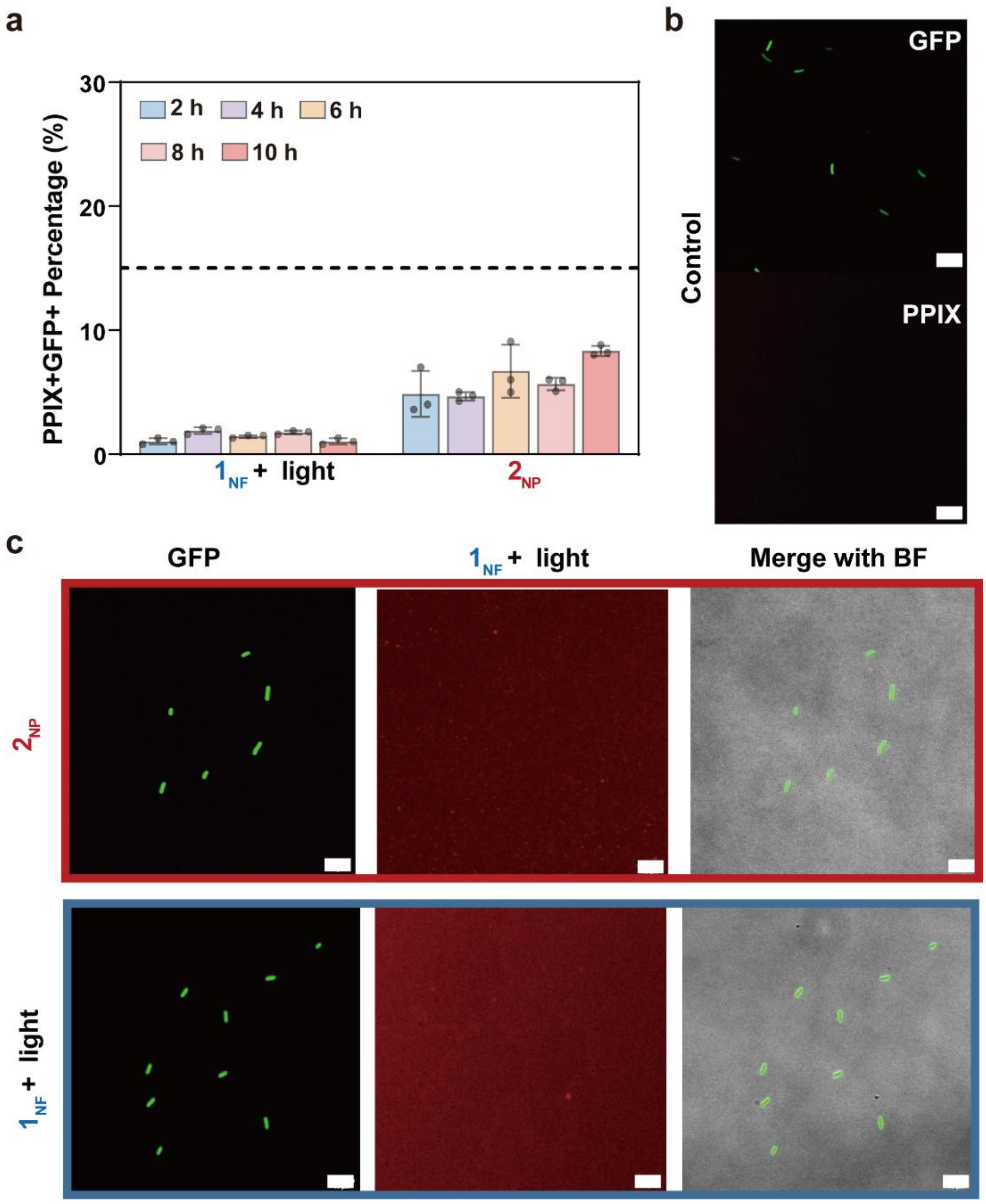

Supplementary Figure 30. Temperature-dependent nanoparticle internalization. a. Flow cytometry analysis of **1**$_{NF}$ **+ light** and **2**$_{NP}$ bacterial internalization after 2, 4, 6, 8, and 10 h incubation with *E. coli* GFP at 4°C. (Gated at singlets) The dotted line is the average of the uptake percentage at 37°C. Data are represented as the mean ± s.d. (n = 3 independent biological replicates) b. CLSM images of *E. coli* GFP bacterial cells untreated after 8 h at 4°C. Scale bars, 10 µm. c. CLSM images of *E. coli* GFP bacterial cells treated with co**2**$_{NP}$ (200 µM) and co**1**$_{NF}$ **+ light** (200 µM) with light after 8 h. Scale bars, 10 µm. Experiments in b and c were independently repeated three times with similar results.

18. Energy-dependent nanoparticle internalization.

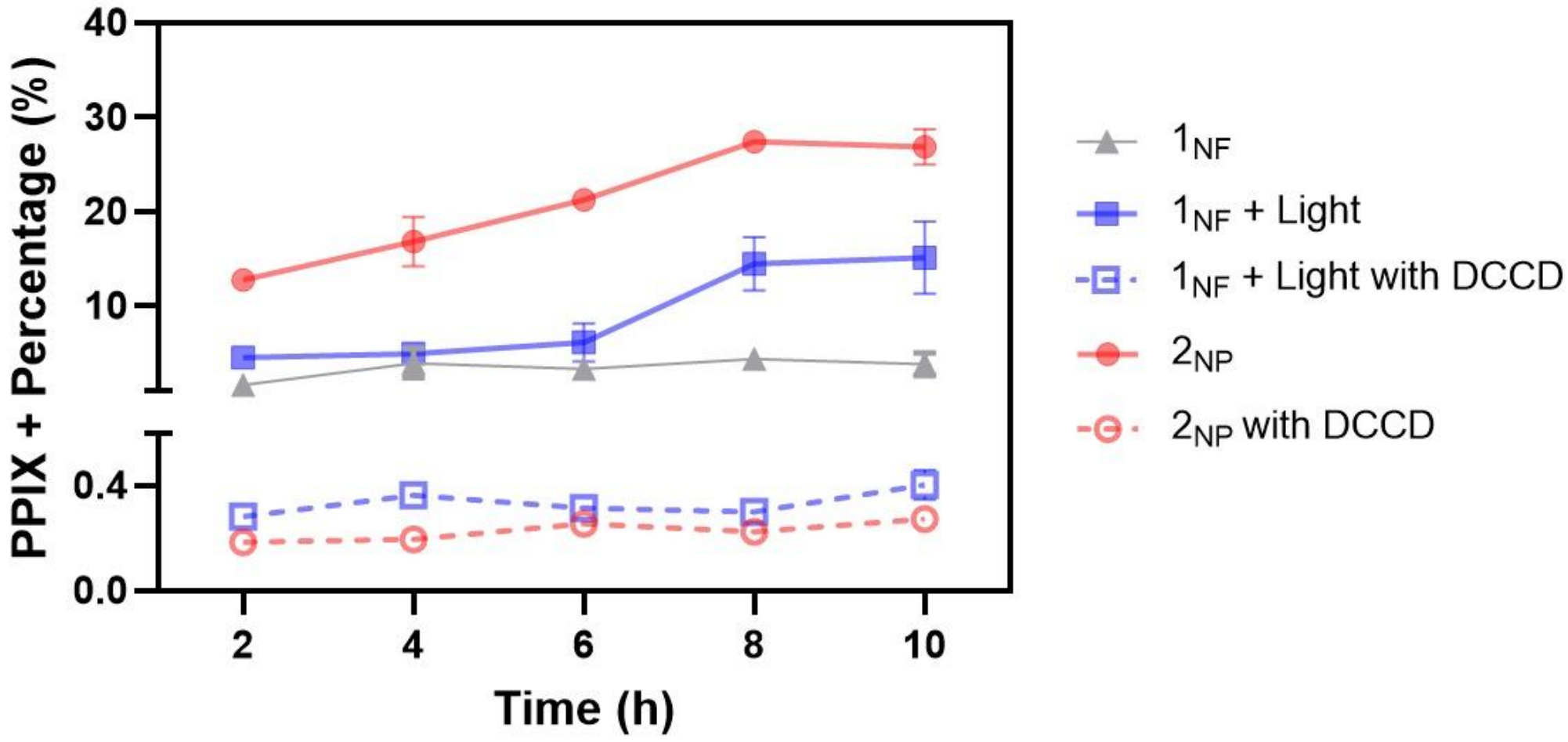

Supplementary Figure 31. Flow cytometry analysis of "**1**$_{\mathbf{NF}}$ + Light" and **2**$_{\mathbf{NP}}$ bacterial internalization after 2, 4, 6, 8, and 10 h incubation with *E. coli* GFP with and without ATP synthase inhibitor, DCCD. (Gated at singlets) Data are represented as the mean ± s.d. (n= 3 independent biological replicates)

19. Membrane integrity.

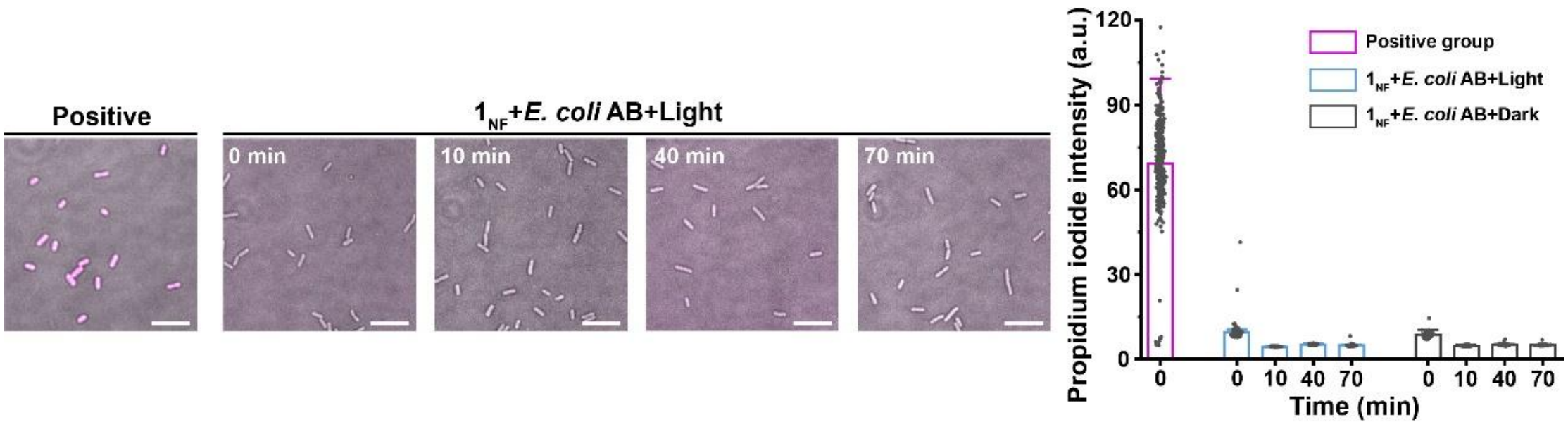

Supplementary Figure 32. Membrane integrity detected by propidium iodide (PI) staining. Representative fluorescence/bright-field merged images of *E. coli* AB from two groups: 1) positive control group, in which *E. coli* AB were treated with 70% isopropanol. 2) experimental group, in which *E. coli* AB were co-incubated with **1**$_{\mathbf{NF}}$ and exposed to 10-minute light irradiation. Scale bar = 10 μm. Experiments were independently repeated three times with similar results. Quantitative analysis of propidium iodide (PI) fluorescence intensity calculated from the corresponding fluorescence images. Data are represented as the mean ± s.d. (n= 3 independent biological experiments)

20. Bacterial responses to oxidative stress

As shown in **Supplementary Figure** 3C, the bacteria exhibited sensitive bioluminescence production in response to environmental H_2O_2 levels. The bioluminescence intensities generated by **1**$_{NF}$ upon light irradiation is comparable to that of exogenously added 10 µM H_2O_2. This concentration was subsequently used to further verify the H_2O_2-activated MsrA-MsrB expression.

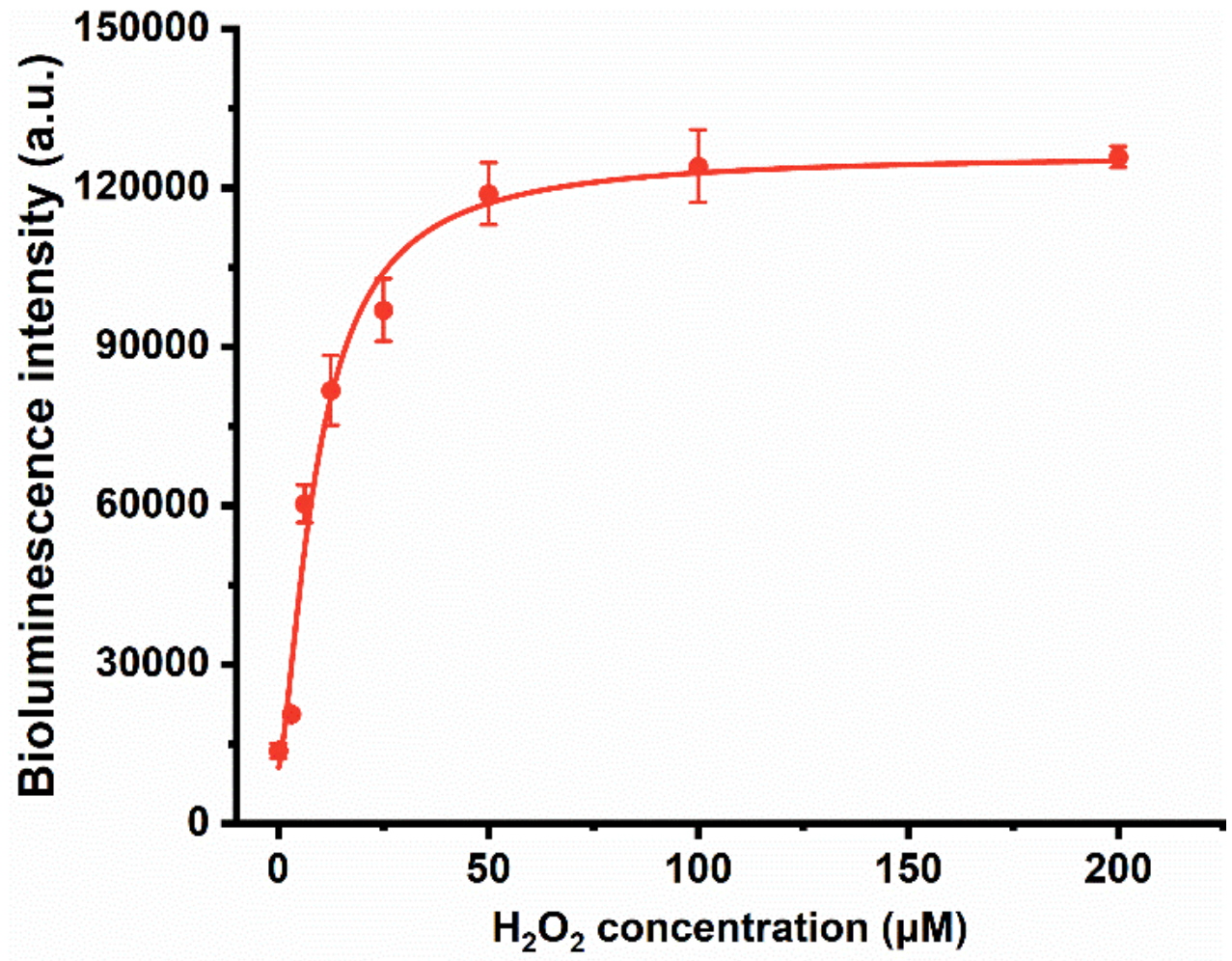

Supplementary Figure 33. Bioluminescence intensity in *E. coli* MG1655 transformed with the *oxyR-lux* plasmid, measured one hour after exposure to varying concentrations of H_2O_2. Data are represented as the mean ± s.d. (n= 3 independent biological replicates)

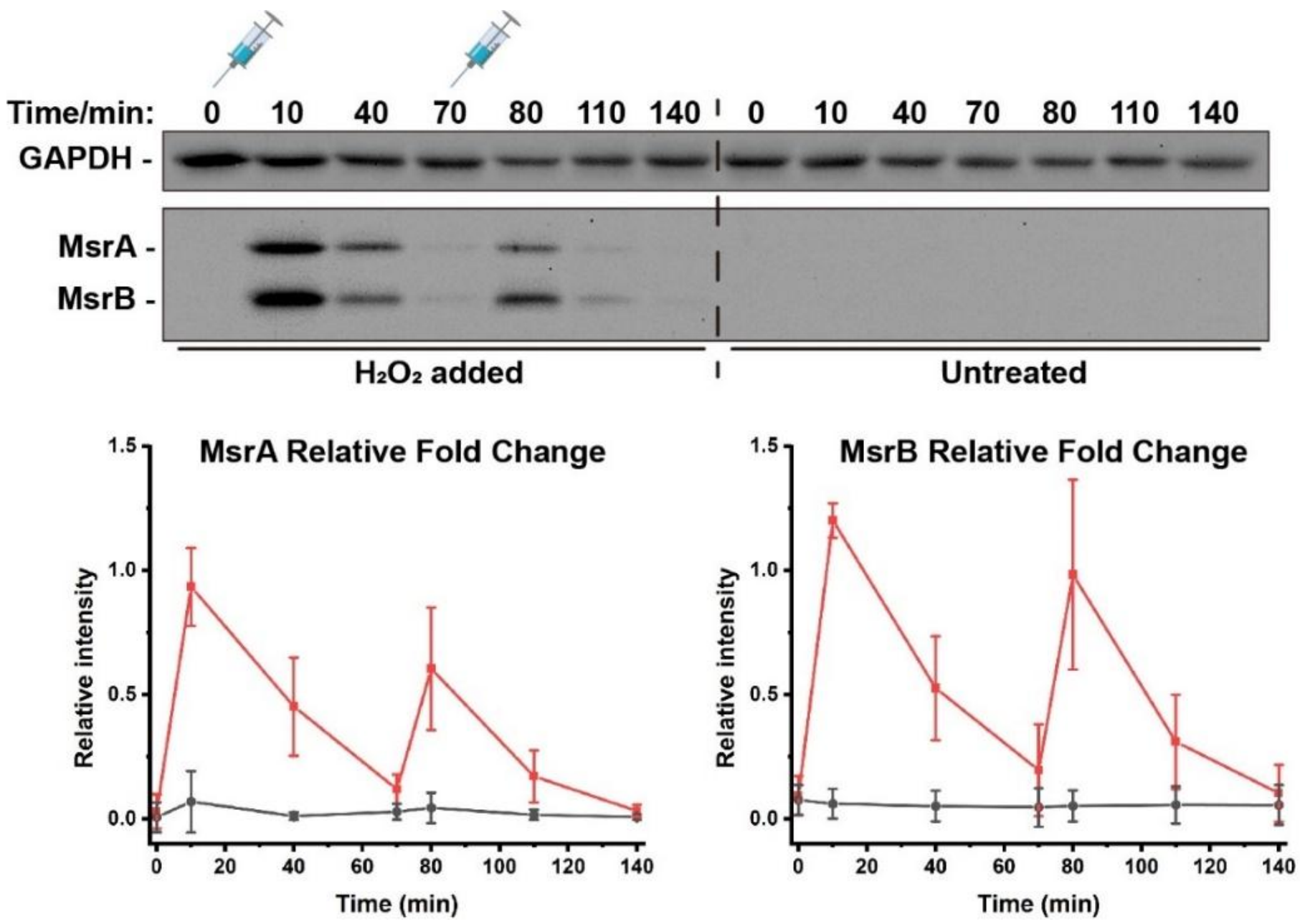

Supplementary Figure 34. Expression of MsrA and MsrB in *E. coli* MG1655 exposed to 10 µM H_2O_2 at 0 and 70 min, along with quantification of the band intensities from the immunoblots above. Data are represented as the mean ± s.d. (n= 3 independent biological replicates)

The reactivation behavior observed in Supplementary Fig. 35 serves as a positive control, demonstrating the effective regulatory function of our gene circuits and, more generally, indicating the significant potential to construct multiple redox cycles.

21. Bacterial viability across cycle-relevant time scales.

The bacterial colony counting assay was used to measure bacteria viability. The overnight bacteria culture was then further diluted to OD_{600} = 0.2 and mixed 1:1 (v/v) with **1_{NF}** solution (400 µM in AB buffer), yielding final concentrations of OD_{600} = 0.1 for bacteria and 200 µM for **1_{NF}**. At t = 0 min and t = 70 min, samples were irradiated for 10 min to induce photo-oxidation. At predetermined time points (t = 0, 10, 40, 70, 80, 110, 140 min), 100 µL bacteria-**1_{NF}** mixture was extracted and subjected to gradient dilution (10^5 times). After dilution, 2 µL of culture solution was placed on agar plates and incubated overnight for colony counting.

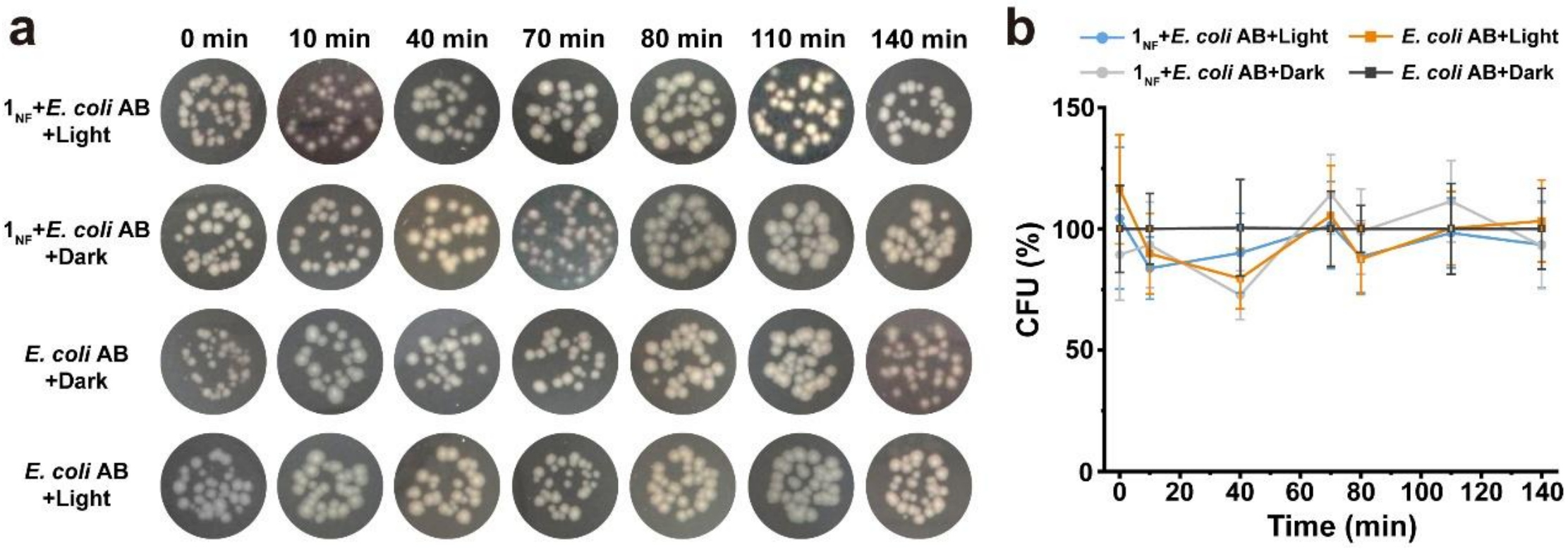

Supplementary Figure 35. Bacterial colony counting assay. (a) Representative images of *E. coli* AB colonies over cycles in the presence and absence of **1_{NF}**. Experiments were independently repeated three times with similar results. (b) Quantitative analysis of colony-forming unit (CFU) percentages for each group over the entire period, calculated by normalizing each group against the *E. coli* AB+Dark group. Data are represented as the mean ± s.d. (n= 3 independent biological replicates)

22. Phasor-FLIM for analyzing bacteria and material interactions

An integrated homeostasis loop was visualized using phasor-FLIM (Supplementary Fig. 33). Initially, in the absence of light, both monomer **1** (τ_f =1.1 ns) and assembly **1_{NF}** (τ_f =0.4 ns) were found around bacteria, corresponding to the fluorescence lifetime in the cell-free M9 medium (Fig. 3f and Supplementary Fig. 23 and 24). After 10 min illumination of **1_{NF}**, **2_{NP}** formed and favored by bacterial internalization. However, the fluorescence lifetime of species within the bacteria was slightly different to bacteria-free control experiments, likely due to the presence of MsrA and MsrB enzymes within the bacteria. More specifically, the τ_f of **2_{NP}** prolonged from 0.5 ns to 0.8 ns when located within bacteria (Supplementary Fig. 37). To confirm the role of enzymes, cell lysates of bacteria (Gene; *oxyR-msrA*+*oxyR-msrB* activated by 100 µM H_2O_2) were added to **2_{NP}** in the cell-free M9 medium as a reference, the enzyme and bacterial debris presence elongated the lifetime from 0.5 ns to 1.0 ns (Supplementary Fig. 38). These results confirmed that only the assembly state **2_{NP}**, but not **1_{NF}**, can be internalized into cells and subsequently cause perturbations. Moreover, because it is not possible to differentiate between the released **1_{NF}** and the initial non-uptaken version using phasor-FLIM, flow cytometry and CLSM were used to monitor the multiple homeostatic loops in the main text.

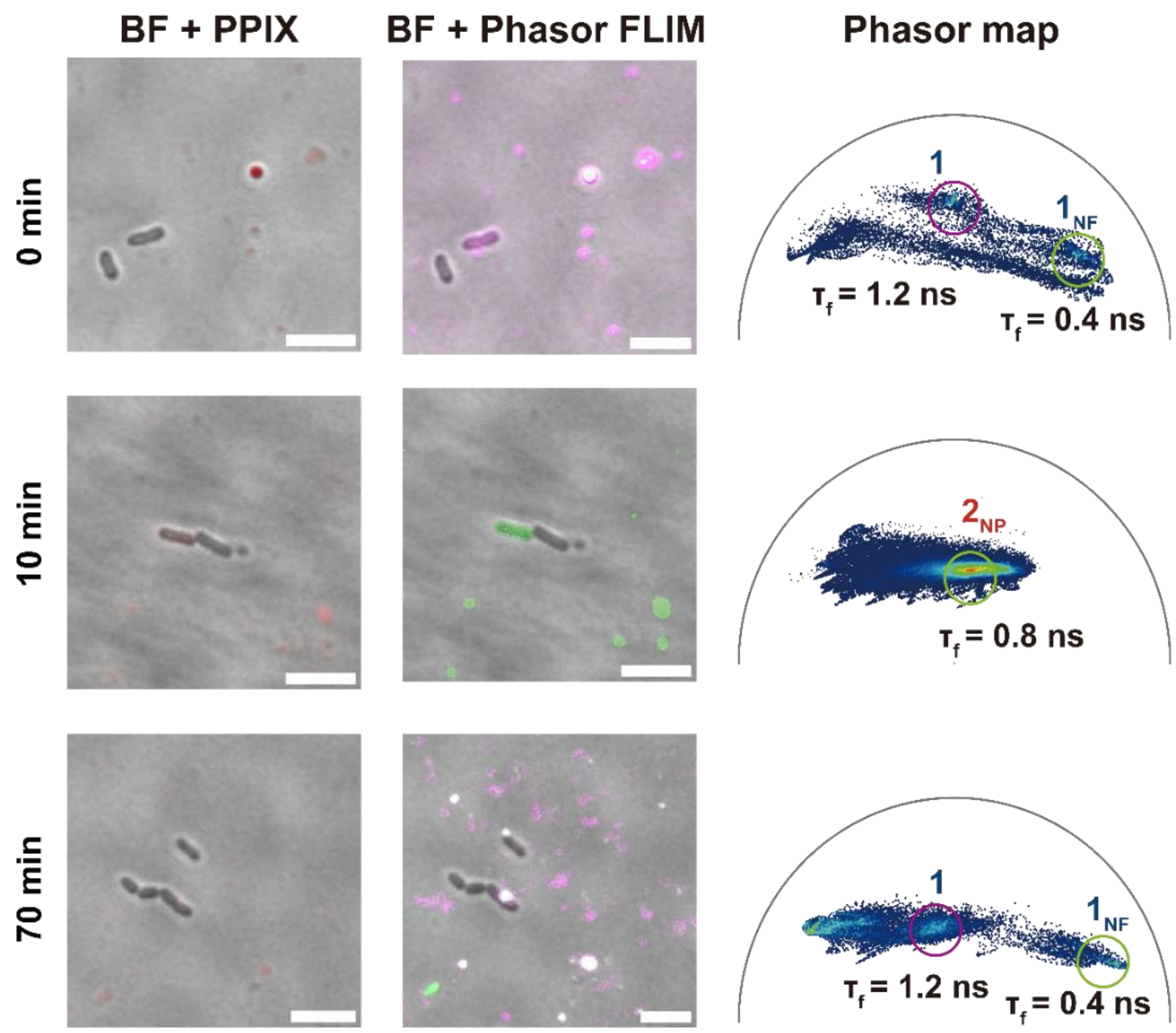

Supplementary Figure 36. CLSM merged the channel of Bright field (BF) and PPIX (left) and BF CLSM images merged with corresponding phasor FLIM images at various time points and corresponding phasor maps. The color signals are correlated with the circles in the phasor map. The magenta signal indicated monomer **1** (τ_f = 1.2 ns) and the green signal indicated assemblies **1**$_{\mathbf{NF}}$ (τ_f = 0.4 ns) and **2**$_{\mathbf{Np}}$ (τ_f = 0.8 ns). Scale bar, 5 μm. Experiments were independently repeated three times with similar results.

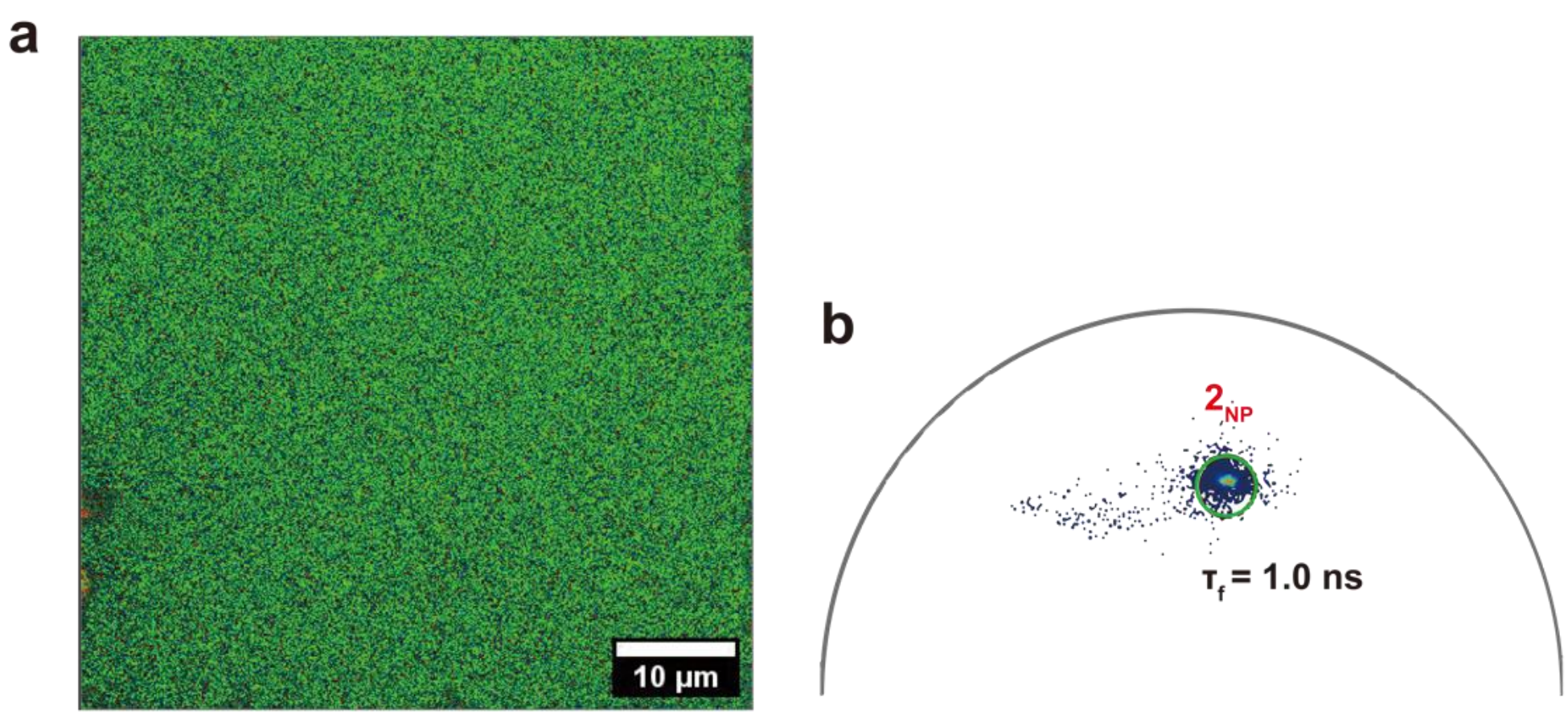

Supplementary Figure 37. a. Phasor-FLIM images by separating τ_f of **2**$_{\mathbf{NP}}$ emitted photons. b. The corresponding phasor map of **2**$_{\mathbf{NP}}$ with the addition of 0.1 mg/mL CL (Gene; *oxyR-msrA +oxyR-msrB*, activated with 100 μM H_2O_2). The fluorescence lifetime of **2**$_{\mathbf{NP}}$ is 1.0 ns. Scale bar, 10 μm. Experiments were independently repeated three times with similar results

23. Flow cytometry indicates a homeostatic synthetic biosystem

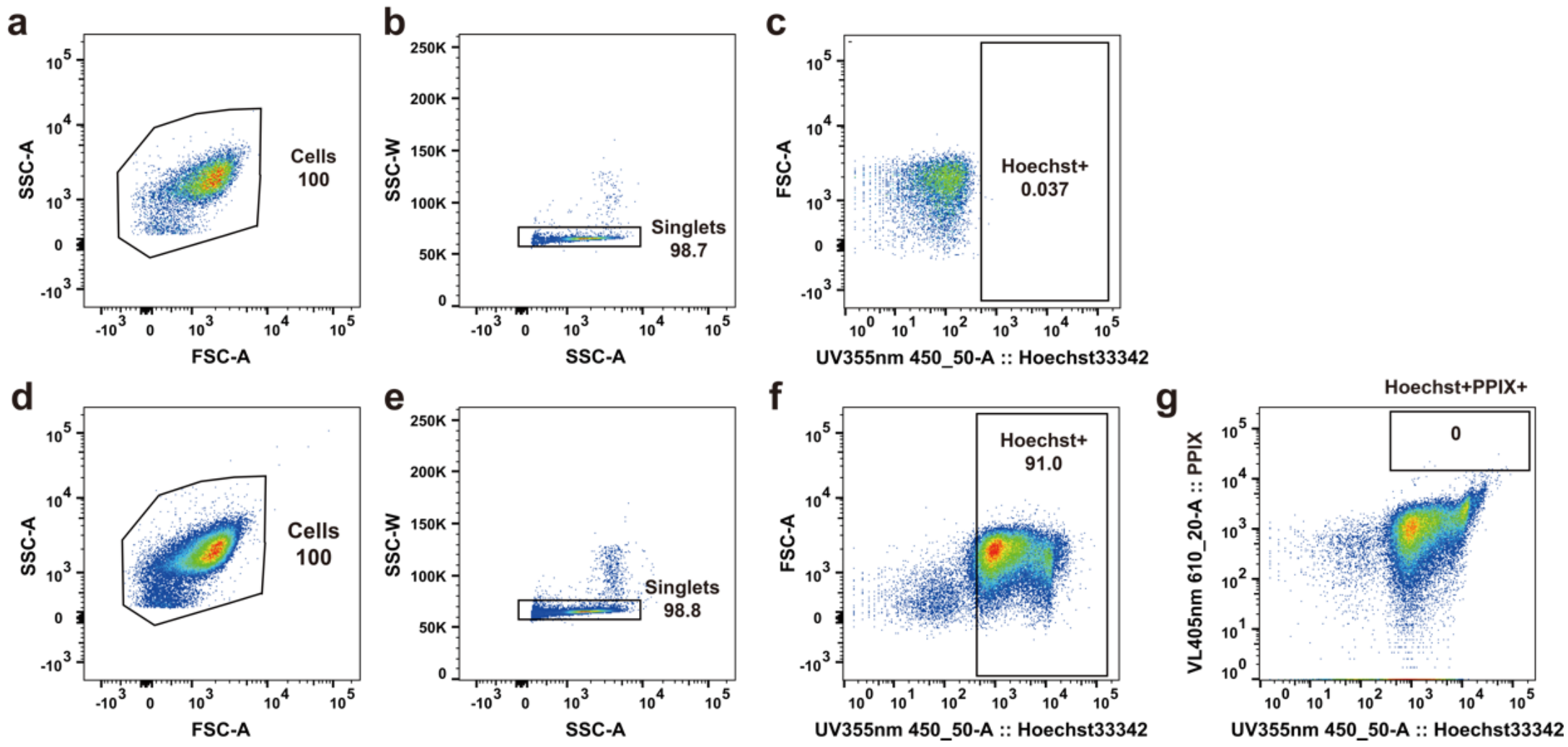

Supplementary Figure 38. Gating strategies used for flow cytometry analysis of bacteria (*E. coli oxyR-msrA+ oxyR-msrB*) uptake and *in vivo* oscillation. A, B, C represented the gating strategies applied for Hoechst-PPIX- bacteria. a. FSC-A vs SSC-A dot plots showing the gating of cells b. SSC-A vs SSC-W dot plots showing the gating of singlets (gate at cells) c. Hoechst vs FSC-A dot plots showing the gating of Hoechst+ (gate at singlets) D-G represented the gating strategies applied for Hoechst + PPIX- bacteria. d. FSC-A vs SSC-A dot plots showing the gating of cells e. SSC-A vs SSC-W dot plots showing the gating of singlets (gate at cells) f. Hoechst vs FSC-A dot plots showing the gating of Hoechst+ (gate at singlets) g. Hoechst vs PPIX dot plots showing the gating of Hoechst+ PPIX+ (gate at Hoechst+)

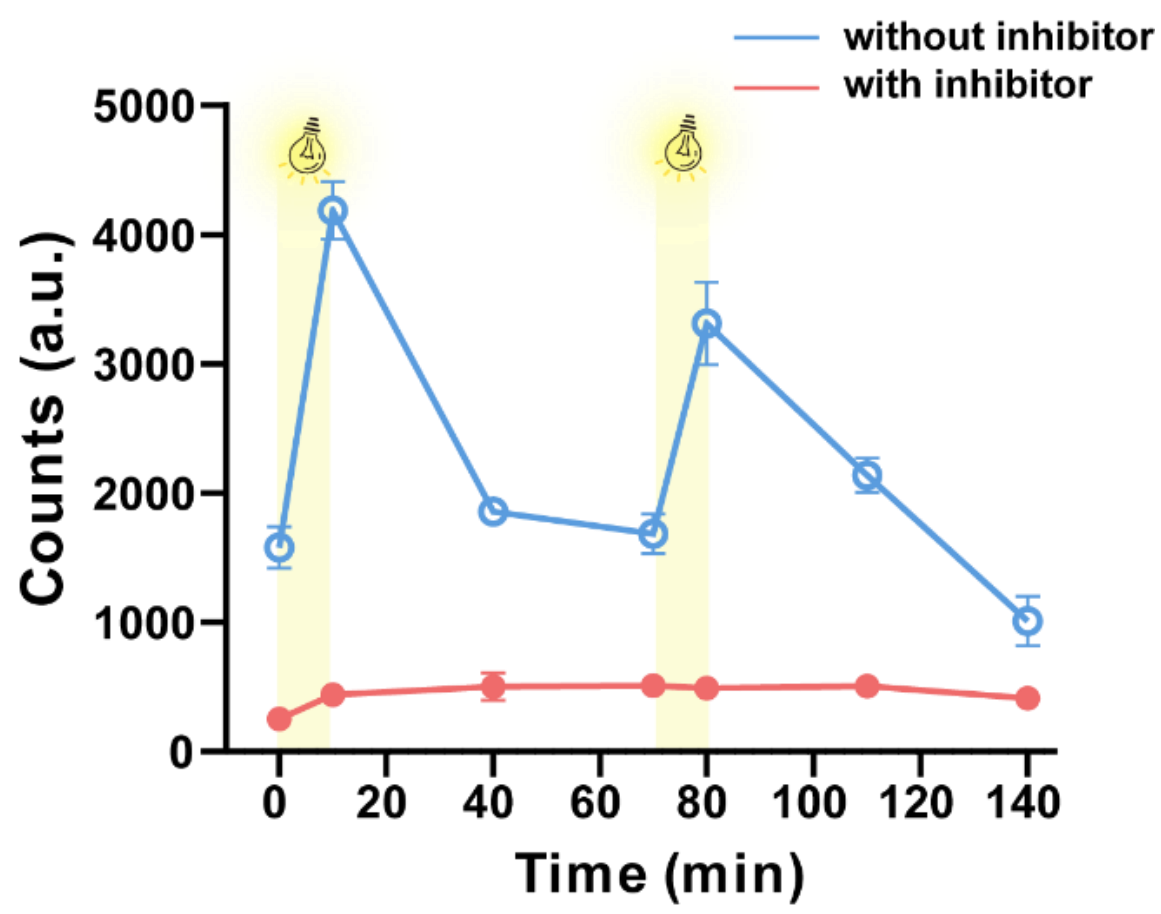

Supplementary Figure 39. Number of counts at channel Hoechst+PPIX+ at different time points with and without ATP synthase inhibitor, DCCD, quantified by flow cytometry gate at Hoechst+. Yellow shading indicates 10 min of light illumination. Data are represented as the mean ± s.d. (n= 3 independent biological replicates)

24. Bacterial lysis analysis

The solutions at each time point were combined with an equal volume of B-PER reagent (supplemented with 1 mM DTT and 1 mM PMSF). The samples were frozen in liquid nitrogen and subsequently thawed at 37 °C. This process was repeated for three cycles. Lysozyme (0.2 mg/mL) and Benzonase (50 U/mL) was then added to the samples, which were incubated at 37 °C and 450 rpm for 10 minutes. A fourfold excess of

methanol was added to precipitate the proteins, after which the samples were subjected to centrifugation at 23,000 g for 20 minutes at 4 °C. The resulting supernatant was then carefully transferred for subsequent analysis by liquid chromatography-mass spectrometry (LC-MS).

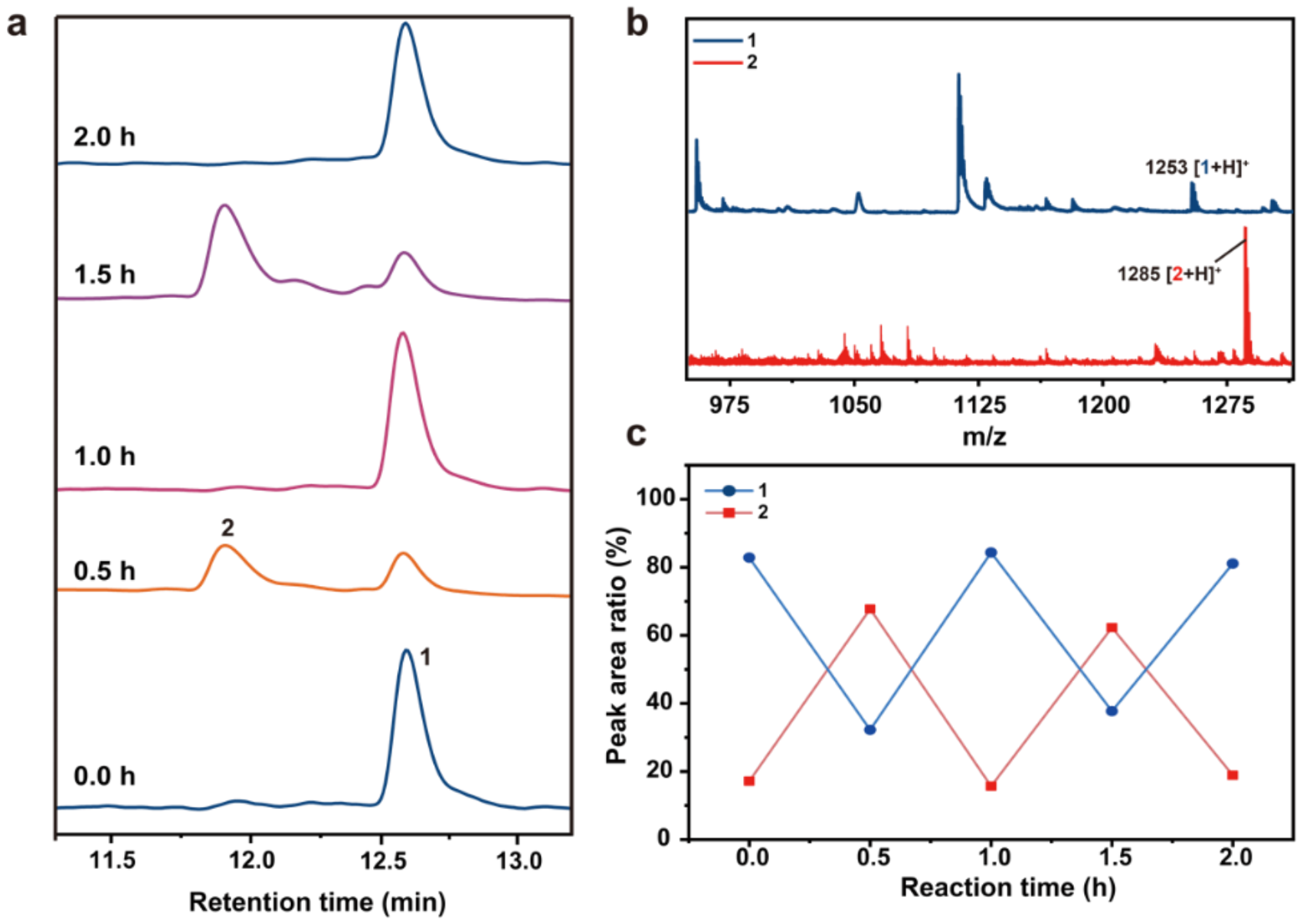

Supplementary Figure 40. a. Kinetic analysis of bacterial lysis at different time points in MeOH solutions by using HPLC-MS. b. MALDI-TOF-MS spectrum of compound **1** and compound **2**. Calculated for $[M]^+$ ($C_{62}H_{80}N_{10}O_{14}S_2$): 1252.53, found mass (m/z): 1253$[\mathbf{1}+H]^+$ Calculated for $[M]^+$ ($C_{62}H_{80}N_{10}O_{16}S_2$): 1284.52, found mass (m/z): 1285$[\mathbf{1}+H]^+$. c. Relative peak area of compound **1** and compound **2** based on the peak integration at 404 nm in two cycles.

Supplementary Table 1. Buffer compositions used in this study.

Buffer Name	Composition
AB buffer	2% methanol, 5 mM NH_4HCO_3 buffer pH=7.5
Buffer A	50 mM Tris, 300 mM NaCl, pH=7.4.
KPi buffer	42.6 mM Na_2HPO_4 x $2H_2O$, 7.4 mM KH_2PO_4, pH=7.4.
Wash buffer	50 mM Tris, 300 mM NaCl, 20 mM imidazole, pH=7.4.
Elution buffer	50 mM Tris, 300 mM NaCl, 500 mM imidazole, pH=7.4.
5X protein loading buffer	350 mM SDS, 500mM DTT, 50% Glycerol, 250mM Tris-HCL, 5% v/v β-mercaptoethanol, and 0.5% bromophenol blue dye, pH=6.8.
TBS-T buffer	20 mM Tris base, 150 mM sodium chloride, 15.24 mM HCl, 0.1% Tween-20.
Transfer buffer	25 mM Tris base, 0.192 M glycine, 20% methanol.
M9 medium	1x M9 salts, 0.4% Glucose sol., 2 mM $MgSO_4$, 0.1 mM $CaCl_2$

Supplementary Table 2. Plasmids used in this study.

Plasmid Name	Relevant Features	Source
pTrc99A[2]	GFP expressed in the presence of IPTG. pBR322 origin, AmpR.	A gift from Prof. Victor Sourjik (Max Planck Institute for Terrestrial Microbiology & Center for Synthetic Microbiology)
pET28a-msrA[3]	MsrA expressed in the presence of IPTG. pBR322 origin, KanR.	A gift from Dr. Christoph K. Winkler (University of Graz)
pET28a-msrB	MsrB expressed in the presence of IPTG. pBR322 origin, KanR.	This work
oxyR-lux	H_2O_2 sensing circuit with luciferase and its substrates as Reporter. OxyR expressed constitutively from promoter ProD, luxCDABE under promoter OxySp. pSC101 origin, KanR.	This work
oxyR-msrA	Part of feedback system with oxidative stress responses. OxyR expressed constitutively from promoter ProD, MsrA under promoter OxySp with RBS 30. pSC101 origin, KanR.	This work
oxyR-msrB	Part of feedback system with oxidative stress responses. OxyR expressed constitutively from promoter ProD, MsrB under promoter OxySp with RBS 30. pBR322 origin, AmpR.	This work

Supplementary Table 3. DNA sequences of genetic parts and plasmids used in this study. (See separated file)

Supplementary References

1 Song, N. *et al.* In situ oxidation-regulated self-assembly of peptides into transformable scaffolds for cascade therapy. *Nano Today* **38** (2021).

2 Chen, F. & Wegner, S. V. Blue Light Switchable Bacterial Adhesion as a Key Step toward the Design of Biofilms. *ACS Synthetic Biology* **6**, 2170-2174 (2017).

3 Bierbaumer, S. *et al.* Synthesis of Enantiopure Sulfoxides by Concurrent Photocatalytic Oxidation and Biocatalytic Reduction. *Angew Chem Int Ed Engl* **61**, e202117103 (2022).

Source data for Supplementary Figure 13

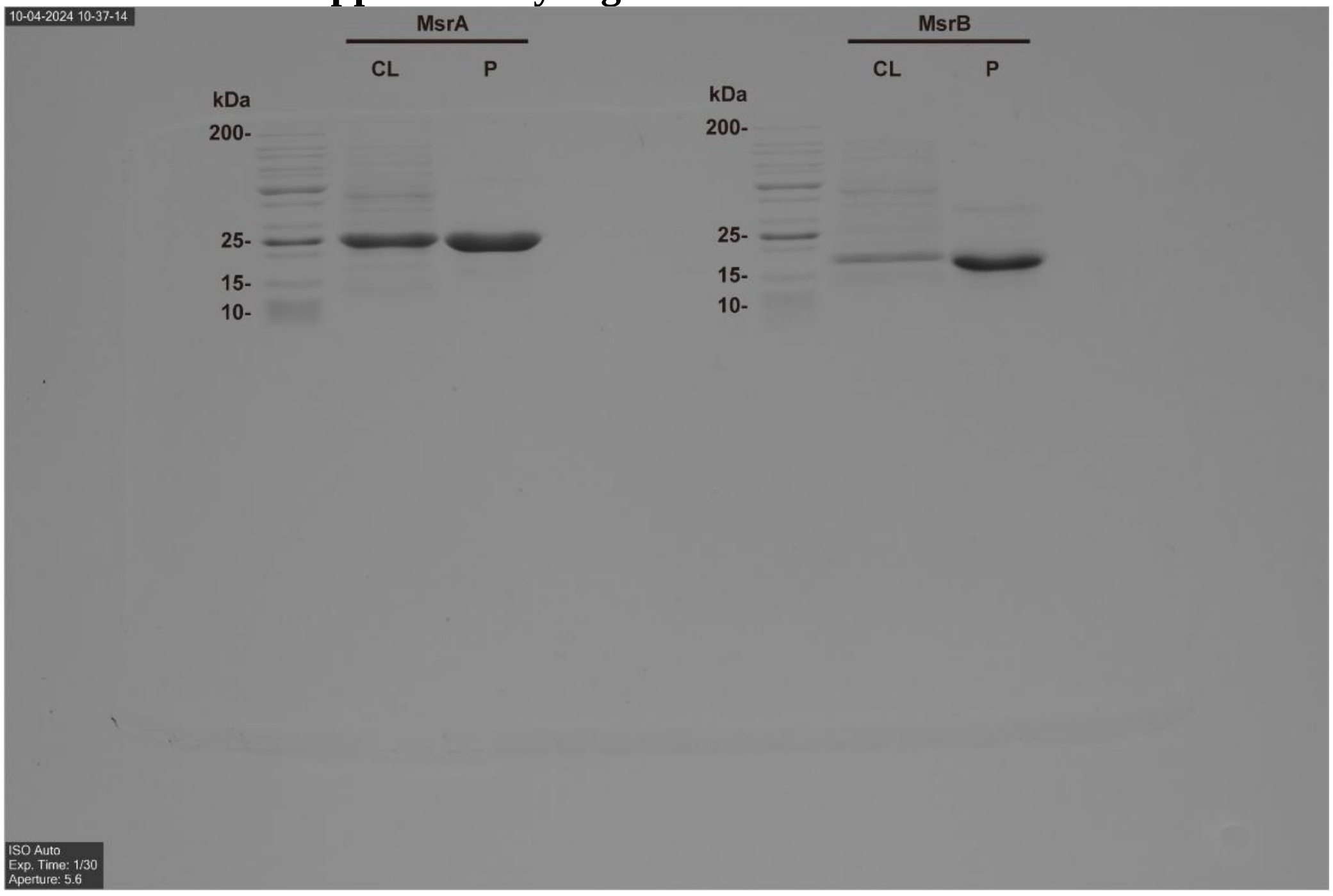

Source data for Supplementary Figure 34

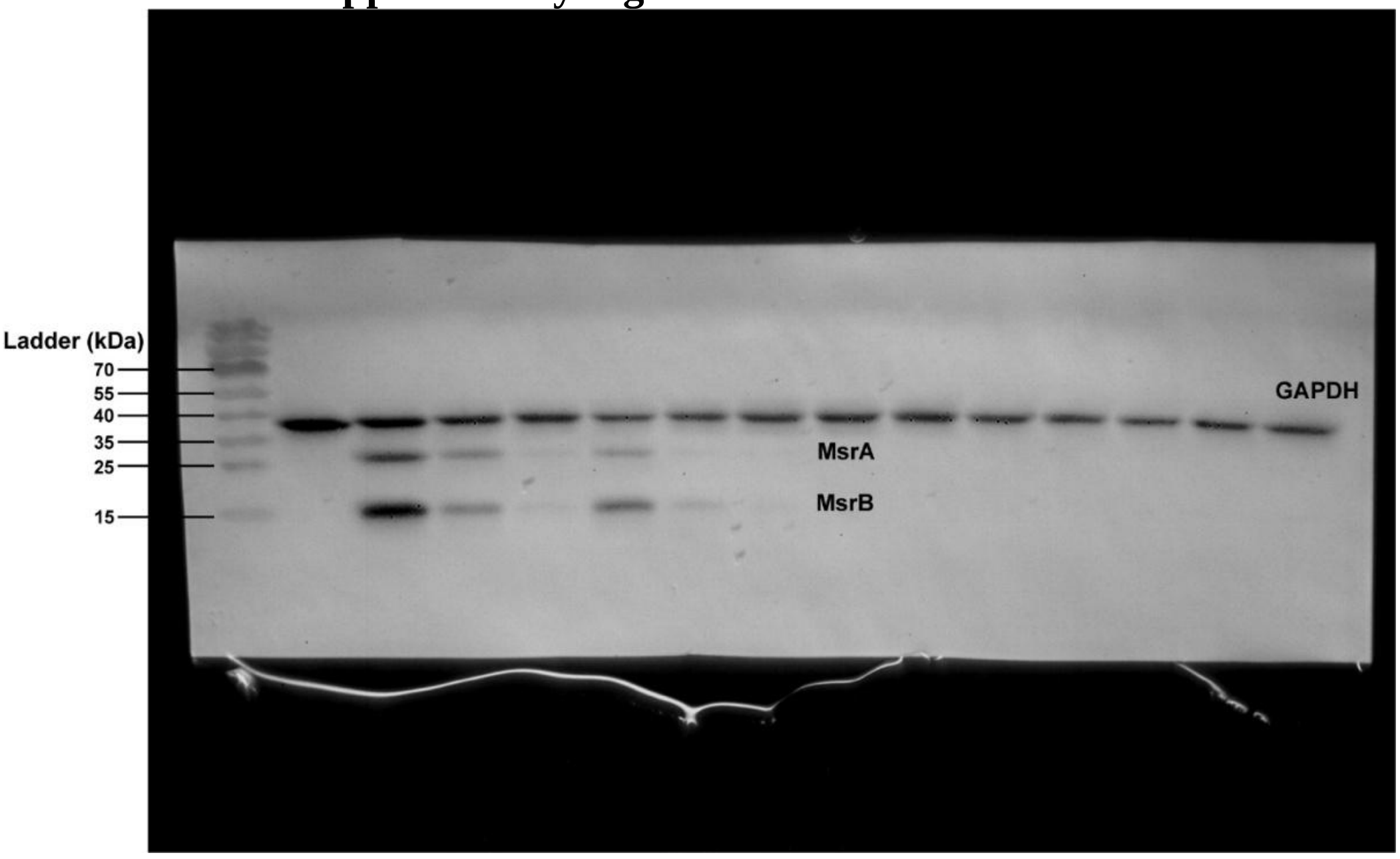

Supplement: Supplementary file 1 [file 41589_2026_2279_MOESM1_ESM.pdf]
